# Electron transfer-triggered imaging of EGFR signaling activity

**DOI:** 10.1038/s41467-022-28213-y

**Published:** 2022-02-01

**Authors:** Jie Tan, Hao Li, Cailing Ji, Lei Zhang, Chenxuan Zhao, Liming Tang, Caixin Zhang, Zhijun Sun, Weihong Tan, Quan Yuan

**Affiliations:** 1 Molecular Science and Biomedicine Laboratory (MBL), Institute of Chemical Biology and Nanomedicine, State Key Laboratory of Chemo/Biosensing and Chemometrics, College of Chemistry and Chemical Engineering, School of Physics and Electronics, Hunan University, Changsha, 410082 China; 2 The State Key Laboratory Breeding Base of Basic Science of Stomatology (Hubei-MOST) & Key Laboratory of Oral Bio-medicine Ministry of Education, School & Hospital of Stomatology, College of Chemistry and Molecular Sciences, Wuhan University, Wuhan, 430072 China; 3 Department of Chemistry, ZJU-NHU United R&D Center, Zhejiang University, Hangzhou, 310027 P. R. China

**Keywords:** Nanoparticles, Sensors and probes, Optical imaging, Growth factor signalling

## Abstract

In vivo electron transfer processes are closely related to the activation of signaling pathways, and, thus, affect various life processes. Indeed, the signaling pathway activation of key molecules may be associated with certain diseases. For example, epidermal growth factor receptor (EGFR) activation is related to the occurrence and development of tumors. Hence, monitoring the activation of EGFR-related signaling pathways can help reveal the progression of tumor development. However, it is challenging for current detection methods to monitor the activation of specific signaling pathways in complex biochemical reactions. Here we designed a highly sensitive and specific nanoprobe that enables in vivo imaging of electronic transfer over a broad range of spatial and temporal scales. By using the ferrocene-DNA polymer “wire”, the electrons transferred in a biochemical reaction can flow to persistent luminescent nanoparticles and change their electron distribution, thereby altering the optical signal of the particles. This electron transfer-triggered imaging probe enables mapping the activation of EGFR-related signaling pathways in a temporally and spatially precise manner. By offering precise visualization of signaling activity, this approach may offer a general platform not only for understanding molecular mechanisms in various biological processes but also for promoting disease therapies and drug evaluation.

## Abstract (web summary)

Here, the authors design a nanoprobe for in vivo imaging of electronic transfer, consisting of a ferrocene-DNA polymer to transfer electrons to luminescent nanoparticles, changing their optical signal. Using this probe, they map activation of EGFR signalling during tumour treatment.

## Introduction

Energy transfer in biological processes is achieved through the transfer of electrons and protons^1–3^. In essence, the transfer of energy-carrying electrons and protons is the basis of life processes, and it is inseparable from the electron transport chain^4–6^. Organisms can pass messages between biomolecules via electron transfer, thereby achieving a variety of life processes^7–10^. After signaling molecule binding, receptor or adapter protein kinases become activated by phosphorylation, thereby turning on downstream signaling pathways^11–13^. Activation of signaling pathways is directly related to electron transfer between the signaling and receptor molecules since the phosphorylation of receptor molecules is accompanied by the formation of chemical bonds and further electron transfer^5,14^. Taking tumor development as an example, compared with normal cells, tumor cells achieve extremely active electron transfer to support their proliferation, differentiation, and migration^15–18^. If the monitoring of tumor proliferation-associated signal pathway activity could be achieved, it would be possible to evaluate therapeutic behavior on this basis. This possibility has motivated the need for developing novel strategies to implement in vivo monitoring of electron transfer processes that occur in signaling pathways.

There is a variety of signaling pathways in a cell that switch on and off nimbly, and influence each other constantly, thus forming a complex network^13,19,20^. Because the activity of these signaling pathways changes rapidly, a rapid, sensitive monitor of dynamic changes in signal transduction is required. Techniques have been well developed to measure electron transfer between biological molecules, such as nuclear magnetic resonance spectroscopy and electrochemical methods^21–23^. Optical methods are mostly characterized by high temporal and spatial resolution, non-contact detection, and high-sensitive visualization based on machine vision^11,24–28^. Specifically, the development of quantum biological electron tunneling spectroscopy enables imaging of electron transfer in live cells, while it is not easily applicable for in vivo testing^29^. Hence, if an optical probe is designed to respond to electron transfer, it would be possible to realize dynamic monitoring of electron transfer during target signal transduction in vivo^30,31^.

Here we designed a highly sensitive and specific nanoprobe that enables in vivo imaging of electronic transfer. Specifically, by imaging electron transfer in biochemical reactions, we determined that the change of probe signal with electron transfer was due to the electron transfer from biochemical reactions into the probe material. At the cellular level, the activation of EGFR-related signaling pathways was visualized by electron transfer-triggered imaging. Additionally, we further developed this strategy to enable monitoring of disease processes during the dynamic procedure of tumor treatment. EGFR activation was positively correlated with tumor progression, thus enabling the evaluation of therapeutic progress^17,18^. Since there are diverse signaling pathways in life activities, this electron transfer-triggered imaging approach provides a general platform, not only for understanding molecular mechanisms in various biological processes but also for promoting disease therapies and drug evaluation.

## Results

### Design of ETTE nanoprobes for electron transfer-triggered imaging

**Fig. 1:**
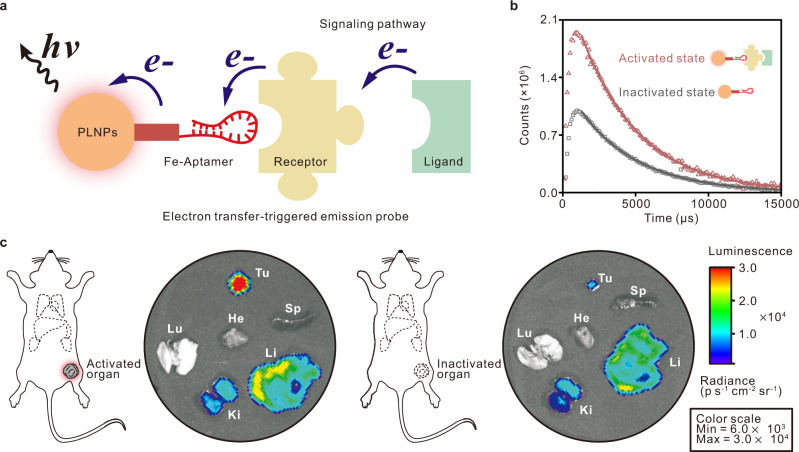
Schematics of electron transfer-triggered imaging. **a** Schematic of ETTE nanoprobe for electron transfer-triggered imaging (not to scale). **b** Time-resolved fluorescence curve of ETTE nanoprobes in water and in solution with glucose oxidase and glucose. The experiments were repeated independently three times. **c** Afterglow images of organs and tumors at 48 h after intravenous administration of ETTE nanoprobes without and with MutT homolog 1 (MTH1) siRNA as a therapeutic reagent, respectively. Tu tumor, Sp spleen, Li liver, Ki kidney, Lu lung, He heart. Color scale: 6.0 × 10^3^–3.0 × 10^4^.

To achieve electron transfer-triggered imaging of target signaling pathways in vivo, we designed an electron transfer-triggered emission nanoprobe (ETTE nanoprobe) containing persistent luminescence nanoparticles (PLNPs) for generating afterglow signals^32^ and ferrocene-DNA polymer chains for directional electron transfer^33–35^. As schematically illustrated in Fig. 1a, the strategy for electron transfer-triggered emission imaging is based on the electron transfer chain between our ETTE nanoprobe and the ligand of target molecule. When ligands of the target molecules are present, the electron can transfer from the ligand to the persistent luminescence nanoparticle through the ferrocene-DNA polymer chain, leading to a change of afterglow signal (Fig. 1b). As a model, the in vivo imaging of electron transfer in EGFR signaling pathways was assessed in a mouse with lung cancer. The map of EGFR-related signaling pathway activity in mouse organs was observed using ETTE nanoprobe (Fig. 1c). The afterglow signal in the tumor region was significantly higher than that in other organs, showing that the tumor region exhibited the most active EGFR-related signaling pathway. For the therapeutic group, the volume of tumor decreased sharply, resulting in a declined afterglow signal, thus indicating the decrease of EGFR-related signal pathway activity.

### Afterglow imaging and quantification of electron transfer

**Fig. 2:**
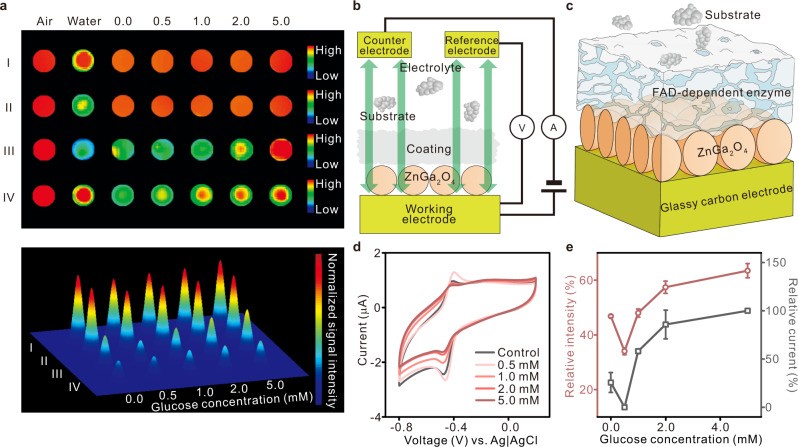
Afterglow imaging and quantification of electron transfer. **a** Afterglow image (upper) and readout signal intensity mapping image (lower) of persistent luminescence nanoparticles (I, Sr_2_MgSi_2_O_7_:Eu,Dy, color scale: 2.50 × 10^7^–1.44 × 10^9^; II, SrAl_2_O_4_:Eu,Dy, color scale: 2.50 × 10^7^–1.44 × 10^9^; III, ZnGa_2_O_4_:Cr, color scale: 2.00 × 10^7^–1.10 × 10^8^; IV, Zn_3_(PO_4_)_2_:Mn^2+^,Ga^3+^, color scale: 1.25 × 10^7^–2.00 × 10^10^) under different conditions (air, water, and GOD solution with 0.0–5.0 mM glucose) at 1.0 s after white light excitation. **b** Schematic of the three-electrode electrochemical set-up used. **c** Schematic of the ZnGa_2_O_4_/GOD coating on a glassy carbon electrode in glucose-containing electrolyte. **d** Typical voltammograms showing oxidation and reduction peaks of phosphate-buffered solution (PBS) solution of glucose at various concentrations (0.0–5.0 mM) on a fresh ZnGa_2_O_4_/GOD-coated electrode. **e** Extracted relative afterglow intensity mean values (red circles) from the afterglow images **a** and extracted reduction peak current mean values (gray squares) from the voltammograms versus the concentration of glucose (*n* = 3 independent electrodes). Error bars in all graphs represent the s.d. of the mean values.

It has been reported that the molecular structure or charge distribution of a luminescent probe can be influenced by environmental factors, causing a change of luminescence signals^36–38^. The afterglow signal of persistent luminescence materials was investigated to establish whether it would be affected by chemical reactions in the surrounding environment as well. As the enzyme cofactor, flavin adenine dinucleotide (FAD) is a crucial molecule in core metabolic pathways and reflects the growth state of the cells^39,40^. Taking the catalytic reaction of FAD-dependent glucose oxidase (GOD) and glucose as an example, the afterglow signals of four common persistent luminescence nanoparticles were detected in a quantitative catalytic reaction environment. The afterglow intensities of these materials were moderate to low in the GOD solution and gradually increased when glucose was added to the solution (Fig. 2a). As a result, the afterglow signal of all these persistent luminescence materials varies with the quantitative catalytic reaction of GOD and glucose, confirming that the afterglow signal can be used to characterize the process of biochemical reactions. The 3D map of the afterglow signal (Fig. 2a below) clearly demonstrates that among the four common persistent luminescence materials, ZnGa_2_O_4_ is the most affected, indicating its high sensitivity to electron transfer. Therefore, ZnGa_2_O_4_ was selected as the system that balances the background intensity with the retention of sensitivity.

The electrochemical activity of ZnGa_2_O_4_ was further characterized in different concentrations of glucose solution to determine the relationship between the afterglow signal and the electron transfer. ZnGa_2_O_4_ and GOD were sequentially coated on the electrode surface, and the electrode was employed for cyclic voltammetry in a solution containing different concentrations of glucose (Fig. 2b, c). While the GOD coating resulted in total passivation (Supplementary Fig. 1), the ZnGa_2_O_4_/GOD coating exhibited obvious oxidation and reduction peaks of GOD (Supplementary Figs. 2 and 3), in part due to ZnGa_2_O_4_-induced electron transfer. The electrochemical performance of ZnGa_2_O_4_/GOD coating was detected in glucose solution (Fig. 2d), the reduction peak currents gradually decreased with glucose addition, that is, the consumption of oxidized GOD provides routes for the electronic transduction occurring at the ZnGa_2_O_4_/GOD surface. The afterglow signal was demonstrated to be proportional to the reduction peak current by quantitative analysis, leading to a better understanding of the essential mechanisms associated with the afterglow signal and the electron transfer (Fig. 2e).

### Mechanism of electron transfer-triggered imaging

**Fig. 3:**
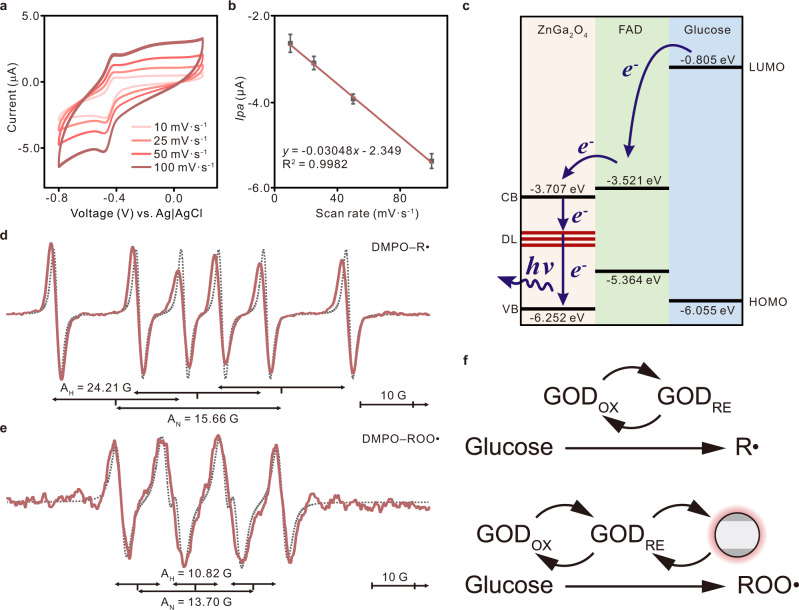
Electron transfer between ZnGa_2_O_4_ and target molecules. **a** Voltammograms of GOD/ZnGa_2_O_4_-coated electrodes of a PBS solution at different scan rates (10–100 mV·s^−1^). **b** Extracted oxidation peak current (ip) mean values (circles) from the voltammograms shown in a plotted versus the scan rate (*n* = 3 independent electrodes). Error bars in all graphs represent the s.d. of the mean values. **c** The energetic structure of the system, as determined by density functional theory (DFT) calculations (Supplementary Figs. 3–6). FAD, flavin adenine dinucleotide; CB, conduction band; DL, defect level; VB, valence band; LUMO, lowest unoccupied molecular orbital; HOMO, highest occupied molecular orbital. **d** EPR spectrum of glucose and GOD mixture observed at 295 K for the 5,5-dimethyl-1-pyrroline-N-oxide (DMPO)/alkyl radical adducts. The dashed line is a simulated spectrum using parameters reported in the text. The inhomogeneous line widths for the spin-adduct have been adjusted to 0.4 G to take into account the broadening caused by the relaxation effect. **e** EPR spectrum of glucose, GOD, and ZnGa_2_O_4_ mixture observed at 295 K for the DMPO/peroxyl radical adducts. The dashed line is a simulated spectrum using parameters reported in the text. The inhomogeneous line widths for the spin-adduct have been adjusted to 0.8 G to take into account the broadening caused by the relaxation effect. **f** A higher alkyl radical EPR intensity is seen when the glucose is treated with GOD **d**. When the glucose is treated by GOD with ZnGa_2_O_4_, a higher peroxyl radical EPR intensity is observed (**e**).

The electrical signals during the reaction were characterized by electrochemical methods, and the voltametric peak current as a function of the scan rate was evaluated (Fig. 3a). As schematized in Fig. 3b, the peak current is proportional to the scan rate, indicating that the redox process occurring on the ZnGa_2_O_4_/GOD coating is surface adsorption-limited rather than diffusion-limited^41^. Thus, it is reasonable to speculate on the electron transfer between GOD and ZnGa_2_O_4_ adsorbed on the surface. The corresponding band diagrams of ZnGa_2_O_4_, glucose, and FAD, a cofactor of GOD, were calculated to investigate the electron transfer from an electron donor to an acceptor (Fig. 3c and Supplementary Figs. 4–7)^42,43^. For glucose with high electrochemical energy levels, electrons in its LUMO energy level will tunnel into ZnGa_2_O_4_ with the assistance of FAD-dependent enzymes. The Fermi level of ZnGa_2_O_4_ will increase via electron transfer and lead to the increment of electrons in the defect level by charge separation and trapping, thus eventually enhancing the afterglow emission. As a consequence, the electrons transferred from glucose to ZnGa_2_O_4_ can recombine to the electronic ground state via radiative pathways, leading to an enhanced emission that is highly consistent with the observation in afterglow images.

The catalytic reaction between GOD and glucose was also characterized by electron paramagnetic resonance (EPR) spectroscopy to investigate the performance of ZnGa_2_O_4_-mediated electron transfer in this reaction (Fig. 3d, e). The A_N_ values of DMPO/R radical adducts (Fig. 3d) and DMPO/ROO radical adducts (Fig. 3e) were 15.66 G and 13.70 G, and their A_H_ values were 24.21 G and 10.82 G, comparable to spin adducts of DMPO/alkyl and DMPO/peroxyl, respectively^44,45^. The EPR spectrum of the glucose and GOD mixture displayed large numbers of alkyl radicals, while peroxyl radicals were generated with the addition of ZnGa_2_O_4_ (Fig. 3e), suggesting the transition of radical signals from alkyl radicals to peroxyl radicals after exposure of ZnGa_2_O_4_ (Supplementary Figs. 8–10). It can be concluded that with both ZnGa_2_O_4_ and GOD present, glucose will be oxidized to peroxyl radicals rather than alkyl radicals, further indicating that electron transfer occurs between GOD and ZnGa_2_O_4_.

### Optimization of the ETTE nanoprobe

In an ideal solution environment, it was demonstrated that ZnGa_2_O_4_ can produce obvious afterglow signal, varying with the number of transferred electrons. By calculating the photoluminescence efficiency and the electron-hole recombination rate of the probe (see Supplementary Information, section ‘Electron tunneling probability calculation’), it can be concluded that the efficiency of photoluminescence is proportional to the number of electron-hole pairs of the material. Since ZnGa_2_O_4_ is a *p*-type semiconductor material, where the hole concentration is much higher than the electron concentration, it can be considered that the effect of hole concentration change on the radiative recombination rate is negligible. Thus, the photoluminescence intensity of ZnGa_2_O_4_ is positively correlated with its electron concentration. To optimize the electron transfer-induced luminescence efficiency of the probe, it is necessary to increase the number of transferred electrons between the target molecule and ZnGa_2_O_4_ in unit time. For tunneling through a one-dimensional barrier in a weakly coupled donor-acceptor system, the electron transfer rate *k*_*ET*_ is shown in formula (1).

1 $${k}_{{ET}}=\frac{2\pi }{{{\hslash }}}{\left|{T}_{{DA}}\right|}^{2}\left(F.C\right)={A}^{2}{e}^{{-R}_{{DA}}\beta }(F.C)$$

where ℏ is the reduced Planck constant, ℏ = *h*/2π, *T*_*DA*_ is the electronic tunneling matrix element between donor and acceptor localized states. (F.C.) is the Franck-Condon factor which is determined by the activated or tunneling nuclear processes coupled to the transfer^46^. Formula (1) gives the calculation method of electron tunneling probability *T*_*AD*_^2^, where *R*_*DA*_ is the distance between donor and acceptor, and *β* is the barrier height. It can be seen that the probability of electron tunneling can be greatly enhanced by reducing the barrier height and the distance between the target molecule and the probe^47^.

To induce electron transfer from the signaling pathway to the probe, molecular “wires” with molecular targeting ability were assembled on the surface of the material to close the distance between the target molecule and the probe. Because ferrocene structure exhibits high electrical conductivity and low energy barrier^35^, an aptamer-ferrocene polymer was a promising candidate for molecular wires (see Supplementary Information, section ‘Synthesis of Fe-base’, and Supplementary Figs. 11–20). An increase in the number of ferrocene moieties can reduce the barrier height of the aptamer-ferrocene polymer by extending the conjugate field, and, on the other hand, lengthen the distance between the target molecule and the probe. Here, ETTE nanoprobes with different lengths of molecular wires were constructed via the phase transfer reaction to establish how the number of Fe-bases affects the electron transfer (Supplementary Table 1, Supplementary Fig. 21–32). Energy level distributions of ZnGa_2_O_4_ and ferrocene moieties in the ETTE nanoprobe were calculated (Supplementary Fig. 33), confirming that electrons in the defect level of ZnGa_2_O_4_ will transfer to the ferrocene moieties and quench the afterglow signal^48^. It can be concluded that within the ETTE nanoprobe, the higher the electron tunneling probability, the lower the afterglow emission. Fluorescence spectrum of ETTE nanoprobe with varying Fe-base modifications was obtained and the probe with nine Fe-bases exhibited the lowest afterglow signal (Supplementary Figs. 34–37). As a result, the ETTE nanoprobe with nine Fe-bases was selected as the optimized system that balances the reduction of barrier height with the retention of a close distance.

### Electron transfer-triggered imaging of EGFR activity in living cells

EGFR signaling pathway activity is associated with tumor cell proliferation, angiogenesis, tumor invasion, metastasis, and inhibition of apoptosis^17,18^. Key molecular EGFR can self-phosphorylate for activating downstream signal pathways located in cells, such as mitogen-activated protein kinase (MAPK) and c-Jun N-terminal kinase (JNK) pathways. Hence, selective detection of the activity of EGFR-related signal pathways can help to understand the progression of tumor development. To map EGFR activity in complex biological samples, it is necessary to achieve the specific detection of target signal pathway activity in numerous active signal pathway networks. Thus, a molecular wire with target recognition was needed to satisfy the requirements. Known as a chemical antibody, the nucleic acid aptamer was a promising candidate for target recognition, and an EGFR-targeting aptamer was modified with 9 Fe-bases to form a specific molecular wire. This molecular wire was then functionalized on the surface of ZnGa_2_O_4_ to achieve specific electron transfer imaging of EGFR activity.

**Fig. 4:**
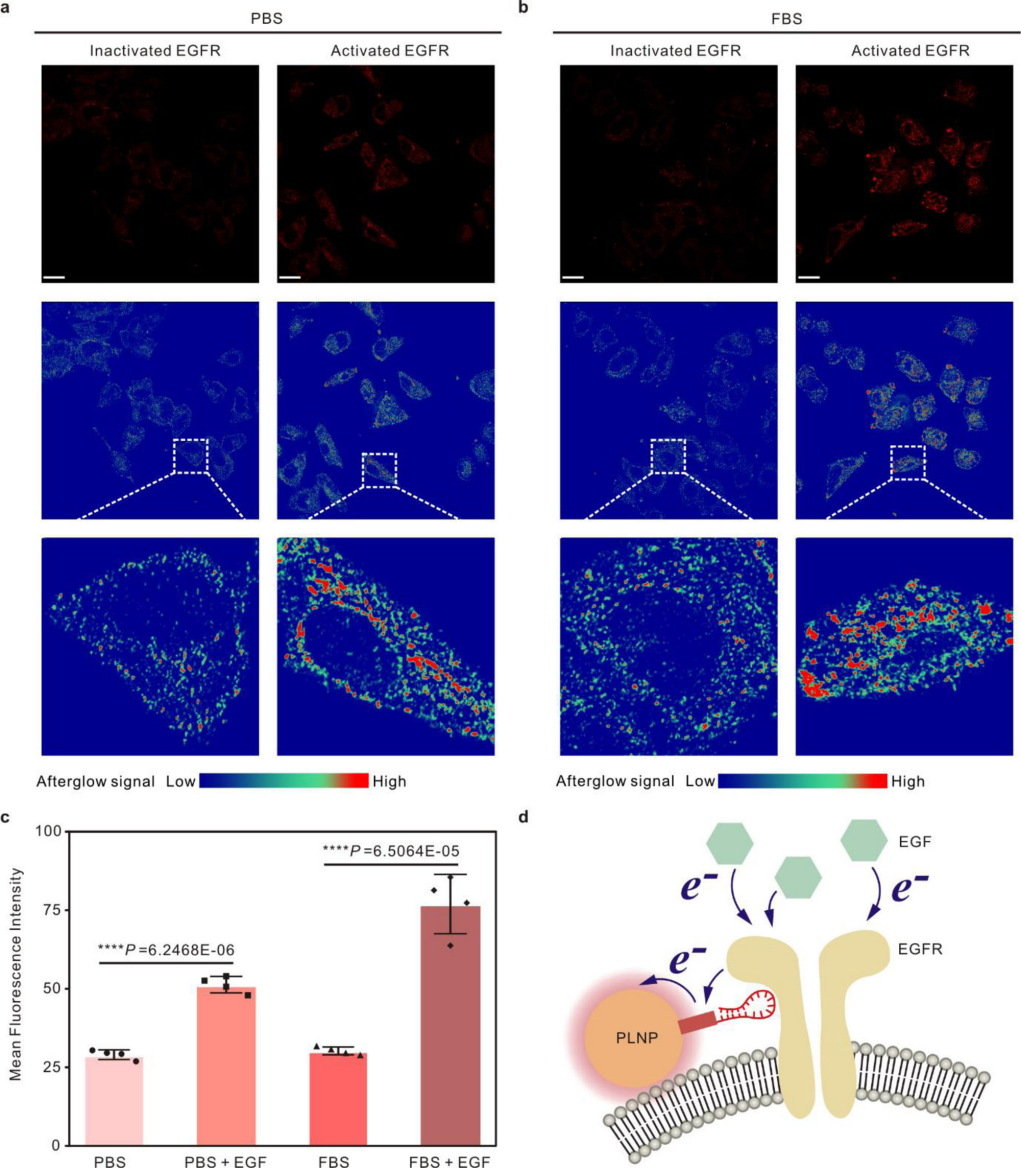
EGFR signaling pathway-related electron transfer imaging in living cells. All cells were incubated with ETTE nanoprobes and DAPI for imaging (Supplementary Fig. 25). Afterglow images and corresponding pseudo-color images of ETTE nanoprobes from A549 cells incubated without or with EGF in PBS (**a**) and in 10% fetal bovine serum (FBS) culture medium (**b**). Higher intensities reflect higher EGFR signaling activity. Color scale (Δ*F*/*F*_0_): 0–1.5. Scale bar, 20 μm. The laser of 635 nm and 365 nm were used as the excitation sources, afterglow signal from the ETTE probe was obtained at 1.0 s after excitation. The imaging experiments were repeated independently three times and similar results were obtained. **c** The bar graph shows the relative afterglow intensities of ETTE nanoprobes. Data are presented as the mean values ± s.d.; unpaired two-tailed Student’s *t*-test; *n* = 4 independent experiments. **d** Schematic of EGFR signaling pathway-related electron transfer-triggered emission of ETTE nanoprobes (not to scale).

The imaging of EGFR signaling pathway activity was performed in A549 lung cancer cells using the modified ETTE nanoprobes as imaging agents (Supplementary Fig. 38). The cultured lung cancer cells were first preincubated with ETTE nanoprobes in PBS, then exposed to EGF to activate the EGFR signaling pathway. In EGFR-activated cells and in EGFR-inactivated cells, comparable fluorescence signals could be observed (Supplementary Figs. 39–40), referring to the similar EGFR-targeting capability of ETTE nanoprobes. In the case of the afterglow signal from the EGFR-targeting ETTE nanoprobe (Fig. 4a), the pseudo-color image showed a weak afterglow signal in PBS buffer, due to the feeble life activity and the inactivated EGFR signaling pathway in the cells. After activation of the EGFR pathway, the afterglow signal displayed a significant enhancement, confirming that the probes have intensive afterglow-signal-generation efficiency in EGFR-activated cells and show high sensitivity to the EGFR activity. To simulate the complex in vivo environment, the above tests were repeated in the serum, and the enhancement of the afterglow signals could also be observed in EGFR-activated cells (Fig. 4b). In both PBS and FBS environment, the EGFR signaling pathway in cells was activated with the addition of EGF, and the intracellular afterglow signal was also enhanced obviously. Statistically, the relative signal intensity of these images was calculated. The relative intensity of the afterglow signal rose from 20 to 60% after EGF exposure under PBS condition, while 80% of relative intensity value could be obtained at EGF stimulation in the serum (Fig. 4c). These observations demonstrate that the ETTE probe could achieve electron transfer-triggered imaging for the EGFR signaling activity. EGFR and phosphorylated EGFR (P-EGFR) in A549 cells were characterized using western blot analysis. As observed in western blot results, the band for P-EGFR was clearly displayed in the EGF-treated cells (Supplementary Fig. 41), fitting well with the afterglow signal of the ETTE probe. The sensitivity of ETTE probes was evaluated in PBS and FBS environment. To measure the sensitivity of ETTE probes, EGF was progressively increased, which increased the afterglow intensity in EGFR-expressing A549 cells, yielding an EC_50_ (concentration for 50% maximal effect) of about 20 ng·mL^−1^ both in PBS and in FBS condition (Supplementary Figs. 42–45). The ETTE probe achieves comparable sensitivity with other strategies such as fluorescence resonance energy transfer^49^ and surface-enhanced Raman scattering^50^. The longest timescales of the ETTE probe can reach almost 10 min, making it possible to obtain kinetic information (Supplementary Fig. 46). Furthermore, molecular level validation of the ETTE probe for EGFR dependent signal mapping was performed on the EGFR-knocked out A549 cells. For EGFR-knocked out A549 cells, ETTE probes were found insensitive to the stimulation of EGF (Supplementary Figs. 47–50), suggesting that the afterglow signal of the ETTE probe is EGFR dependent. To confirm that the signal of the ETTE probe is indeed EGFR specific, EGFR signaling activity in some EGFR-expressing cells, such as A549 cell, CAL-27 cell, and MDA-MB-231 cell, were tested with EGF stimulation and EGFR tyrosine kinase inhibition^51,52^ (Supplementary Fig. 51). As a result, stimulation of EGF was found to activate the signals of ETTE probes significantly, while the afterglow signals in EGFR-expressing cells were completely blocked by co-application of afatinib, an EGFR tyrosine kinase inhibitor, indicating specific responses of the ETTE probe^53,54^ (Supplementary Figs. 52–55). Altogether, the results demonstrate that the afterglow signal from the ETTE probe is specific to EGFR and can be used as a reliable, non-invasive method to measure EGFR-related signaling activity in live cells. Flow cytometry has been used to analyze cellular signaling events, however, this platform always gives relatively static results and cannot map the signaling events dynamically in living cells^55,56^. The ETTE nanoprobe can possess a dynamic monitoring capability of the EGFR signaling pathway during cancer cell division, as depicted in Supplementary Fig. 56 and Supplementary Movie 1. These phenomena are possibly due to the electron transfer from activated EGFR to the probe (Fig. 4d). During a fluorescence emission process, charging of the probe can be achieved by light irradiation, enabling bright fluorescent signals^57^. Drowned by the strong fluorescent signals, the slight optical signal change caused by electron transfer in organisms can thus not be observed. For the ETTE probe, some of the electrons/holes created by optical excitation would be trapped at specific defects and enable the probe to store the excitation light^32^. Different from fluorescent signals, afterglow signals from the ETTE probe were produced by the slow release and radiative recombination of carriers from trapping defects. As a time-dependent decayed signal, afterglow intensity mostly relies on the stored electrons that would be affected by the electron transfer between biomolecules via electron acception/donation. As a consequence, the mapping of EGFR activity in living cells can be achieved by evaluating the afterglow emission of this optimized ETTE nanoprobe.

### Electron transfer-triggered imaging of EGFR activity in vivo

**Fig. 5:**
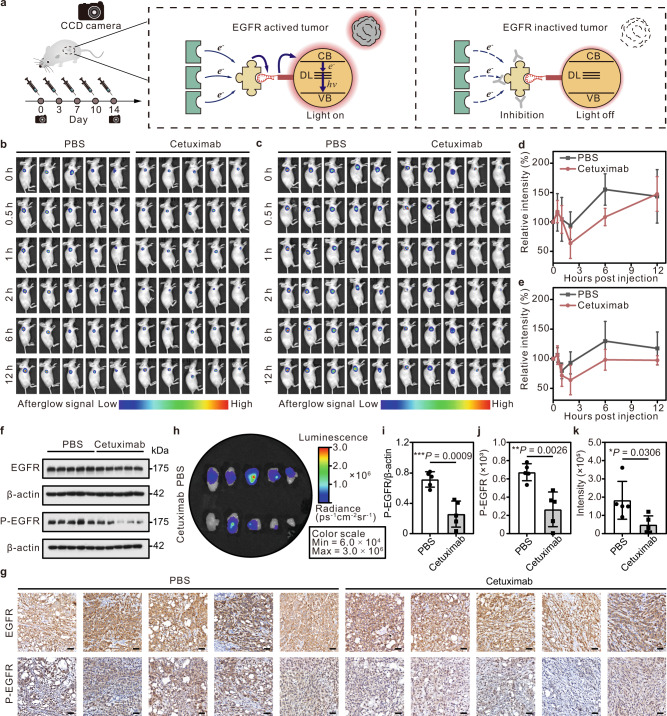
EGFR signaling pathway-related electron transfer-triggered imaging in vivo during EGFR inhibition. **a** Schematic of electron transfer-triggered imaging of EGFR-inhibited mice with a single established A549 tumor. Mice with 50 mm^3^ subcutaneous tumors were administered with PBS or cetuximab intraperitoneally twice per week. **b**, **c** Afterglow images of A549 xenograft mice treated with PBS or cetuximab at day 0 (**b**) and day 14 (**c**). The afterglow imaging was performed after 1.0 s of white light excitation. The afterglow signal intensities are displayed as color maps, color scale: (**b**) 3.5 × 10^5^–3.0 × 10^6^, (**c**) 6.0 × 10^4^–1.0 × 10^6^. **d**, **e** Corresponding quantitative data analysis of relative total radiance in tumor area of mice shown in **b** (**d**) and **c** (**e**). Data presented as mean values ± s.d., *n* = 5 mice. **f** Western blot analysis on expression of EGFR, P-EGFR, and β-catenin protein in tumors after the final treatment. A representative image of five biologically independent samples from each group is shown. **g** IHC analysis of tumor sections. EGFR and P-EGFR in the tumor sections of each group were observed by IHC analysis. Scale bar: 50 μm. A representative image of five biologically independent samples from each group is shown. **h** Afterglow images of harvested tumors from the tumor-bearing mice treated with PBS or cetuximab after 14 days treatment. Color scale: 6.0 × 10^4^–3.0 × 10^6^. **i**, **j** Quantitative analysis of P-EGFR in tumor sections observed by western blot analysis (**i**) and IHC analysis (**j**). **k** Quantitative analysis of afterglow intensity in the tumor site. Data are presented as the mean values ± s.d.; unpaired two-tailed Student’s *t*-test; *n* = 5 mice.

To further clarify the EGFR monitoring capacity of the ETTE probes in vivo, mice from the EGFR-noninhibited and EGFR-inhibited group were investigated (Fig. 5a), respectively. As a clinical used EGFR inhibitor, cetuximab can specifically bind to the EGFR with high affinity and block the normal function of the receptor^58^. The in vivo electron transfer-triggered imaging of EGFR was therefore performed using a murine model of lung cancer that was susceptible to cetuximab in vivo^59^ (see Supplementary Information, section ‘EGFR Inhibition Using Cetuximab’). The inhibition of EGFR was performed using cetuximab in tumor-bearing mice and was characterized by ETTE probe at day 0 and day 14, respectively (Supplementary Figs. 57–61). Both real-time afterglow results showed that the afterglow intensity of the EGFR-inhibited group was significantly decreased after injection of cetuximab compared with the EGFR-noninhibited group (Fig. 5b, c). The correlation curve of afterglow signal intensity versus injection time showed that the relative signal intensity fluctuated above 100% for the PBS-treated group (Fig. 5d, e gray line), while it decreased to 60% for the cetuximab-treated group (Fig. 5d, e red line). For the EGFR-inhibited group, the afterglow signal was dimmer after 1 h post injection of cetuximab and reached a minimum at 2 h at the tumor site within the extended observation time window. The electron transfer-triggered images of EGFR-noninhibited and EGFR-inhibited mice suggest that the inhibition of EGFR could be vividly observed using our ETTE probe. To ensure the activity of EGFR signaling pathway, the expression of EGFR and P-EGFR in tumor tissues was characterized by traditional western blot and immunohistochemistry (IHC). It was observed in the western blot result that the band for P-EGFR was clearly displayed in the PBS-treated tumor tissues and weakened in the cetuximab-treated ones (Fig. 5f). When it came to IHC images, the PBS-treated group exhibited an extremely vivid yellow signal of P-EGFR while the cetuximab-treated group displayed a little yellow region (Fig. 5g). These observations suggested the low expression of P-EGFR and the low activity of the EGFR signaling pathways in the EGFR-inhibited group. In the electron transfer-triggered images, the afterglow signals of cetuximab-treated tumor tissues were apparently weaker than those of the PBS-treated group, revealing the inhibition of EGFR in the cetuximab-treated group (Fig. 5h and Supplementary Fig. 62). The statistical results of western blot and IHC demonstrated that the P-EGFR expression of the cetuximab-treated group was significantly lower than that of the PBS-treated group (Fig. 5i–j, Supplementary Figs. 63–64), matching well with the EGFR activity evaluated by the ETTE probe in tumor tissues (Fig. 5k). Consequently, the electron transfer-triggered images are highly consistent with the western blot and IHC results, verifying the feasibility of the ETTE probe for imaging the EGFR signaling activity in vivo.

Moreover, the biosafety of the ETTE nanoprobe was evaluated by measuring its tissue and blood compatibilities on female athymic BALB/c mice. Histological analysis of major organs, hematology analysis, and blood biochemical analysis were performed on healthy mice treated by intravenous injection with PBS or ETTE probe. The body weight of mice was monitored for 14 days after the injection, and no apparent body weight loss was observed (Supplementary Fig. 65). The histological analysis of major organs showed that the treatment of ETTE nanoprobe did not cause visible damage to all the tested organs after treatment (Supplementary Fig. 66), suggesting negligible organ toxicity of the probe. Furthermore, the general hematology parameters and standard blood biochemical indexes were assayed. Compared with the PBS-treated group, the ETTE probe-treated group displayed no statistically significant differences in both hematology parameters and blood biochemical indexes (Supplementary Figs. 67–68), demonstrating good blood compatibility and no obvious toxicity to liver and kidney. Altogether, the ETTE nanoprobe displays satisfiable biosafety in the mice model, laying a good foundation for further in vivo application.

### Mapping EGFR activity for efficacy assessment in vivo

During tumor treatment, the activity of EGFR relevant signal pathways keeps changing. When a tumor grows, tumor cells need to proliferate and differentiate rapidly, EGFR-related signaling pathways are activated in the cells. During treatment, the growth of tumor cells will be retarded, leading to the decrease of EGFR activity. To investigate whether electron transfer-triggered imaging can be used as a more general strategy to probe signaling events in vivo, it was further explored whether the EGFR activity evaluated by ETTE probe can reflect the therapeutic effect of EGFR non-targeted therapeutic agents.

**Fig. 6:**
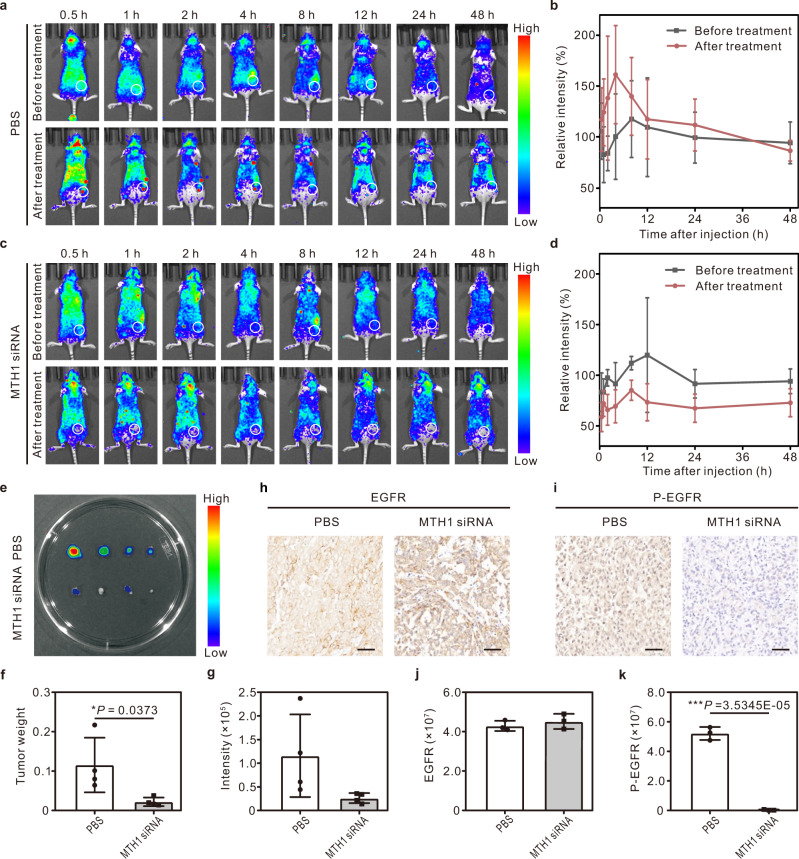
EGFR signaling pathway-related electron transfer-triggered imaging in vivo during tumor treatment. Time-dependent whole-body fluorescence images and corresponding quantitative data analysis of the tumor portion relative to the spinal symmetry region of A549 xenograft mice after intravenous injection of ETTE nanoprobe without (**a**, **b**) and with (**c**, **d**) treatment with MTH1 siRNA as a therapeutic agent for 14 days and before and after treatment. Color scale: 2.5 × 10^3^–1.5 × 10^4^. Data presented as mean values ± s.d., *n* = 3 mice. During the treatment, the afterglow images of mice treated with PBS or MTH1 siRNA were obtained at day 0 (Before treatment) and day 14 (After treatment). The afterglow imaging was performed after 1.0 s of white light excitation. The afterglow signal intensities are displayed as color maps. The white circles indicated the location of the tumor. The in vivo afterglow imaging was performed with triplicates. **e** A representative afterglow image of harvested tumor from the tumor-bearing mouse with ETTE nanoprobes, showing that the afterglow signal mostly appeared in large tumors. Color scale: 1.0 × 10^4^–5.0 × 10^4^. **f** The average tumor weight of each group at the experimental endpoint. Tumors were resected after the initial treatment. Data presented as mean values ± s.d., *n* = 4 mice. **g** Quantitative analysis of afterglow intensity in the tumor site. Data presented as mean values ± s.d., *n* = 4 mice. **h**, **i** IHC analysis of primary tumor sections. EGFR (**h**) and P-EGFR (**i**) in the tumor sections of each group were observed by IHC analysis. Scale bar: 50 μm. Quantitative analysis of EGFR (**j**) and P-EGFR (**k**) in tumor site observed by IHC analysis. Data are presented as the mean values ± s.d.; unpaired two-tailed Student’s *t*-test; *n* = 3 mice.

Using MTH1 siRNA as a non-targeted therapeutic agent^60^ (see Supplementary Information, section ‘siRNA Transfection’, and Supplementary Figs. 69–71), whole-body EGFR activity was evaluated by ETTE probe in mice, and the afterglow signal in the tumor area was quantitatively calculated (Supplementary Figs. 72 and 73). Considering that the metabolism in each mouse is unlikely the same, the relative afterglow signal intensity of the tumor portion relative to the spinal symmetry region was also calculated. In the untreated group, the afterglow signal after treatment in the tumor site was apparently higher than the initial signal (Fig. 6a, b), while the treated group showed a significant decay (Fig. 6c, d). After treatment, the correlation curve of afterglow signal versus injection time showed that the relative signal intensity fluctuates raised from 100 (Fig. 6b gray line) to 120% (Fig. 6b red line) for the untreated group, while it decreased from about 100 (Fig. 6d gray line) to 60% (Fig. 6d red line) for the treated group, suggesting that the EGFR activity in the tumor region was significantly weakened. Compared with the untreated group, the treated mice have statistically significant low tumor volume and tumor weight (Fig. 6e, f), the same as the trend of afterglow signal (Supplementary Figs. 74 and 6g), indicating that the efficacy of treatment can be evaluated by the ETTE probe. Finally, the expression of EGFR and P-EGFR in tumor tissues was characterized by IHC to show the activity of the relevant signaling pathways. In the untreated group, both EGFR and P-EGFR were highly expressed (Fig. 6h), while the treated group showed a high EGFR content and a low P-EGFR content (Fig. 6i), revealing that most EGFR molecules stay inactivated in the treated group. The statistical results further demonstrated that the EGFR activity of the treated group was significantly lower than that of the untreated group (Fig. 6j, k), in agreement with the ETTE images (Fig. 6e, g). These results are highly consistent with the widely accepted view that the EGFR signaling pathway in tumors stays activated to satisfy the needs of proliferation, angiogenesis, invasion, and metastasis during tumor growth^17,18^. Overall, this ETTE imaging method may offer valuable guidelines for the mapping of signaling activity in vivo and will inspire new strategies to evaluate therapeutic efficacy.

## Discussion

Our study suggests that an ETTE nanoprobe can achieve dynamic monitoring of EGFR signal pathway activity during tumor therapy. Compared with traditional IHC, which is the major source of our knowledge of signaling pathways, our probe has enabled the visualization of target signaling pathways and electron transfer in a temporally and spatially precise manner. Such a method provides the possibility to understand disease progression and molecular mechanisms, thus minimizing uncertainty caused by biopsy and sample processing. As an effective minimally invasive technique, this ETTE imaging method can monitor and characterize the activity of the target signal pathway in real-time and is expected to play a key factor in effective therapeutic decision-making and prognosis.

## Methods

### Reagents and materials

All reagents were used as received from commercial sources or prepared as described in references. All DNA synthesis reagents were purchased from Glen Research (Sterling, VA). Protected dFe phosphoramidites were synthesized in our lab.

Dulbecco’s phosphate-buffered saline (DPBS), Dulbecco’s modified Eagles medium (DMEM), Leibovitz’s L-15 medium (L-15), and fetal bovine serum (FBS) were obtained from Thermo Fisher Scientific Inc. All solutions used in the experiments were prepared using ultrapure water (resistance > 18 MΩ cm), which was obtained through a Millipore Milli-Q ultrapure water system (Billerica, MA, USA).

### Sample characterization

Oligonucleotides were prepared with an Applied Biosystems (ABI) 394 DNA/RNA synthesizer. ESI-MS spectra of oligonucleotides were performed by the Shanghai Sangon Mass Spectrometer (Shimadzu, Japan). Transmission electron microscopy (TEM) was carried out on an H-7000 NAR transmission electron microscope (Hitachi) with a working voltage of 100 kV. Atomic force microscopy (AFM) images of samples were obtained on a Multimode 8 (Bruker, USA). The confocal fluorescence imaging studies were performed on a confocal laser scanning microscope (FV1000, Olympus, Japan).

### Preparation of ZnGa_2_O_4_ nanoparticles

The ZnGa_2_O_4_ nanoparticles were synthesized in oil phase. First, 4.5 g of octadecanoic acid and 0.6 g of NaOH were dissolved in a mixed solvent containing 8 mL of deionized water and 18 mL of ethanol. Next, Zn(NO_3_)_2_ (2.0 M, 0.5 mL), Ga(NO_3_)_2_ (0.4 M, 5.0 mL) and Cr(NO_3_)_2_ (8 mM, 625 uL) were sequentially added to the above solution under vigorous stirring. Sodium hydroxide was added to adjust the pH to 10.0. The resulting mixture was left stirring for 20 min at room temperature. Then, the solution was transferred to a Teflon-lined autoclave and reacted at 220 °C for 10 h. The as-prepared ZnGa_2_O_4_ nanoparticles were collected by centrifugation and washed three times with cyclohexane: ethanol = 1:1.

### DNA synthesis

DNA oligonucleotide was synthesized using standard phosphoramidite chemistry on controlled pore glass supports on an ABI 394 DNA Synthesizer. The coupling times were 60 s. The completed sequences were then deprotected in saturated ammonium hydroxide at room temperature for 12 h and further purified by reversed-phase HPLC (Agilent 1260) on a C18 column using 0.1 M triethylamine acetate (TEAA) buffer (Glen Research) and acetonitrile (Sigma-Aldrich) as the eluents. The collected DNA products were dried and detritylated by dissolving and incubating DNA products in 200 µL of 80% acetic acid for 20 min. The detritylated DNA product was precipitated with NaCl (3 M, 25 µL) and ethanol (600 µL) and then desalted using Sep-Pac Plus C18 cartridges.

### Synthesis of Fe-base

Briefly, commercially available (S)-3-amino-1,2-propanediol was coupled with ferrocene carboxylic acid, and the corresponding phosphoramidite compound (Supplementary Fig. 11) was prepared for DNA solid-phase synthesis^61^.

### Fe-base characterization

Anhydrous solvent pyridine, CH_3_CN, CH_2_Cl_2_, and N,N-Dimethylformamide (DMF) were distilled under a nitrogen atmosphere and stored with 4 Å molecular sieves. Proton NMR spectra were recorded on a Bruker AM400 spectrometer. ^1^H-NMR, ^13^C-NMR, and ^31^P-NMR spectra were recorded on a Bruker AM400 spectrometer. Chemical shifts (*δ*) are reported in ppm, and coupling constants (J) are in hertz (Hz). The following abbreviations were used to explain the multiplicities: s singlet, d doublet, t triplet, q quartet, m multiplet, br broad.

Fourier transform infrared spectroscopy (FTIR) samples were prepared by mixing Fe-base with pre-dried KBr powder. FTIR spectra were recorded on an IR spectrometer instrument (Thermo, Nicolet 6700) in the 4000–400 cm^−1^ range.

The precise molecular weight of Fe-base was confirmed by a high-performance liquid chromatography-ion trap time-of-flight mass spectrometer (LCMS-IT-TOF, Shimadzu, Kyoto, Japan) equipped with an electrospray ionization (ESI) source, operating in positive ionization mode. The scanning ranges were m/z 50–1000. Shimadzu’s LCMS Solution software was used for data analysis.

### Preparation of ETTE nanoprobe

The ETTE nanoprobe was formed by a phase transfer method. The ETTE nanoprobe was attached to the nanoparticle by a hydrophilic-hydrophobic interaction. Briefly, 2 mg of ZnGa_2_O_4_ nanoparticles were dispersed in 2 mL of toluene by stirring. Next, 3 nmol of Fe-base-modified DNA oligonucleotide was dissolved in 1 mL of sterile water and then was added to the nanoparticle solution under stirring. The reaction mixture was kept at room temperature for 15 h under vigorous stirring. Afterward, the ZnGa_2_O_4_ was transferred into the lower water phase from the upper toluene phase. Then the water solution was transferred to a 2 mL of the centrifugal tube. The obtained ETTE nanoprobe was collected by centrifugation and washed with toluene three times, and further dispersed in 1 mL of sterile water.

### Preparation of working electrodes

To prepare the working electrodes, the glassy carbon electrodes (diameter 3 mm) were polished with 0.3 and 0.05 µm alumina powder and rinsed thoroughly with doubly distilled water, followed by washing ultrasonically in ethanol for 2 min. 2 mg persistent luminescence nanoparticles were dispersed in 1 mL ultrapure water to obtain a homogeneous suspension. 5 µL of the suspension was cast on the glassy carbon electrodes surfaces and allowed to dry at room temperature. Then, 3 µL of 625 µM GOD solution was dropped onto the surfaces of the ZnGa_2_O_4_-coated glassy carbon electrodes and dried at 4 °C. To maintain the stability of modified electrodes, 3 µL of 1% (v/v) Nafion solution was dropped onto GOD/ZnGa_2_O_4_/glassy carbon electrodes. The dried electrodes were subjected to electrochemical measurement.

For comparison, GOD/glassy carbon electrodes and glassy carbon electrodes were also prepared with the same procedures as those described above, but without ZnGa_2_O_4_ or GOD-coated ZnGa_2_O_4_. All modified electrodes were stored at 4 °C under dry conditions until further usage.

### Time-resolved fluorescence measurements

Samples were excited with a semiconductor laser (MPL-F-266) at 266 nm, and a filter was used to remove non-fluorescent scattering above 266 nm. Then, the optical path was adjusted to focus the fluorescence emitted by the sample on the PIN photodiode (Thorlabs APD120) to obtain an electrical signal corresponding to the lifetime of the fluorescence.

When a very short pulse (typically < 0.1 ms) excitation light irradiates a sample containing a fluorescent material, the fluorescent material will emit fluorescence with rapid exponential decay. When the fluorescence intensity becomes 1/e of the initial light intensity, the time t at this time is defined as the fluorescence lifetime^62^. The PIN photodiode captures the fluorescence of the material and then generates photoelectrons, which are converted into the output voltage *V*_*out*_. The relationship between the output voltage *V*_*out*_ of the Thorlabs APD120 photodiode and the incident optical power *P*_*out*_ is shown in Eq. (2), where *λ* is the persistent luminescence wavelength of the fluorescent material, and *R*(λ) is the sensitivity of the *λ* photodiode, where *R*(λ) is 25 A·W^−1^. The multiplier *M* and the transimpedance gain *G* are determined by the instrument and the ambient temperature. In this experiment, *M* = 50 and *G* is 10^5^ V·A^−1^. The formula for optical power is shown in Eq. (3)^63^, where *N* is the number of photons, h is Planck constant, c is the speed of light, *λ* is the persistent luminescence wavelength, and t is the detection time interval of the photodiode. As simplified, the relationship between the output voltage of the PIN photodiode and the photon number of the luminescent material is shown in formula (4).

2 $${V}_{{out}}={P}_{{out}}* R\left({{{{{\rm{\lambda }}}}}}\right){MG}$$

3 $${P}_{{out}}=\frac{{Nhc}}{{{{{{\rm{\lambda }}}}}}{{{{{\rm{t}}}}}}}$$

4 $$N=\frac{{P}_{{out}}{{{{{\rm{\lambda }}}}}}{{{{{\rm{t}}}}}}}{hc}=\frac{{V}_{{out}}{{{{{\rm{\lambda }}}}}}{{{{{\rm{t}}}}}}}{h{cMGR}({{{{{\rm{\lambda }}}}}})}$$

### Afterglow imaging of persistent luminescent nanoparticles

The persistent luminescent nanoparticles were placed in a 96-well plate. The nanorods were illuminated with a portable UV lamp for 2 min. After that, the UV lamp was removed, and the plate was immediately put into the IVIS Lumina XR Imaging System to record the decay images.

### Electrochemical characterization

Cyclic voltammetry (CV) measurements were carried out at room temperature on a CHI660D electrochemical working station (ChenHua Instruments Co. Ltd., Shanghai, China) using a standard three-electrode system. A modified GCE was the working electrode, and a saturated calomel electrode (SCE) and a platinum wire served as the reference and counter electrode, respectively. Before the measurement, the PBS solution (0.1 M, pH 7.2) containing various glucose concentrations (0.0, 0.5, 1.0, 2.0, and 5.0 mM) was purged with argon or oxygen for 10 min to prepare the argon- or oxygen-saturated solutions. All CV scans were recorded from −0.8 to 0.2 V with a scan rate of 50 mV·s^−1^, unless otherwise stated.

### Thermoluminescence measurements

In this experiment, an SL-08 thermoluminescence dosimeter was used to record the TL curves of the sample. The sample was placed on the sample plate, pre-irradiated with 15 W ultraviolet (254 nm) for 100 s, then heated from room temperature to 500 °C at a heating rate of 5 °C·s^−1^ and the TL curve of the sample was recorded.

Chen’s peak shape analysis method was used to analyze the thermoluminescence curve to obtain the trap depth of the sample. The trap depth by this analysis method is given by Eq. (5)^64–66^, where α corresponds to *τ* = (*T*_m_ − *T*_1_), *δ* = (*T*_2_ − *T*_m_), and *ω* = (*T*_2_ − *T*_1_). T_m_ is the maximum peak temperature, T_1_ and T_2_ are the temperatures corresponding to half the intensity on either side of the maximum peak, and *k* is the Boltzmann constant. Equations (6–8) give the C_α_, and the equation for b_α_ is shown in Eq. (9). Then, the average of *E*_*τ*_, *E*_*δ*_, *E*_*ω*_ is used to calculate the trap depth of the sample.

5 $${E}_{\alpha }={C}_{\alpha }\left(\frac{k{T}_{m}^{2}}{\alpha }\right)-{b}_{\alpha }(2k{T}_{m})$$

6 $${C}_{\tau }=1.51+3.0\left(\frac{{T}_{2}-{T}_{m}}{{T}_{2}-{T}_{1}}-0.42\right)$$

7 $${C}_{{{{{{\rm{\delta }}}}}}}=0.976+7.3\left(\frac{{T}_{2}-{T}_{m}}{{T}_{2}-{T}_{1}}-0.42\right)$$

8 $${C}_{{{{{{\rm{\omega }}}}}}}=2.52+10.2\left(\frac{{T}_{2}-{T}_{m}}{{T}_{2}-{T}_{1}}-0.42\right)$$

9 $${b}_{\tau }=1.58+4.2\left(\frac{{T}_{2}-{T}_{m}}{{T}_{2}-{T}_{1}}-0.42\right),{b}_{{{{{{\rm{\delta }}}}}}}=0,{b}_{{{{{{\rm{\omega }}}}}}}=1$$

### Electron paramagnetic resonance (EPR) measurements

The EPR signals of samples were acquired at 295 K using a JES-FA200 EPR spectrometer (JEOL, Tokyo, Japan) with an X-band standard frequency of 8.75–9.65 GHz. The following EPR parameters were used: frequency = 9.19 GHz, center field = 335 ± 10 mT, modulation frequency = 100 kHz, time constant = 0.03 s, and power = 0.998 mW.

The total 20 µL reaction mixture contained 8 µL glucose solution (0.0, 0.5 mM, 1.0 mM, 2.0 mM, and 5.0 mM dissolved in isopropanol), 4 µL DMPO (20 mg·mL^−1^, in isopropanol), 4 µL GOD (6.25 µM) and 4 µL isopropanol for detection of the DMPO/alkyl radical adducts.

### Electron tunneling probability calculation

The electrons transferred per unit time are proportional to the produced photons that determine the luminescence efficiency of the probe. Photoluminescence efficiency of the probe *y* is shown in Eq. (10).

10 $${{{{{\rm{y}}}}}}=\frac{{F}_{{PL}}}{{f}_{e}\left(1-R\right)F}=\frac{{R}_{r}}{\left(1-R\right)F}$$

where *F*_*PL*_ is the photoluminescence intensity of the probe, *f*_*e*_ is the correction factor, *R* is the reflection coefficient of the excitation light, *F* is the excitation intensity of external light (photons per second), *(1 − R) F* is the number of electrons excited from the probe per second, and *R*_*r*_ is the recombination rate of electron-holes^67^. According to the derivation of the equation, the luminescence efficiency of the probe is proportional to *R*_*r*_, the electron-hole recombination rate, under the same illumination intensity. Equation (11) is used to calculate *R*_*r*_.

11 $${R}_{r}=\frac{{np}}{{n}_{i}^{2}}32{{{{{{\rm{\pi }}}}}}}^{2}c{\left(\frac{{kT}}{{ch}}\right)}^{4}\int_{0}^{{{\infty }}}\frac{{N}^{3}{{\delta }}{u}^{3}{du}}{{e}^{u}-1}=\frac{{np}}{{n}_{i}^{2}}{R}_{i}$$

where *n* is the electron concentration of the probe, *p* is the hole concentration of the probe, *n*_*i*_ is the concentration of the electrons or holes in the intrinsic semiconductor, *k* is the Boltzmann constant, *c* is the speed of light, *h* is the Planck constant, *N* is the refractive index of the probe material, *Nδ* is the extinction coefficient, and *u* ≡ *hv*/*kT* represents the integration variable in Eq. (11)^68^. As simplified, the total recombination rate *R*_*i*_ per unit volume can be regarded as a temperature-dependent constant. In that way, at a certain temperature, the electron-hole recombination rate of the probe *R*_*r*_ is related only to the electron concentration in the probe *n*, the hole concentration *p*, and the concentration of electrons or holes in the intrinsic semiconductor *n*_*i*_.

### Cell culture

A549 cells: A549 cells derived from two different sources, American Type Culture Collection (ATCC) and China Center for Type Culture Collection (CCTCC). The A549 cells were maintained in DMEM high glucose (Gibco™, USA) with 10% fetal bovine serum (Zeta life, USA) and 1% penicillin/streptomycin (New Cell & Molecular Biotech, Co., Ltd).

HT-29 cells: HT-29 cells were derived from CCTCC. The HT-29 cells were maintained in DMEM high glucose (Gibco^TM^, USA) with 10% fetal bovine serum (Zeta life, USA) and 1% penicillin/streptomycin (New Cell & Molecular Biotech, Co., Ltd).

CAL-27 cells: CAL-27 cells were derived from CCTCC. The CAL-27 cells were maintained in DMEM high glucose (Gibco^TM^, USA) with 10% fetal bovine serum (Zeta life, USA) and 1% penicillin/streptomycin (New Cell & Molecular Biotech, Co., Ltd).

MDA-MB-231 cells: MDA-MB-231 cells were derived from CCTCC. The MDA-MB-231 cells were maintained in DMEM high glucose (Gibco^TM^, USA) with 10% fetal bovine serum (Zeta life, USA).

### Confocal fluorescence imaging

A549 cells were plated on 20 mm confocal laser dishes for 24 hours and divided into four groups for processing:

Group one: The cultured A549 cells were preincubated with ETTE nanoprobes (final concentration of 75 μg·mL^−1^) in a PBS environment at 37 °C for 40 min, then washed and detected in a PBS environment.

Group two: The cultured A549 cells were preincubated with ETTE nanoprobes (final concentration of 75 μg·mL^−1^) in a PBS environment at 37 °C for 30 min, then exposed to EGF (100 ng·mL^−1^) for 10 min to activate the EGFR signaling pathway.

Group three: The cultured A549 cells were preincubated with ETTE nanoprobes (final concentration of 75 μg·mL^−1^) in the medium supplemented with 10% serum at 37 °C for 40 min.

Group four: The cultured A549 cells were preincubated with ETTE nanoprobes (final concentration of 75 μg·mL^−1^) in the medium supplemented with 10% serum at 37 °C for 30 min, then exposed to EGF (100 ng·mL^−1^) for 10 min to activate the EGFR signaling pathway.

ETTE’s sensitivity detection:

Group one: The cultured A549 cells were preincubated with ETTE nanoprobes (final concentration of 75 μg·mL^−1^) in a PBS environment at 37 °C for 30 min. Then cells were incubated for 10 min with EGF at different concentrations (0.6 ng·mL^−1^, 4.0 ng·mL^−1^, 20.0 ng·mL^−1^, 100.0 ng·mL^−1^, 500.0 ng·mL^−1^) to activate the EGFR signaling pathway (*n* = 3).

Group two: The cultured A549 cells were preincubated with ETTE nanoprobes (final concentration of 75 μg·mL^−1^) in the medium supplemented with 10% serum at 37 °C for 30 min. Then cells were incubated for 10 min with EGF at different concentrations (0.6 ng·mL^−1^, 4.0 ng·mL^−1^, 20.0 ng·mL^−1^, 100.0 ng·mL^−1^, 500.0 ng·mL^−1^) to activate the EGFR signaling pathway (*n* = 3).

Then all the cells were washed with DPBS and fixed with paraformaldehyde. Subsequently, the cells were incubated with DAPI for 5 min for nuclear staining. After washing with DPBS three times, confocal fluorescence imaging studies were performed on the FV1000 confocal laser scanning microscope. The objective used for imaging was a UPlanSApo 60× oil immersion objective with a numerical aperture of 1.35 from Olympus (Japan). The lasers of 405 nm and 635 nm were used as the excitation light source for DAPI and ETTE probes. In addition, the cells were excited under an external 365 nm UV lamp for 2 min, and multiple images were taken continuously without UV excitation. All the experiments were performed in triplicate for reproducibility. The images were analyzed with ImageJ software. The ImageJ lookup table (LUT) named ‘Thermal’ was applied to obtain the pseudo-color images.

#### ETTE’s timescales evaluation

The A549 cells were plated on 20 mm confocal laser dishes for 24 h and divided into two groups for processing. The A549 cells were incubated with the ETTE probes (final concentration of 250 μg·mL^−1^) at 37 °C for 30 min. One group received no treatments and the other group was exposed to EGF (100 ng·mL^−1^) for 10 min. The two groups of cells were washed and tested in a PBS environment. The cells were excited for 2 min with an external UV lamp. Subsequently, the afterglow signal was continuously captured by laser confocal microscope at 1.0 s after UV excitation.

#### EGFR inhibition using afatinib

A549, CAL-27, and MDA-MB-231 cells were plated on a 20 mm confocal laser culture dish for 24 h. Different cell lines were separately incubated with ETTE probes and divided into three groups. The first group was treated with PBS; the second group was stimulated with EGF for 10 min; the third group was treated with afatinib (5 μM) for 3 h and then stimulated with EGF for 10 min. Then all the cells were washed with DPBS and fixed with paraformaldehyde. Subsequently, the cells were incubated with DAPI for 5 min for nuclear staining. After washing with DPBS three times, confocal fluorescence imaging studies were performed on the FV1000 confocal laser scanning microscope.

### Generating EGFR knockout A549 cell lines

The EGFR stable knockout (KO) A549 cells were established by using clustered regularly interspaced short palindromic repeats (CRISPR)/Cas9 system^69^. EGFR single guide RNAs (sgRNAs) were designed using the online CRISPR analysis tool (https://www.benchling.com/crispr/). The sgRNAs targeting the EGFR gene at exon 2 and Cas9 vectors (Supplementary Table 2) were cloned into 8138-EGFP-puro. For transfection, cells were seeded in a 96-well plate. Following transfection, cells were replated for single-cell cloning, propagated, and screened by a polymerase chain reaction (PCR) strategy designed to screen for EGFR knockout A549 cells. The EGFR primers for PCR were as follows, forward: 5′-CTACCACCCACCCCTTTAAATTTCA-3′, reverse: 5′-CATTAGCTGGTAAAATGGCTTTCTC-3′. The amplified PCR products were sequenced using the primers described above. EGFR knockout clones were further validated by Sanger sequencing and western blot.

### First-principles calculation

The first-principles calculation was carried out by the open-source QUANTUM ESPRESSO plane-wave density functional theory (DFT) package^42,43^. The Perdew-Burke-Ernzerhof (PBE) exchange-correlation functional was adopted and the plane-wave cut-off energies for the Fe-base and ZnGa_2_O_4_ were 626 and 598 eV, respectively. To avoid mirror interactions, vacuum spaces of 15 Å are added between adjacent cells in the nonperiodic direction. In the electronic ground-state computation of the Fe-base, only the Γ point of the Brillouin zone *k*-space was considered, whereas the *k*-space sampling was 5 × 5 × 5 for the ZnGa_2_O_4_. The crystal structures were fully relaxed until the force on each atom and total energy variations were <2.6 × 10^−2^ eV·Å^−1^ and 1.4 × 10^−3^ eV. Both materials are calculated separately and their band structures were assembled according to the work functions and vacuum energy.

### In vivo fluorescence imaging

Four-week-old female athymic BALB/c mice were purchased from the Hunan SJA Laboratory Animal Co., Ltd (Changsha, China) and used under protocols approved by the Institutional Animal Care and Use Committee of Hunan University (housing conditions, dark/light cycle: 12/12 h, temperature: 20 °C, humidity: about 40%). The mice were also cultured under specific pathogen-free (SPF) condition at SPF Animal Laboratory of School and Hospital of Stomatology, Wuhan University. This experiment was approved by the Experimental Animal Ethics Committee of School and Hospital of Stomatology, Wuhan University (Ethics No. S07918100F). Studies were conducted in accordance with the Tumor Policy for Mice, including adhering to the maximal tumor size of 1000 mm^3^. Tumor volumes in each group were then monitored, and mice were killed when tumor volumes reached 1000 mm^3^. To establish A549 tumor-bearing nude mouse model, female athymic BALB/c mice received a subcutaneous injection of 6 × 10^6^ A549 cells into the right backside. 4.5 nmol ETTE nanoprobe was injected intratumorally or intravenously via the tail vein, after mice were anesthetized with breathing oxygen and the anesthetic isopentane. Mouse tumors and other major organs were collected and measured for afterglow intensity.

### Biosafety evaluation

To verify the biosafety of ETTE probes, health BALB/c mice were randomly divided into two groups (*n* = 5) to be intravenously injected with 30 μL PBS and 30 μL ETTE probes, respectively. The mice’s body weights were monitored every other day. After 14 days, the blood was collected. Hematological and blood biochemical analyses were assessed using a blood cell analyzer (BC-2800vet, Mindray) and a biochemical analyzer (Chemray 240, Rayto). The major organs (liver, spleen, kidney, heart, and lung) were harvested and histologically analyzed by H&E staining.

### EGFR inhibition using cetuximab

Cetuximab was produced by MedChemExpress USA (#HY-P9905). Cetuximab was administered intraperitoneally at 1.5 µg per gram of body weight. Caliper measurements were used to calculate tumor volumes using the formula *V* = 1/2 (length × width × width).

### siRNA transfection

Small interfering RNAs (siRNAs) targeting MTH1 were synthesized by Sangon Biotech (China). The following sequences were used: siMTH1, 5′-GAC GAC AGC UAC UGG UUU CTT-3′ (sense), 5′-GAA ACC AGU AGC UGU CGU CTT-3′ (antisense). Cells were incubated with siRNA or materials at 37 °C for 48 hours. Further, siMTH1 were used on 0.8 × 10^5^ A549 cells. Cells were transfected with 10 nmol·L^−1^ of siRNAs using the Lipofectamine 2000 reagent at the concentrations indicated by the manufacturer (Invitrogen). Aptamer binding was verified 48 h post silencing. The transfection efficiency was tested by FAM-siRNA to ensure proper cell viability and delivery efficiency, which could be easily verified under the microscope.

### Western blot

Western blot was performed in accordance with our manual^61^. For EGFR expression in four kinds of cells, A549, CAL-27, HT-29, and MDA-MB-231 were separately seeded in a 6-well plate at a density of 1 × 10^5^ cells/well and cultured for 24 h. Subsequently, the cells were lysed by RIPA and total proteins were obtained. After high-speed centrifugation, the proteins were denatured. Forty micrograms of protein were loaded and separated by sodium dodecyl sulfate (SDS)-polyacrylamide gel electrophoresis (PAGE). The samples were transferred to polyvinylidene fluoride (PVDF) membranes (Millipore, Billerica, MA, USA) followed by immunoblotting with Anti-EGFR (#4267; 1:400, Cell Signaling Technology) after blocking. At last, Amersham ImageQuant 800 (GE Healthcare, USA) was used to visualize the protein bands after incubation with horseradish peroxidase (HRP)-conjugated anti-IgG antibody.

For P-EGFR expression in cells treated with EGFR inhibitors, A549, CAL-27, and MDA-MB-231 cells were seeded in 6-well plates at a density of 1 × 10^5^ cells/well, respectively. The cells were pretreated with gefitinib or afatinib at concentrations from 0 μM to 40 μM for 3 h before exposure to 100 ng·mL^−1^ EGF for 10 min at 37 °C. Subsequently, the cells were lysed by RIPA and total proteins were obtained. After high-speed centrifugation, the proteins were denatured. Forty micrograms of protein were loaded and separated by SDS-PAGE. The samples were transferred to PVDF membranes (Millipore, Billerica, MA, USA) followed by immunoblotting with anti-P-EGFR (#3777; 1:400, Cell Signaling Technology) after blocking. At last, Amersham ImageQuant 800 (GE Healthcare, USA) was used to visualize the protein bands after incubation with horseradish peroxidase (HRP)-conjugated anti-IgG antibody.

For MTH1 expression in A549 cells, A549 cells were lysed by RIPA and total proteins were obtained. After high-speed centrifugation, the proteins were denatured. Forty micrograms of protein were loaded and separated by SDS-PAGE. The samples were transferred to PVDF membrane (Millipore, Billerica, MA, USA) followed by immunoblotting with anti-MTH1 monoclonal antibody (#EPR15934-50; 1:800, Abcam, UK) after blocking. At last, Amersham ImageQuant 800 (GE Healthcare, USA) was used to visualize the protein bands after incubation with horseradish peroxidase (HRP)-conjugated anti-IgG antibody.

### Immunohistochemistry

The slides were subjected to deparaffination and rehydration. For epitope unmasking, the sections were boiled in 0.01 M citric acid buffer solution (pH 6.0) for 15 min at high pressure. Then, the sections were blocked for endogenous peroxidase activity by incubating sections in 3% hydrogen peroxide solution for 20 min at room temperature, and 10% goat serum was added to the sections to block non-specific binding. Next, the sections were incubated overnight at 4 °C in a humidified chamber with two primary antibodies, anti-EGFR (#4267; 1:400, Cell Signaling Technology) and anti-p-EGFR (#3777; 1:400, Cell Signaling Technology), or isotype-matched IgG controls. Subsequently, a secondary biotinylated IgG antibody solution and an avidin-biotin-peroxidase reagent were added to the slides (Sigma-Aldrich, USA). Peroxidase activity was detected by staining with DAB solution (Sigma-Aldrich). Then, the slides were counterstained with hematoxylin for 1–2 min. For immunofluorescence, the slides were incubated with fluorochrome-conjugated secondary antibody (Sigma-Aldrich) after incubation with primary antibody. Nuclear DNA was stained with DAPI (Sigma-Aldrich).

### Statistical analysis

Results are expressed as mean ± s.d. unless stated otherwise. Statistical analysis was performed with Prism 8.0 software (GraphPad Software) by an unpaired Student’s *t-*tests. *P-*value < 0.05 was considered statistically significant.

### Reporting summary

Further information on research design is available in the Nature Research Reporting Summary linked to this article.

## Supplementary information

Supplementary Information

Description of Additional Supplementary Files

Supplementary Movie 1

Reporting Summary

## Data Availability

The experimental data supporting the findings of this study are available within the article and Supplementary Information. The data for all graphs generated in this study are provided in the Source Data file. Source data are provided with this paper.

## Source data

Source Data
