# Supplementary material for: Electron transfer-triggered imaging of EGFR signaling activity
Source: Nat Commun. 2022 Feb 1;13:594. doi: 10.1038/s41467-022-28213-y (PMC8807759; doi:10.1038/s41467-022-28213-y)
Supplement: Supplementary file 1 — Supplementary Information [file 41467_2022_28213_MOESM1_ESM.pdf]

Supplementary Information for "Electron Transfer-Triggered Imaging of EGFR Signaling Activity"

Jie Tan[1], Hao Li[2], Cailing Ji[1], Lei Zhang[1], Chenxuan Zhao[3], Liming Tang [1], Caixin Zhang[1], Zhijun Sun[2,*], Weihong Tan[1,*], Quan Yuan[1,2,*]

## Supplementary Figures

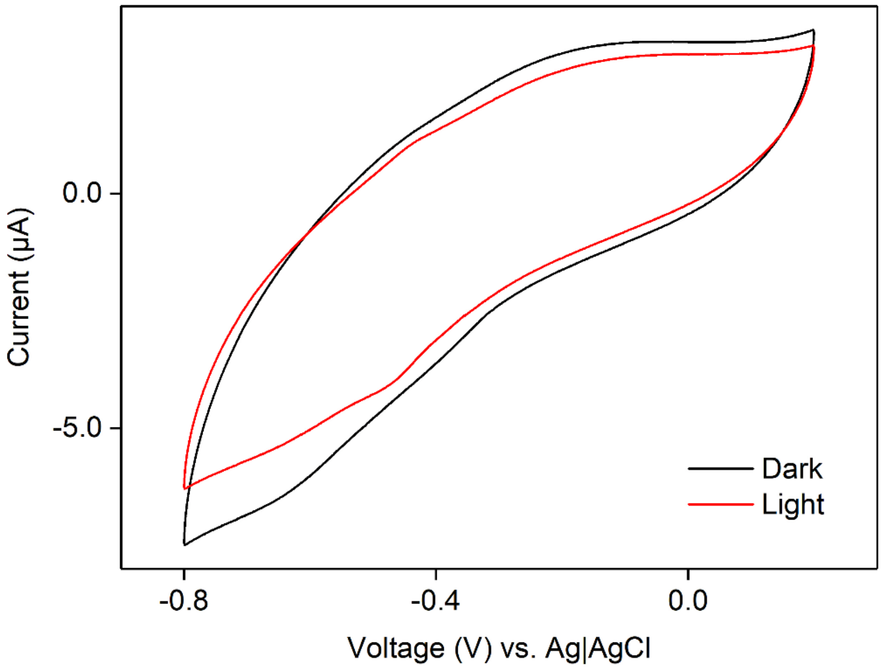


Supplementary Fig. 1. Cyclic voltammograms of the GOD modified electrode in argon-saturated PBS (pH 6.98) at a scan rate of 50 mV·s$^{-1}$ under dark (black line) and light (red line) condition.

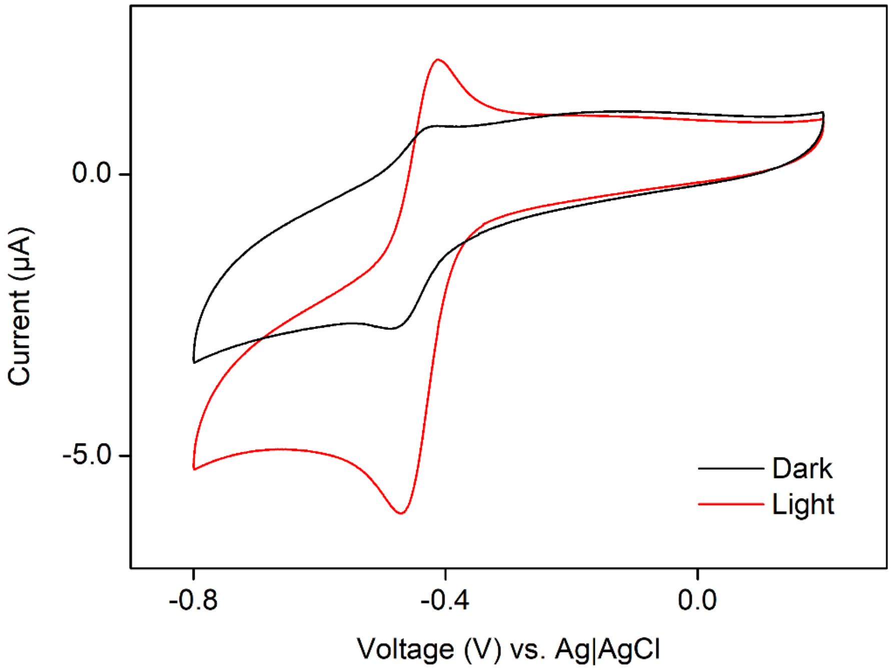


Supplementary Fig. 2. Cyclic voltammograms of the GOD/$ZnGa_2O_4$ modified electrode in argon-saturated phosphate buffer solution (pH 6.98) at a scan rate of 50 $mV\cdot s^{-1}$ under dark (black line) and light (red line) condition.

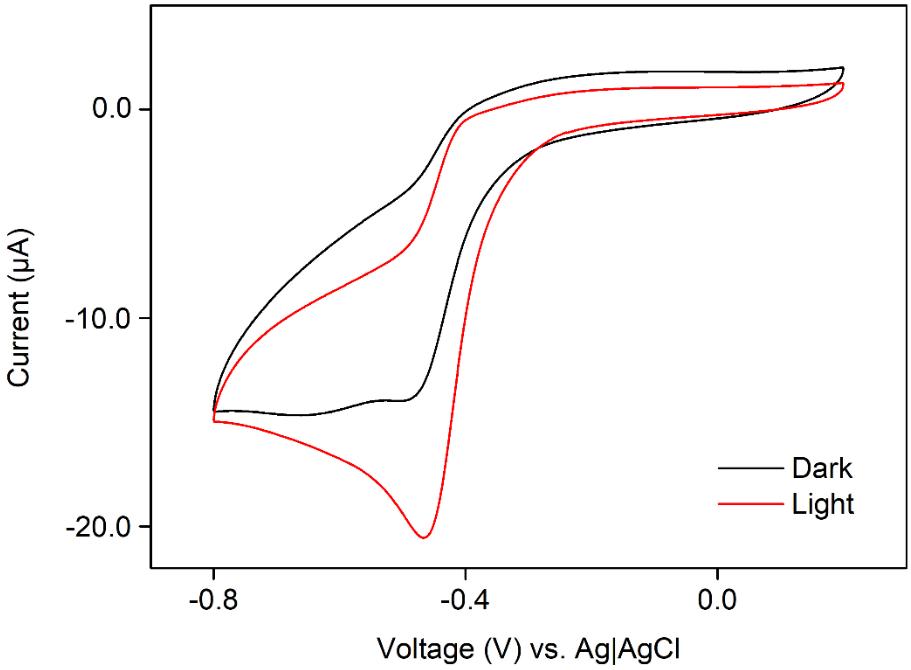


Supplementary Fig. 3. Cyclic voltammograms of the $ZnGa_2O_4$/GOD modified electrode in oxygen-saturated phosphate buffer solution (pH 6.98) at a scan rate of 50 $mV \cdot s^{-1}$ under dark (black line) and light (red line) condition.

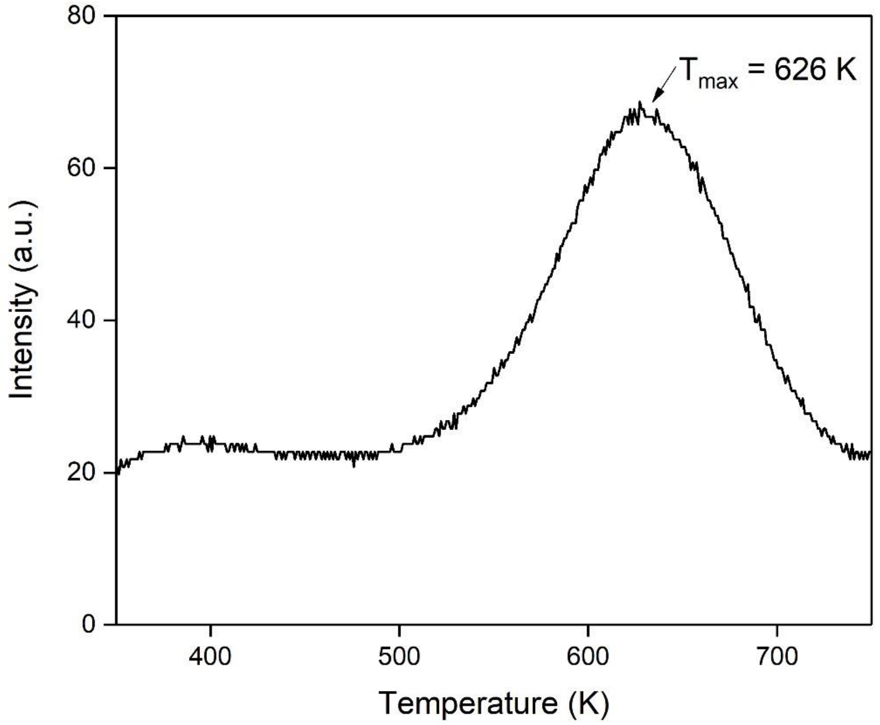


Supplementary Fig. 4. Thermoluminescence curve recorded with a 5°C·s$^{-1}$ heating rate of $ZnGa_2O_4$ after UV charging at room temperature. The thermoluminescence (TSL) excitation spectrum shows that the escape of charges from the local defect is more efficient at 626 K for excitation of the TSL/afterglow signal, indicating the existence of one active electron trap level centered at 989.6 meV in the forbidden band of $ZnGa_2O_4$.

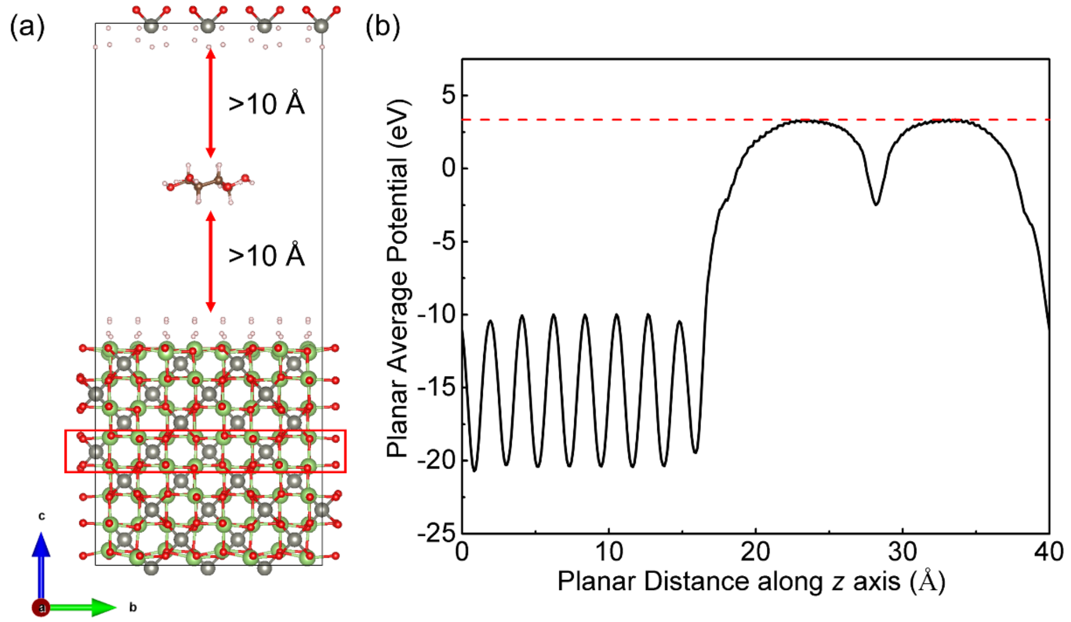


Supplementary Fig. 5. The computational model (a) and planar average potential (b) of a glucose molecule placed on a passivated 2 × 2 × 2 $ZnGa_2O_4$ slab. Red, pink, brown, green, gray balls represent O, H, C, Ga and Zn atoms, respectively.

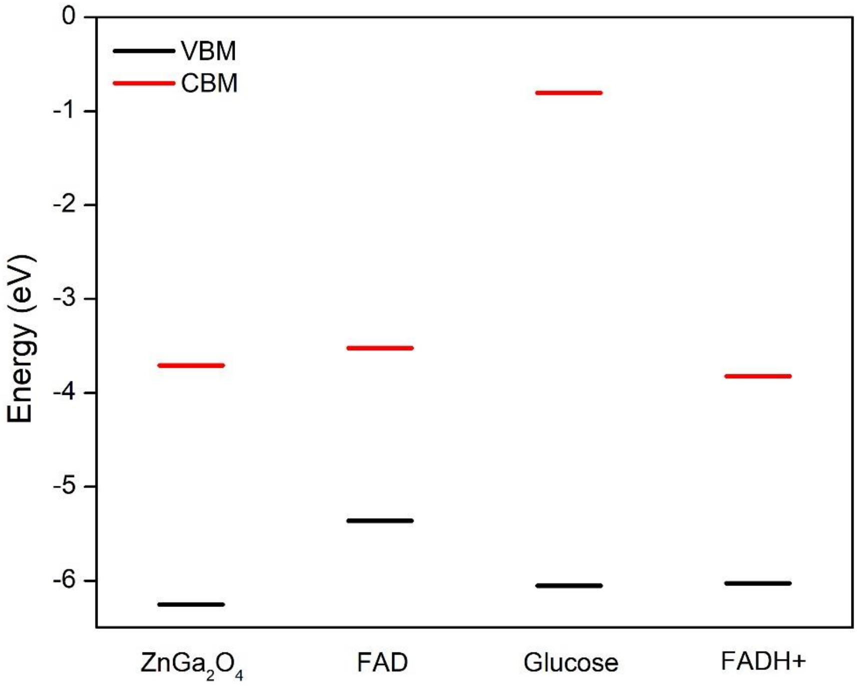


Supplementary Fig. 6. Energy band alignment diagrams for $ZnGa_2O_4$, FAD, glucose, and FADH+ as revealed.

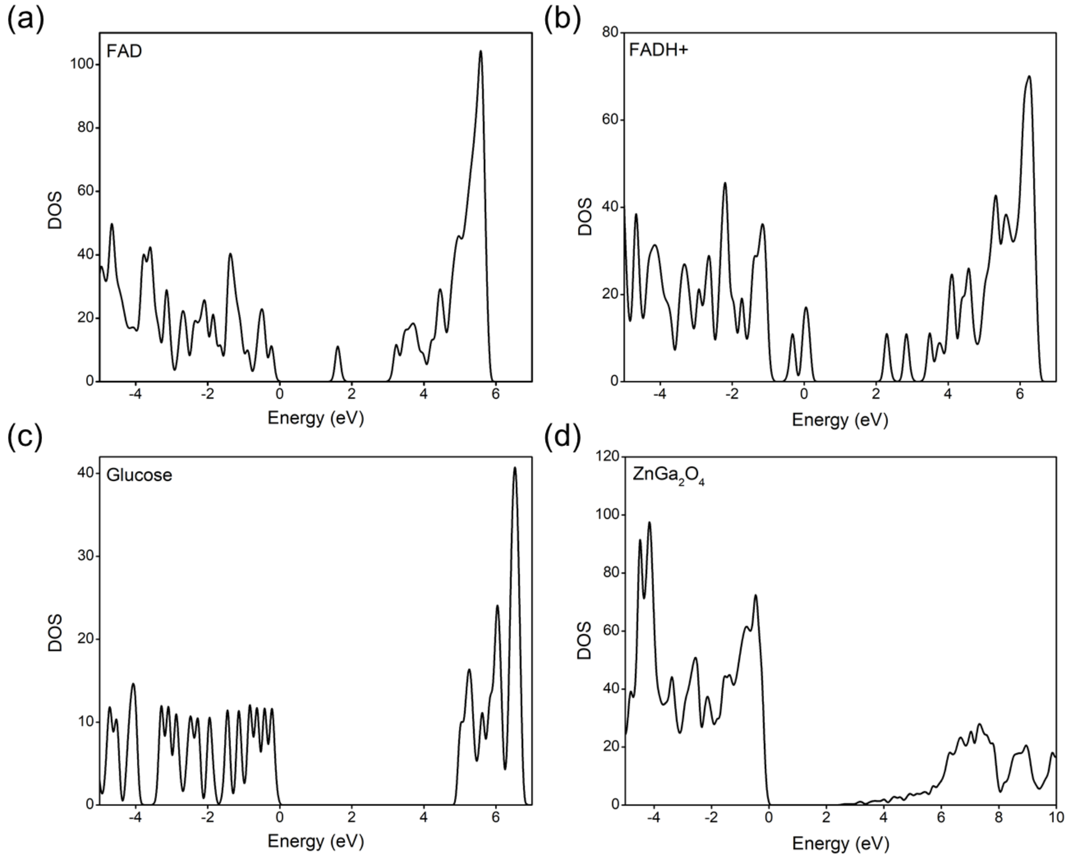


Supplementary Fig. 7. Calculated density of states (DOS) diagrams from first-principles simulations for FAD (a), FADH+ (b), glucose (c), and $ZnGa_2O_4$ unit cells (d).

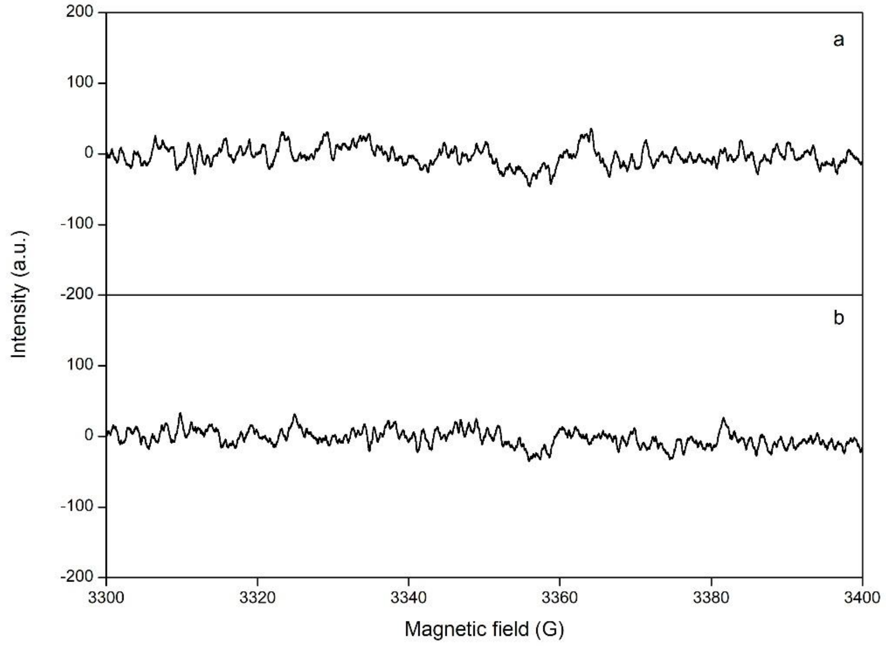


Supplementary Fig. 8. EPR spectrum of GOD without (a) and with (b) $ZnGa_2O_4$ observed at 295 K.

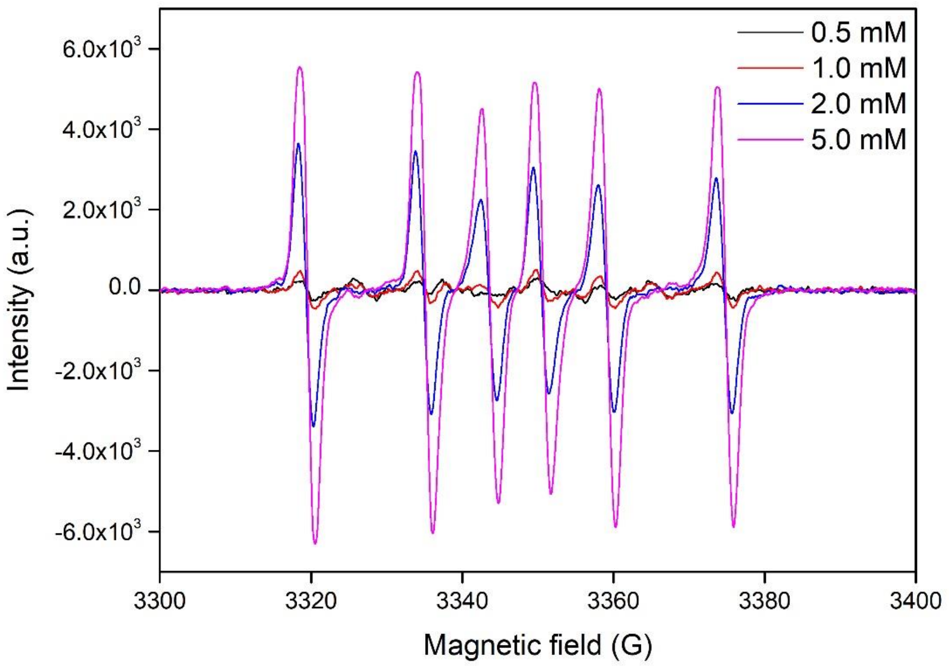


Supplementary Fig. 9. EPR spectrum of glucose at various concentrations (0.0 – 5.0 mM) and GOD mixtures observed at 295 K for the DMPO/alkyl radical adducts.

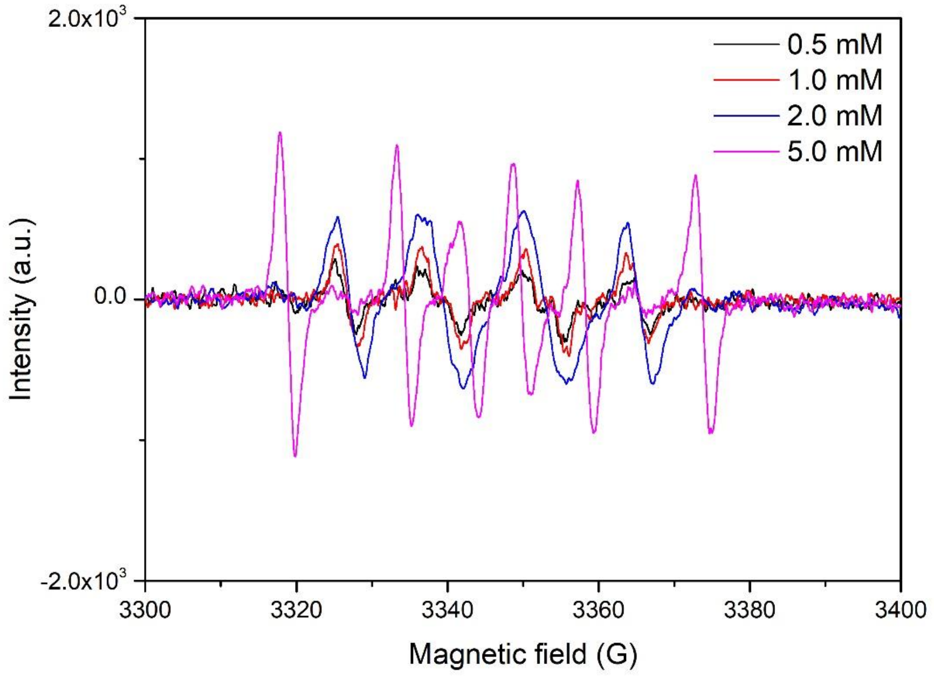


Supplementary Fig. 10. EPR spectrum of glucose at various concentrations (0.0 – 5.0 mM), GOD and $ZnGa_2O_4$ mixtures observed at 295 K for the DMPO/peroxyl radical adducts.

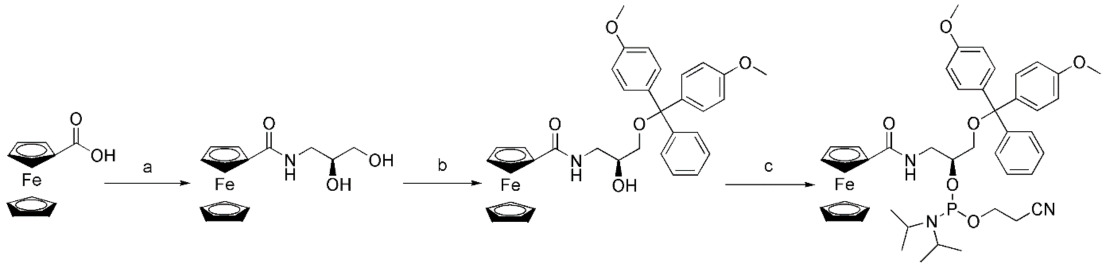


S1 S2 S3 S4

Supplementary Fig. 11. Synthesis of Fe-base. (a) (s)-3-Amino-1,2-propanediol, 2-(1H-Benzotriazole-1-yl)-1,1,3,3-tetramethyluronium hexafluorophosphate (HBTU), N, N-Diisopropylethylamine (DIPEA), and N,N-Dimethylformamide (DMF), (b) 4,4'-Dimethoxytrityl Chloride and Pyridine, and (c) Chlorophosphoramidite and dichloromethane (DCM).

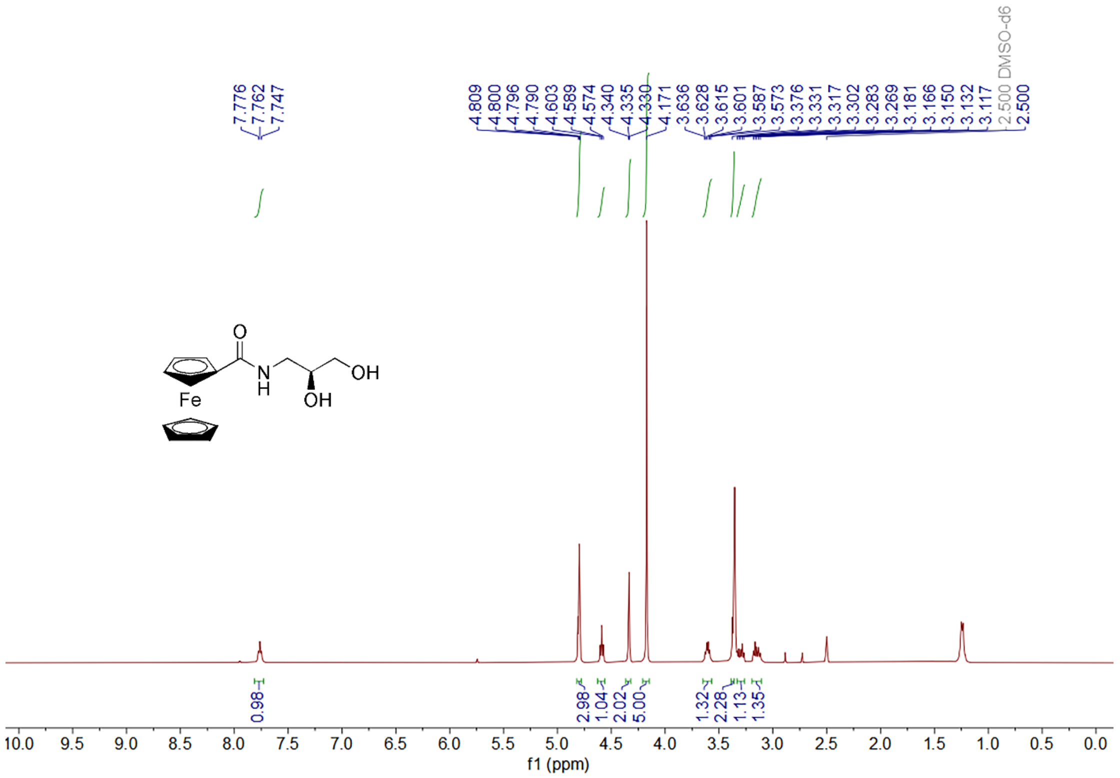

Supplementary Fig. 12. The $^1$H NMR spectrum of **compound S2**. **$^1$H NMR** (400 MHz, DMSO-$d_6$) δ 7.77 (t, $J$ = 5.9 Hz, 1H), 4.82 – 4.78 (m, 3H), 4.59 (t, $J$ = 5.9 Hz, 1H), 4.34 (t, $J$ = 1.9 Hz, 2H), 4.18 (s, 5H), 3.66 – 3.57 (m, 1H), 3.39 – 3.36 (m, 2H), 3.34 – 3.27 (m, 1H), 3.20 – 3.10 (m, 1H).

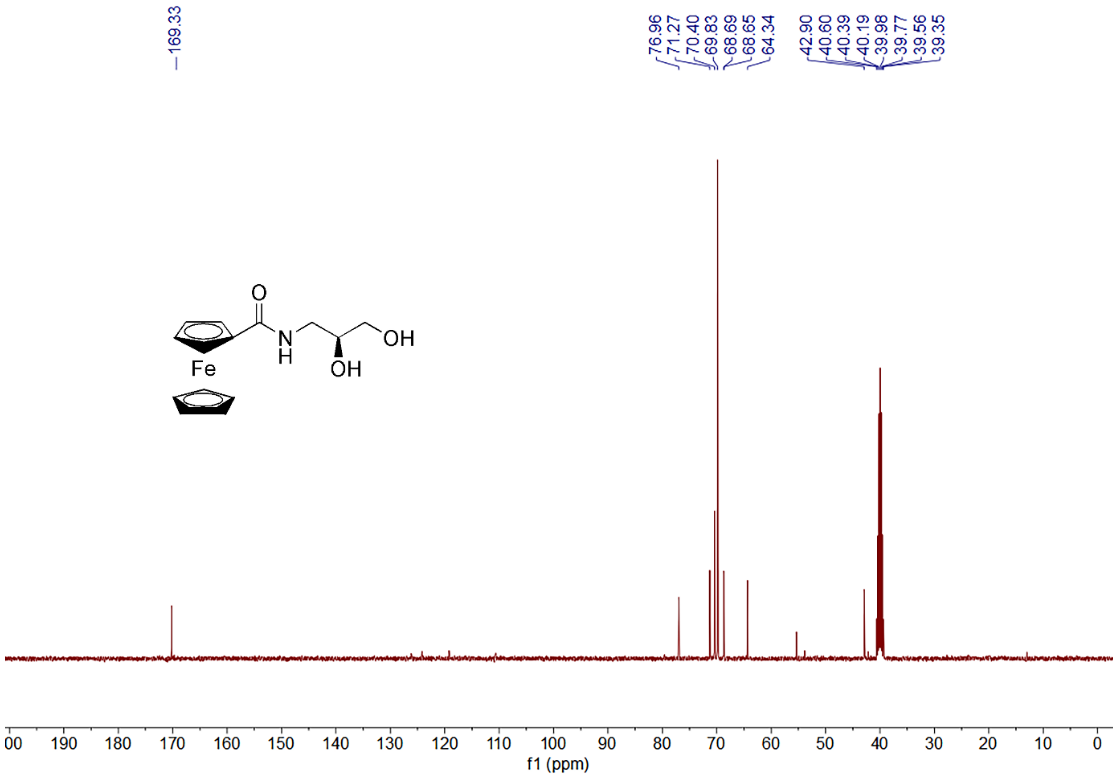


Supplementary Fig. 13. The $^{13}$C NMR spectrum of **compound S2**. **$^{13}$C NMR** (101 MHz, DMSO-$d_6$) δ 169.3, 77.0, 71.3, 70.4, 69.8, 68.7, 68.7, 64.3, 42.9.

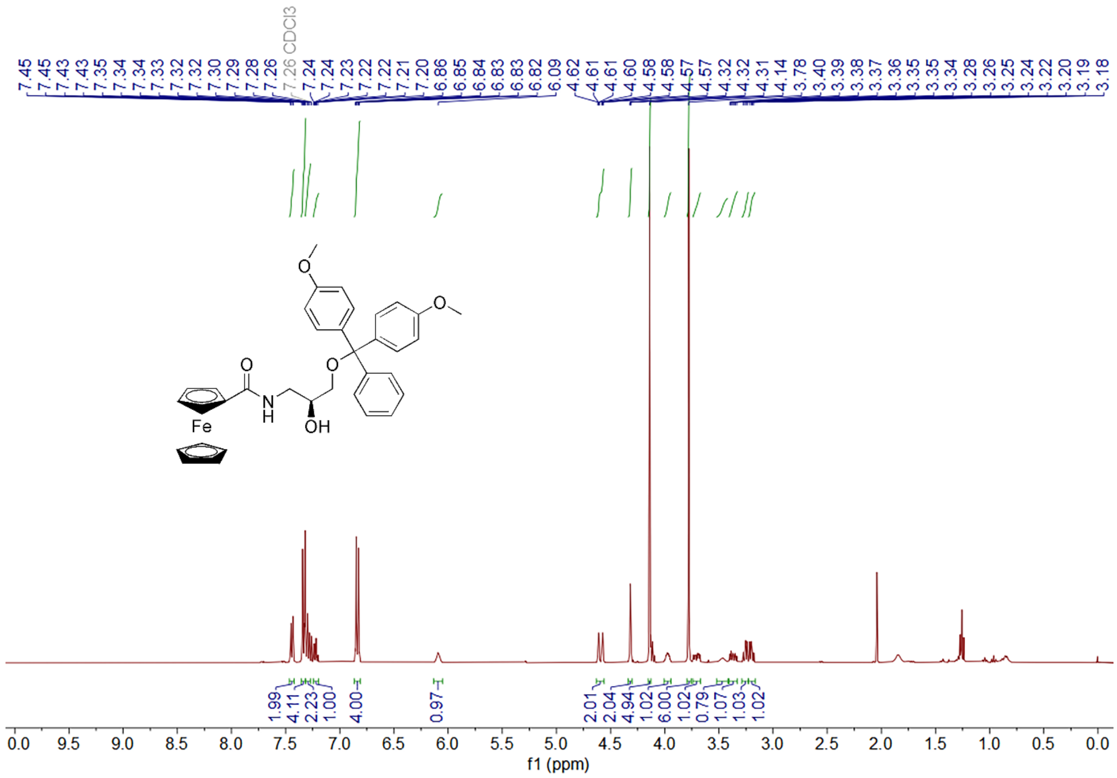


Supplementary Fig. 14. The $^1$H NMR spectrum of **compound S3**. **$^1$H NMR** (400 MHz, $CDCl_3$) δ 7.46 – 7.42 (m, 2H), 7.36 – 7.32 (m, 4H), 7.32 – 7.27 (m, 2H), 7.24 – 7.19 (m, 1H), 6.92 – 6.71 (m, 4H), 6.09 (s, 1H), 4.63 – 4.56 (m, 2H), 4.32 (t, *J* = 1.9 Hz, 2H), 4.14 (s, 5H), 4.01 – 3.94 (m, 1H), 3.78 (s, 6H), 3.74 – 3.67 (m, 1H), 3.47 (s, 1H), 3.41 – 3.33 (m, 1H), 3.28 – 3.23 (m, 1H), 3.22 – 3.16 (m, 1H).

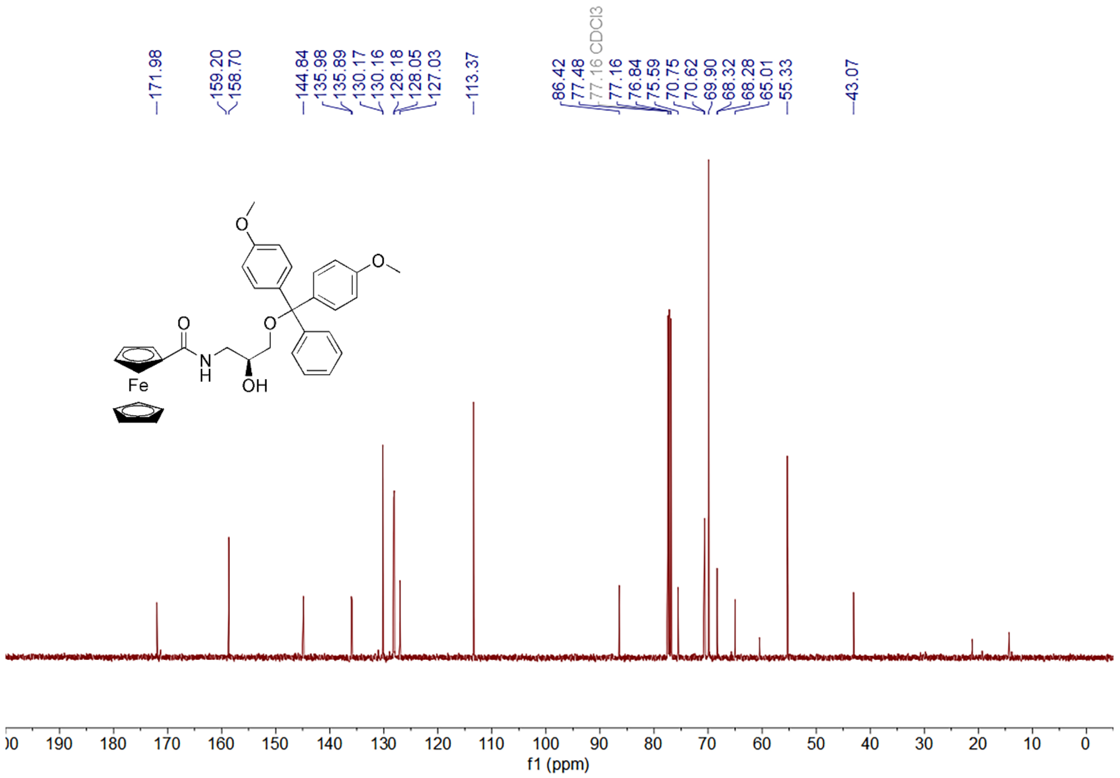


Supplementary Fig. 15. The $^{13}$C NMR spectrum of **compound S3**. **$^{13}$C NMR** (101 MHz, $CDCl_3$) δ 172.0, 159.2, 158.7, 144.8, 136.0, 135.9, 130.2, 130.2, 128.2, 128.1, 127.0, 113.4, 86.4, 75.6, 70.8, 70.6, 69.9, 68.3, 68.3, 65.0, 55.3, 43.1.

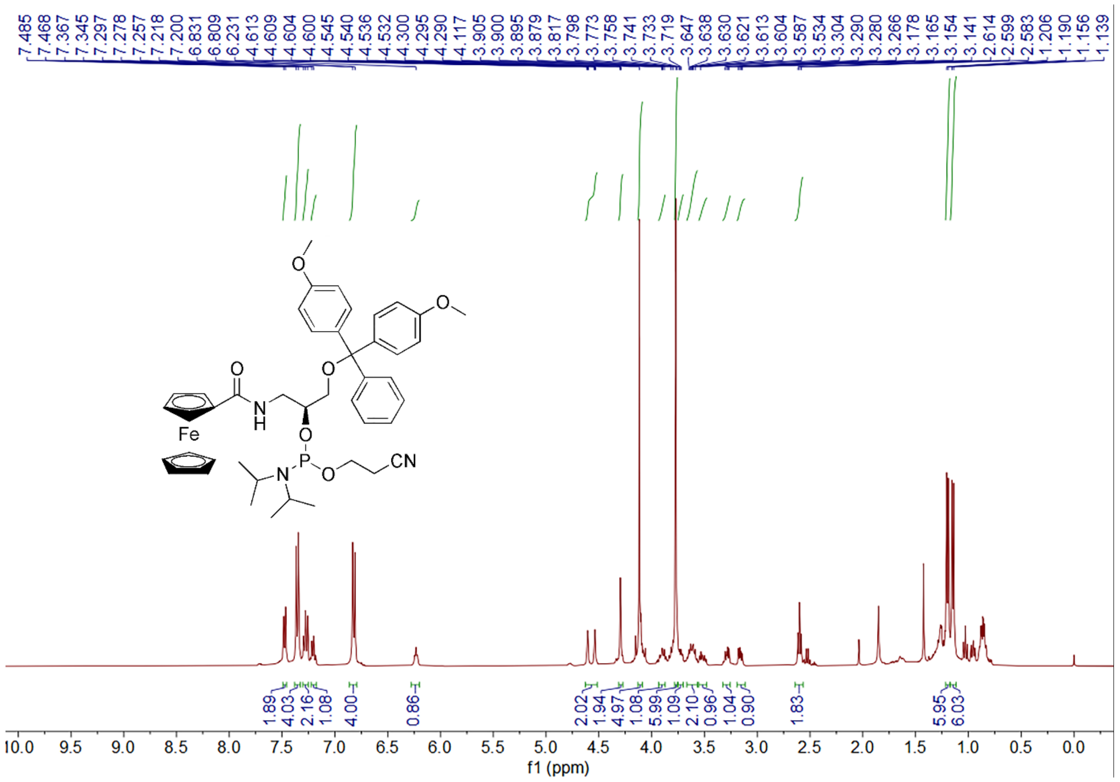

Supplementary Fig. 16. The $^{1}$H NMR spectrum of **compound S4**. **$^{1}$H NMR** (400 MHz, $CDCl_3$) δ 7.48 (d, *J* = 7.0 Hz, 2H), 7.36 (d, *J* = 8.8 Hz, 4H), 7.28 (t, *J* = 8.0 Hz, 2H), 7.20 (t, *J* = 7.3 Hz, 1H), 6.82 (d, *J* = 8.9 Hz, 4H), 6.23 (t, *J* = 5.5 Hz, 1H), 4.62 – 4.51 (m, 2H), 4.30 (t, *J* = 2.0 Hz, 2H), 4.12 (s, 5H), 3.93 – 3.87 (m, 1H), 3.77 (s, 6H), 3.75 – 3.70 (m, 1H), 3.67 – 3.57 (m, 1H), 3.56 – 3.48 (m, 1H), 3.33 – 3.26 (m, 1H), 3.19 – 3.11 (m, 1H), 2.60 (t, *J* = 6.3 Hz, 2H), 1.20 (d, *J* = 6.7 Hz, 6H), 1.15 (d, *J* = 6.7 Hz, 6H).

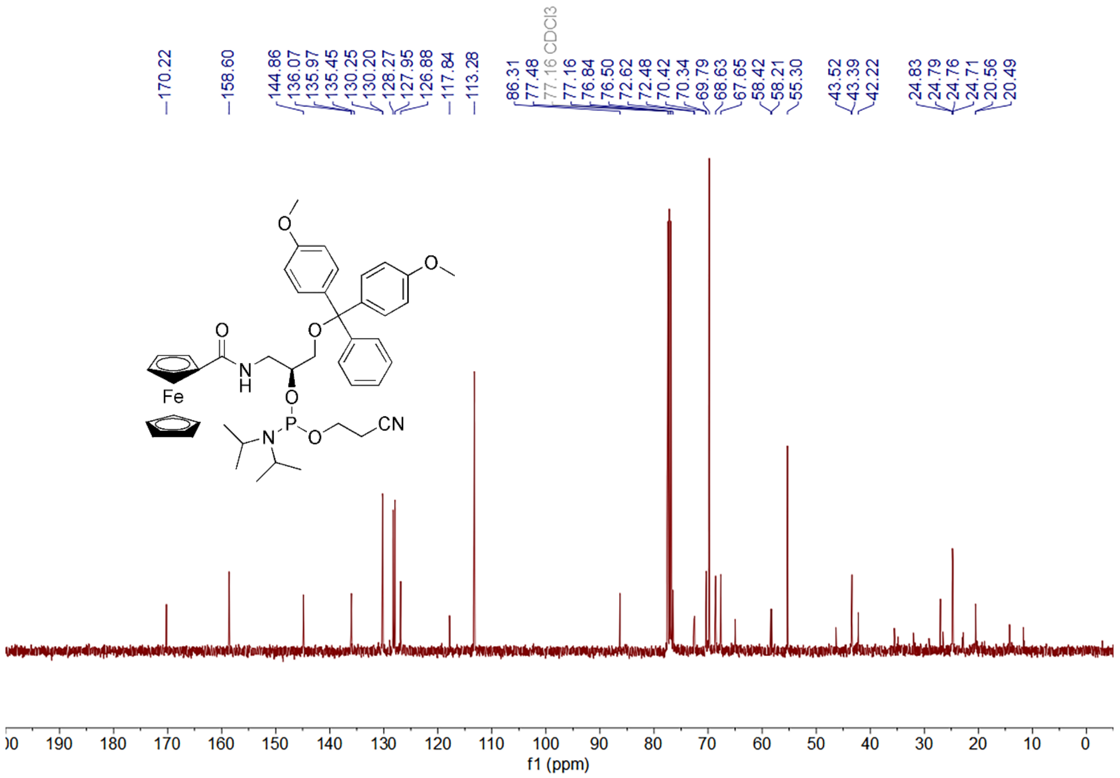


Supplementary Fig. 17. The $^{13}$C NMR spectrum of **compound S4**. **$^{13}$C NMR** (101 MHz, $CDCl_3$) δ 170.2, 158.6, 144.9, 136.1, 136.0, 130.3, 130.2, 128.3, 128.0, 126.9, 117.8, 113.3, 86.3, 76.5, 72.6 (d, *J* = 14.6 Hz), 70.4, 70.3, 69.8, 68.1 (d, *J* = 98.2 Hz), 58.3 (d, *J* = 20.8 Hz), 55.3, 43.5 (d, *J* = 12.8 Hz), 42.2, 24.8 (d, *J* = 4.2 Hz), 24.7 (d, *J* = 5.2 Hz), 20.5 (d, *J* = 6.9 Hz).

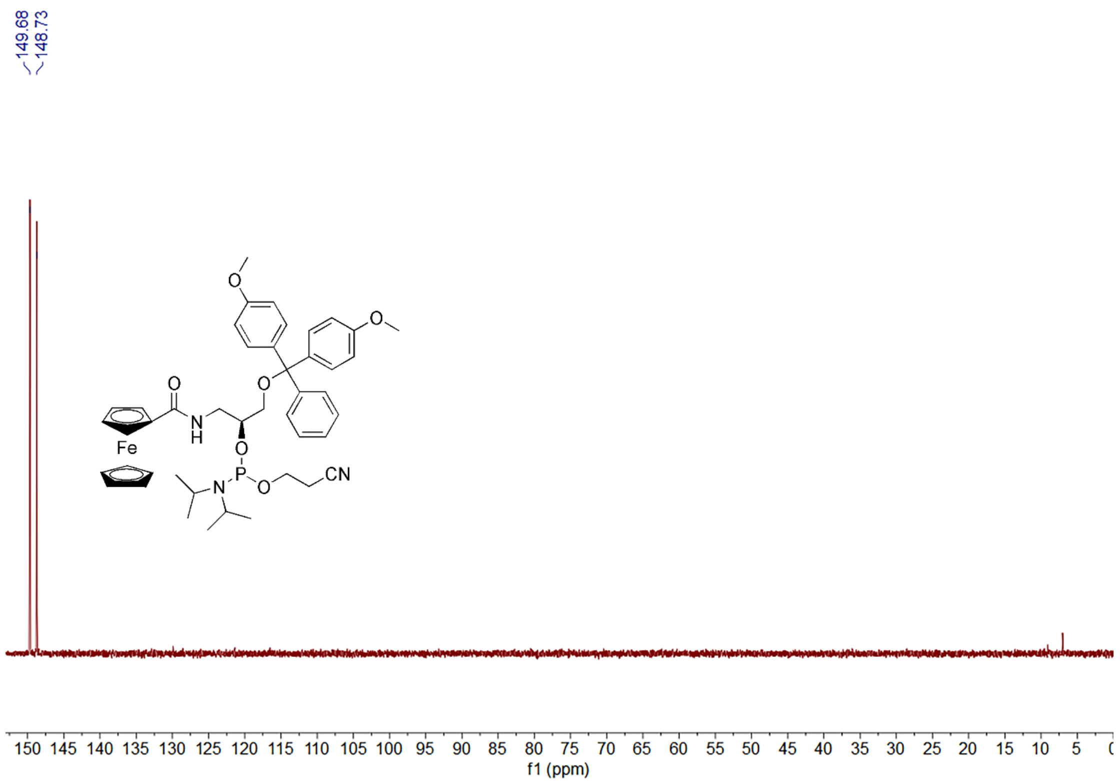


Supplementary Fig. 18. The $^{31}$P NMR spectrum of **compound S4**. **$^{31}$P NMR** (162 MHz, $CDCl_3$) δ 149.68, 148.73.

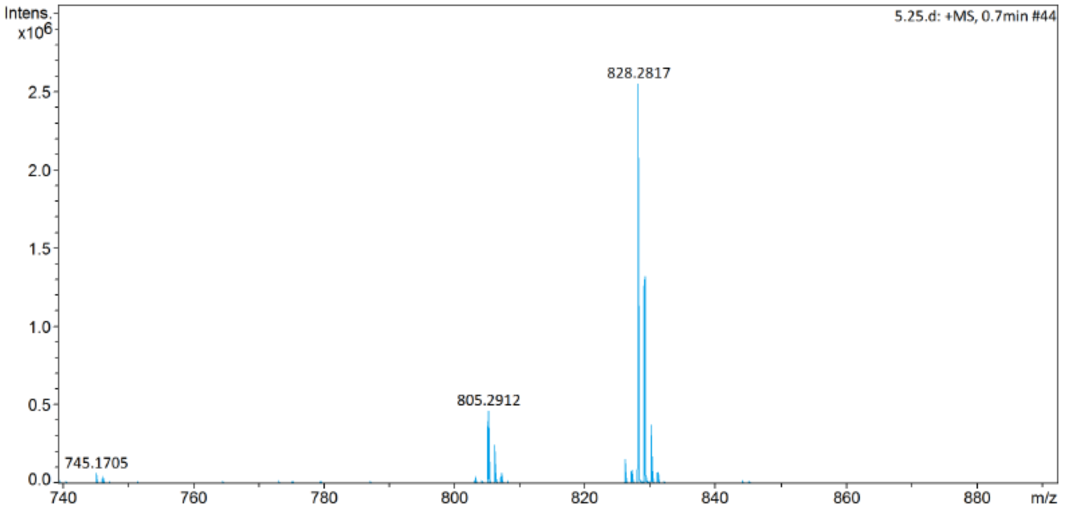


Supplementary Fig. 19. HR-MS spectrum of Fe-base; calculated mass: [M] = 805.28, [M+Na] = 828.30.

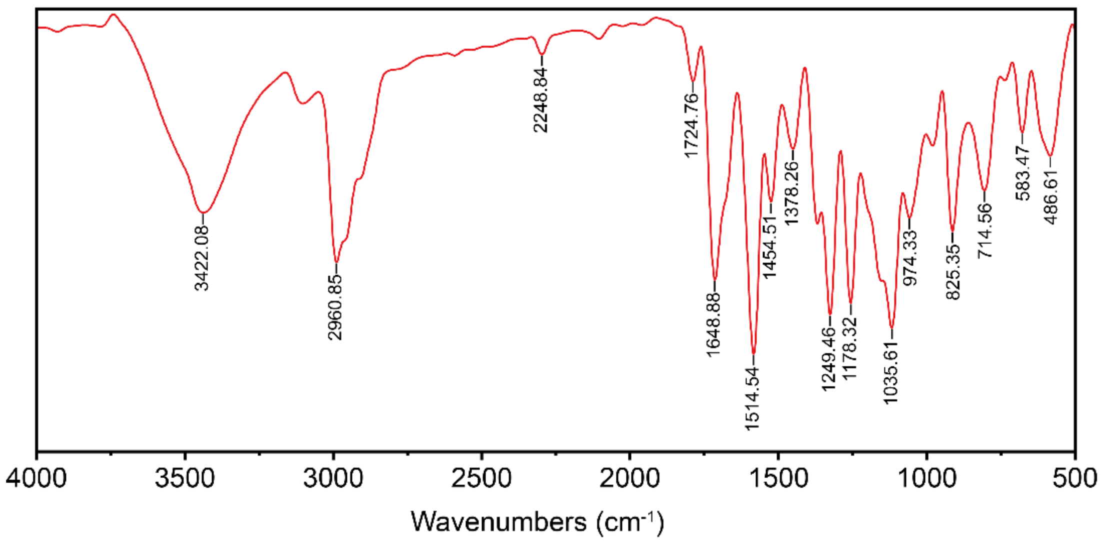


Supplementary Fig. 20. Fourier transform infrared spectrum of Fe-base.

The broad peak centered at 3422 $cm^{-1}$ is attributed to the N-H stretching vibration. The peak at around 2960 $cm^{-1}$ comes from the C-H stretching vibration of methyl/benzene ring. The adsorption bands at 2248, 1724, 1454 and 1378 $cm^{-1}$ are assigned to C≡N stretching, C=O stretching, methylene C-H bending and methyl C-H bending, respectively. The peaks at 1648 and 1514 $cm^{-1}$ indicate the presence of amide, and numerous peaks between 1250 and 1035 $cm^{-1}$ attest to the presence of some C-O-C moieties. The peaks at 1035, 974 and 825 $cm^{-1}$ arising from the out-of-plane vibration of cyclopentadiene and the 486 $cm^{-1}$ peak due to the unique asymmetric ring metal stretching vibration of ferrocene molecules, proving the presence of ferrocene in the sample[1, 2]. The result of FT-IR confirmed that the Fe-base was synthesized successfully.

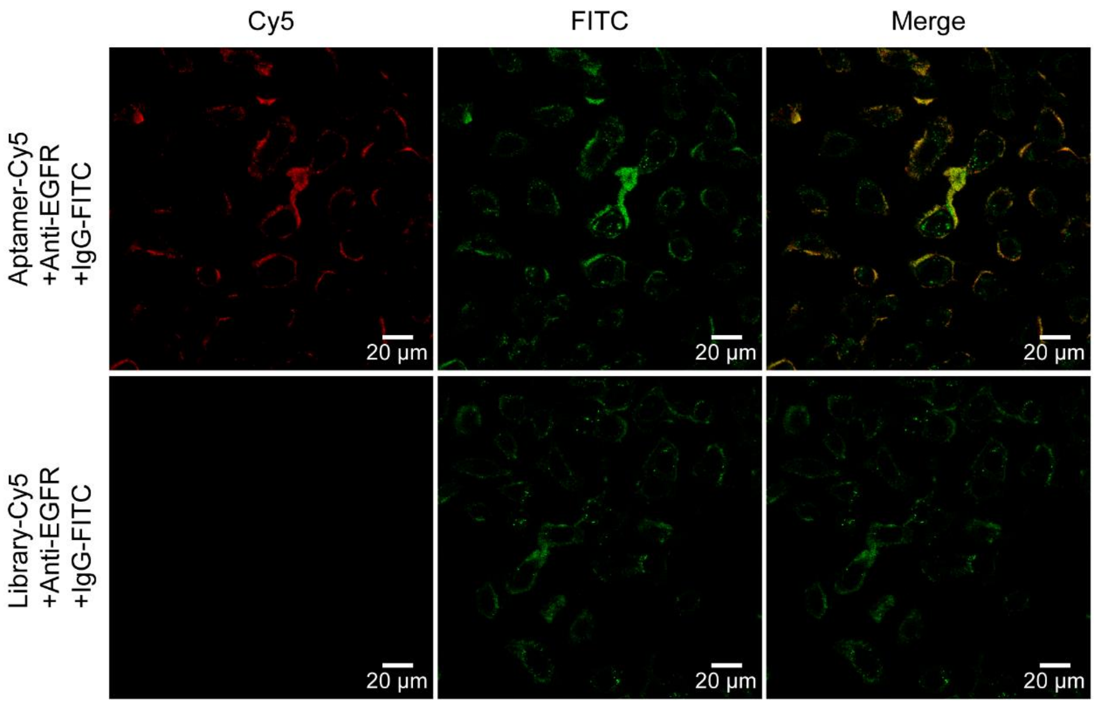


Supplementary Fig. 21. Fluorescence confocal images of A549 cells incubated with Cyanine5-labeled aptamer (Aptamer-Cy5), Cyanine5-labeled random nucleic acid library (Library-Cy5), anti-EGFR and fluorescein isothiocyanate-labeled IgG (IgG-FITC). Column 1 is the Cy5 channel, column 2 is the FITC channel, and column 3 is the merge of Cy5 channel and FITC channel. Cells in row 1 were incubated with Aptamer-Cy5, anti-EGFR and IgG-FITC. Cells in row 2 were incubated with Library-Cy5, anti-EGFR and IgG-FITC. Scale bar, 20 μm. The imaging experiments were repeated independently three times and similar results were obtained.

As shown by confocal microscopy images, red signal from aptamer and green signal from anti-EGFR were clearly present and merged in yellow, indicating the co-localization of aptamer and anti-EGFR. However, for library-treated cells, such co-localization was not observed. These results confirm that EGFR is the target of the aptamer.

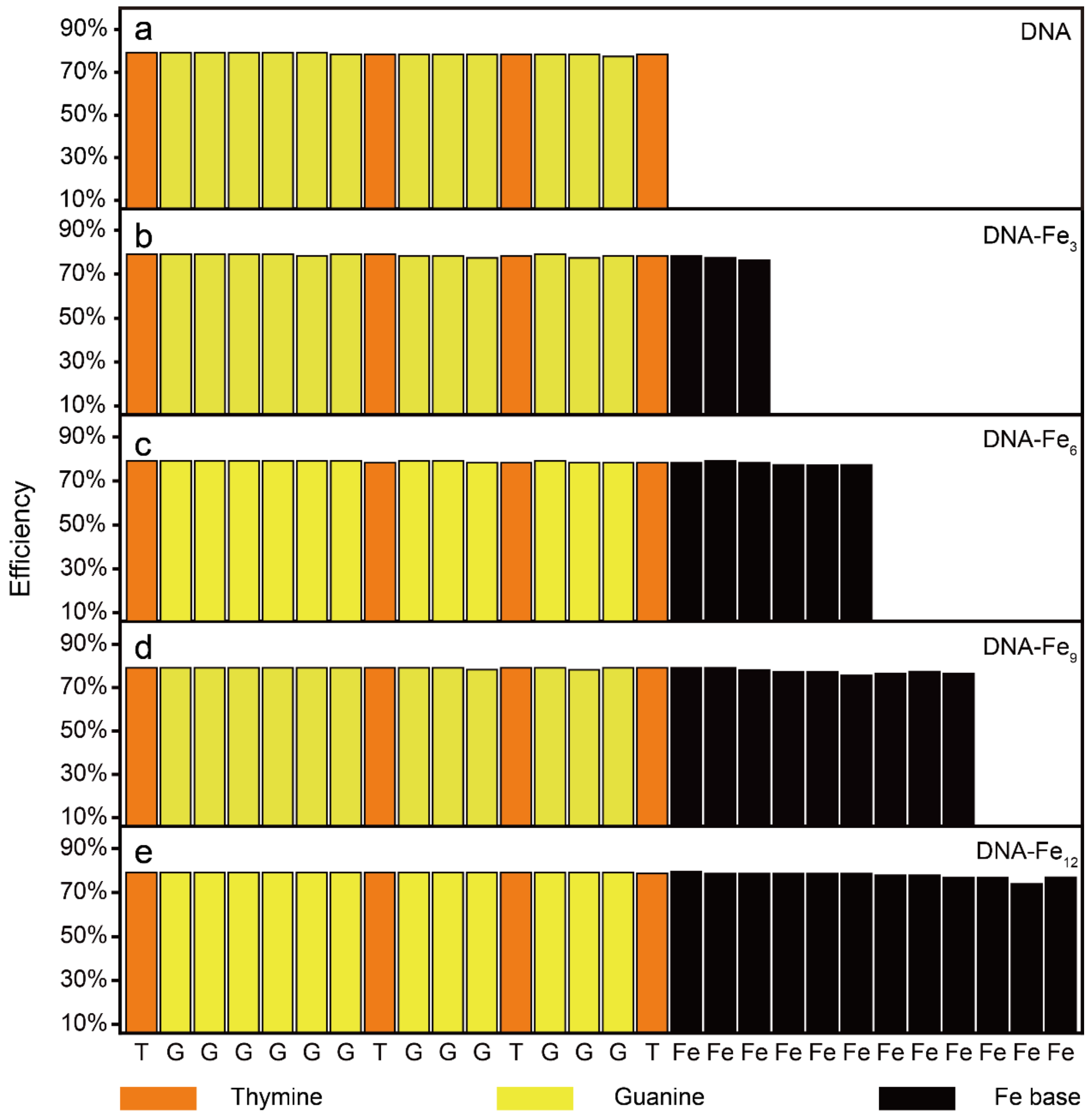


Supplementary Fig. 22. Coupling efficiencies of DNA phosphoramidites. Stepwise coupling yield of pure DNA (a), DNA-$Fe_3$ (b), DNA-$Fe_6$ (c), DNA-$Fe_9$ (d), DNA-$Fe_{12}$ (e), respectively. The orange column and the yellow column represent the coupling efficiency of standard T and G nucleotides respectively, and the black column represents the coupling efficiency of the ferrocene moiety.

In the DNA synthesizer, the coupling efficiency of each step could be evaluated by the amount of released dimethoxytrityl (DMT) group. The coupling efficiency of the Fe-containing nucleoside phosphoramidites was found to be similar to that obtained with commercial phosphoramidites of the standard nucleosides.

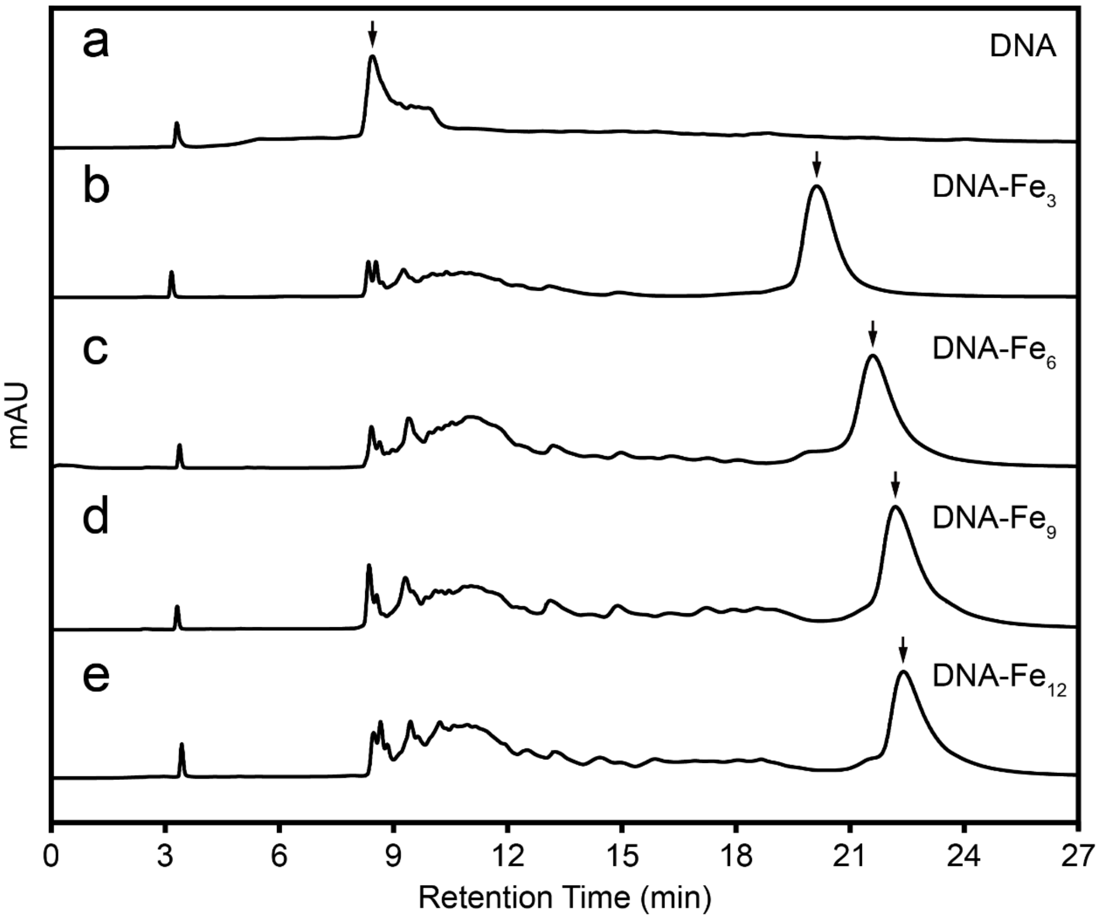


Supplementary Fig. 23. HPLC chromatogram analysis of pure DNA (a), DNA-$Fe_3$ (b), DNA-$Fe_6$ (c), DNA-$Fe_9$ (d) and DNA-$Fe_{12}$ (e) prepared by the DNA synthesizer. HPLC conditions: C18 column at 25°C and a flow rate of 1 $mL \cdot min^{-1}$, absorbance wavelength 260 nm; Mobile phase: Solvent A was 0.1 $mol \cdot L^{-1}$ acetic acid/triethylamine (100:229.2, v: v) and Solvent B was acetonitrile.

The obtained oligonucleotides were further purified by reversed-phase high-performance liquid chromatography (HPLC) on a C18 column using 0.1 M triethylamine acetate (TEAA) buffer and acetonitrile as the eluents. Fe-containing oligonucleotides eluted later than pure DNA, because of the hydrophobic interaction of ferrocene moiety in the mobile phase with the reverse-phase column containing C18 alkyl chain.

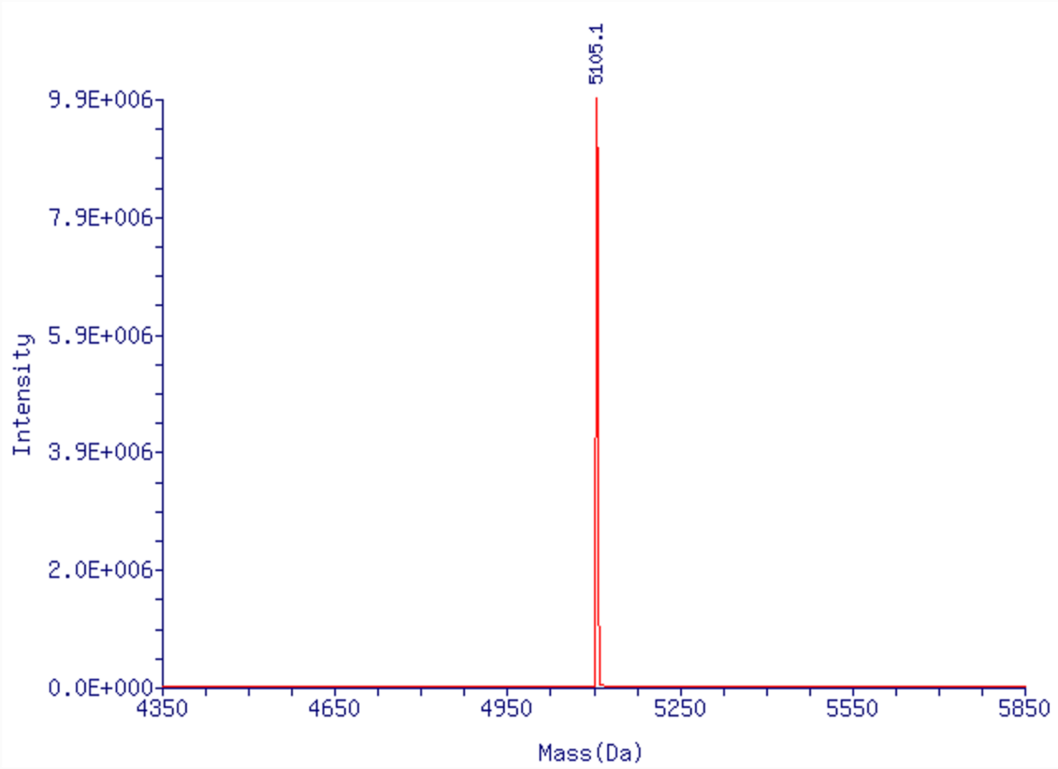


Supplementary Fig. 24. ESI-MS spectrum of pure DNA; calculated mass: 5105.34.

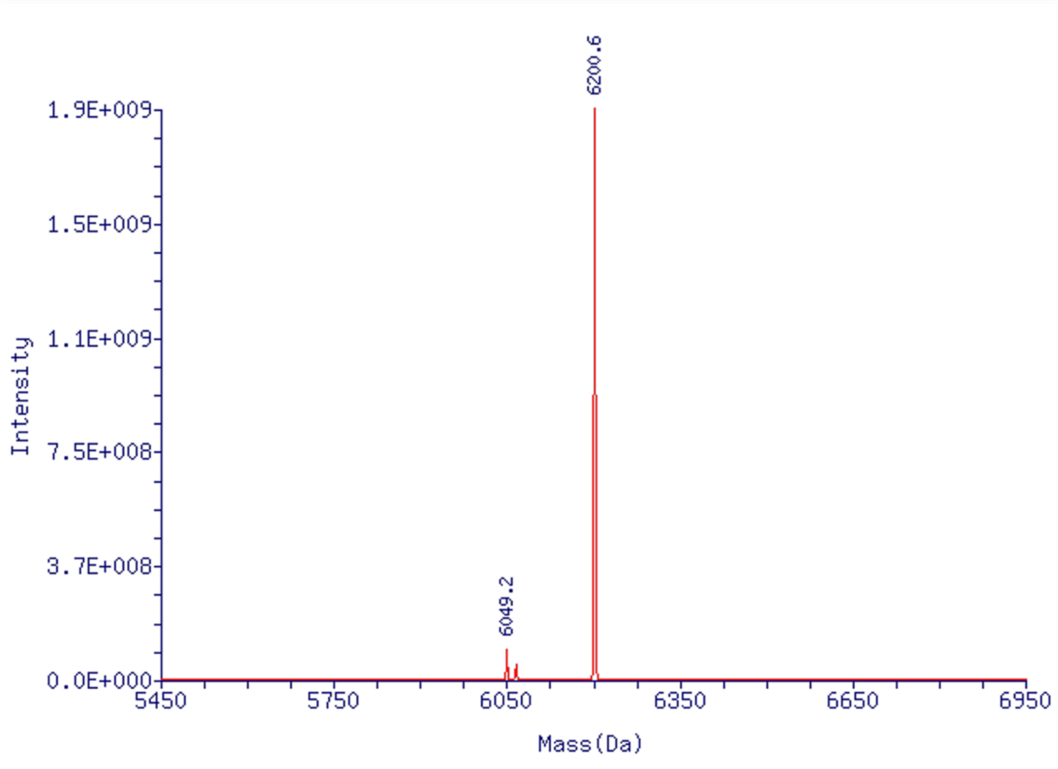


Supplementary Fig. 25. ESI-MS spectrum of DNA-$Fe_3$; calculated mass: 6200.67.

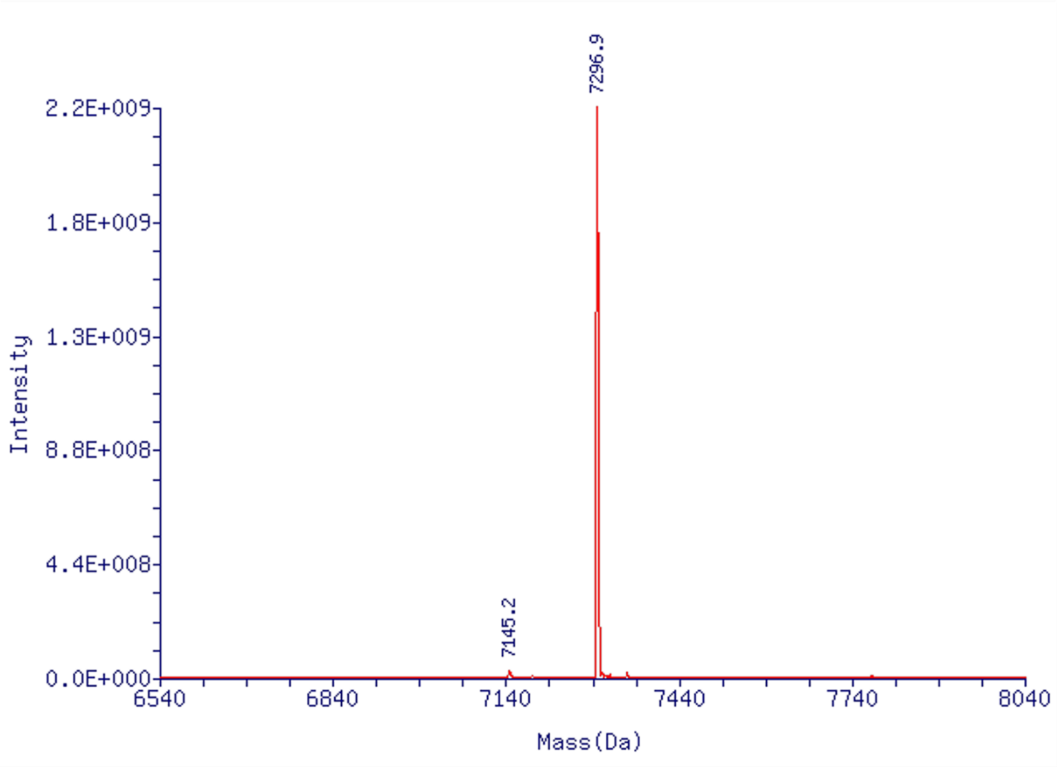


Supplementary Fig. 26. ESI-MS spectrum of DNA-$Fe_6$; calculated mass: 7296.00.

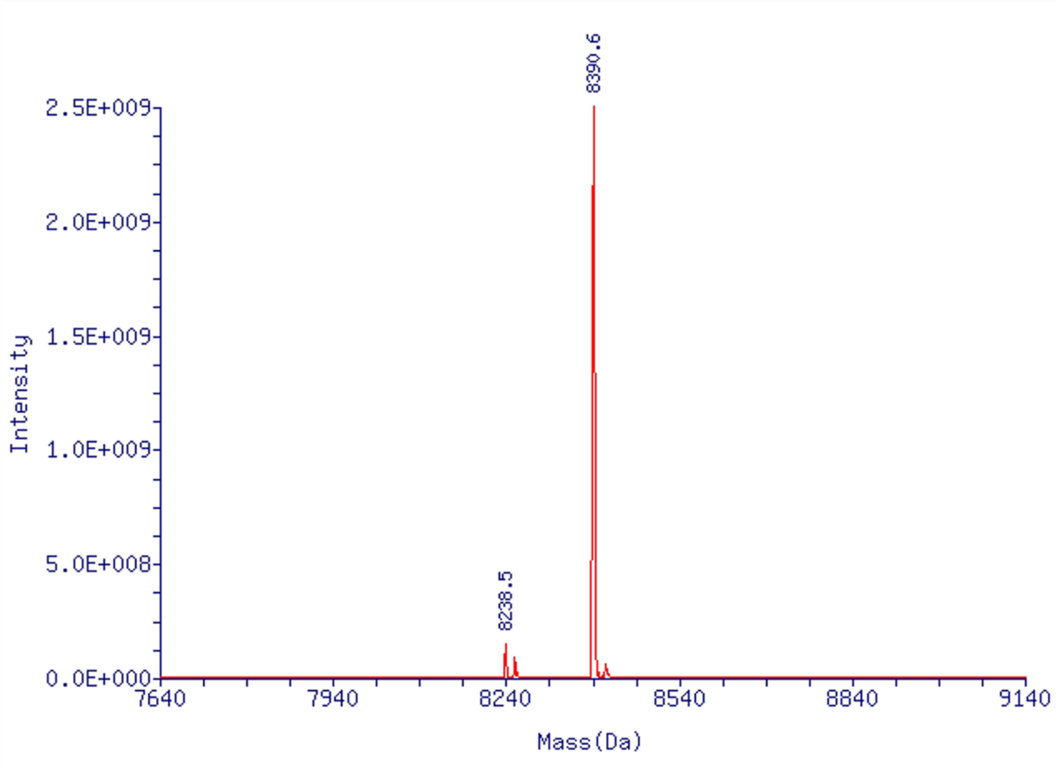


Supplementary Fig. 27. ESI-MS spectrum of DNA-$Fe_9$; calculated mass: 8391.33.

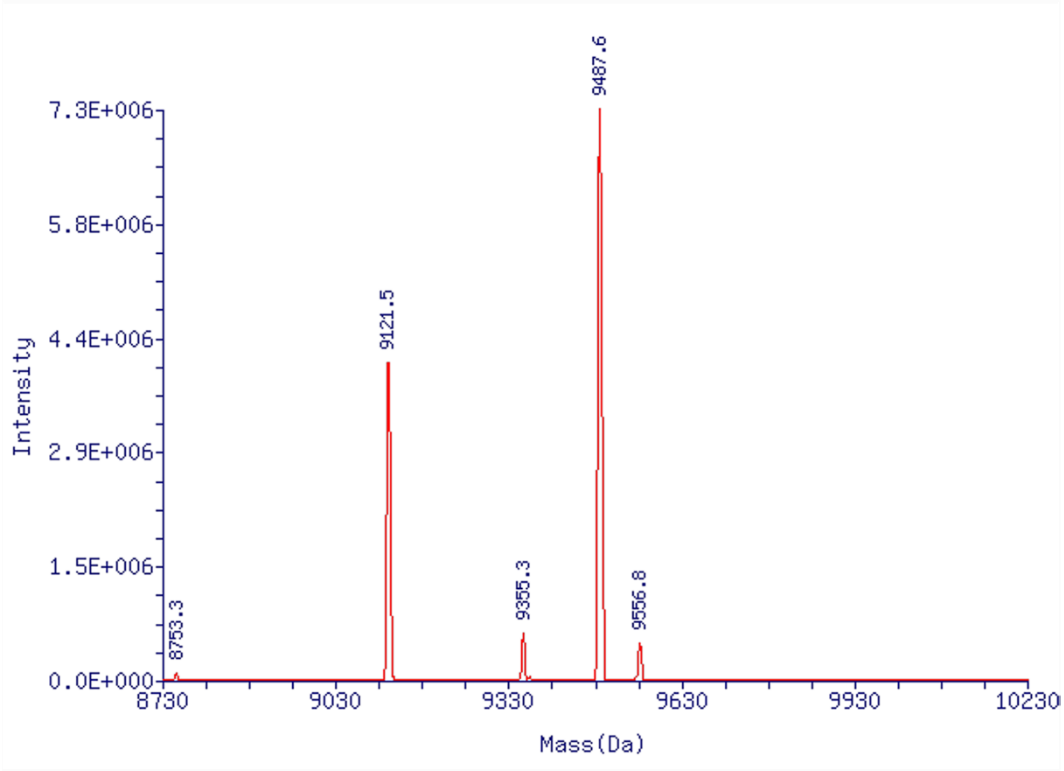


Supplementary Fig. 28. ESI-MS spectrum of DNA-$Fe_{12}$; calculated mass: 9486.66.

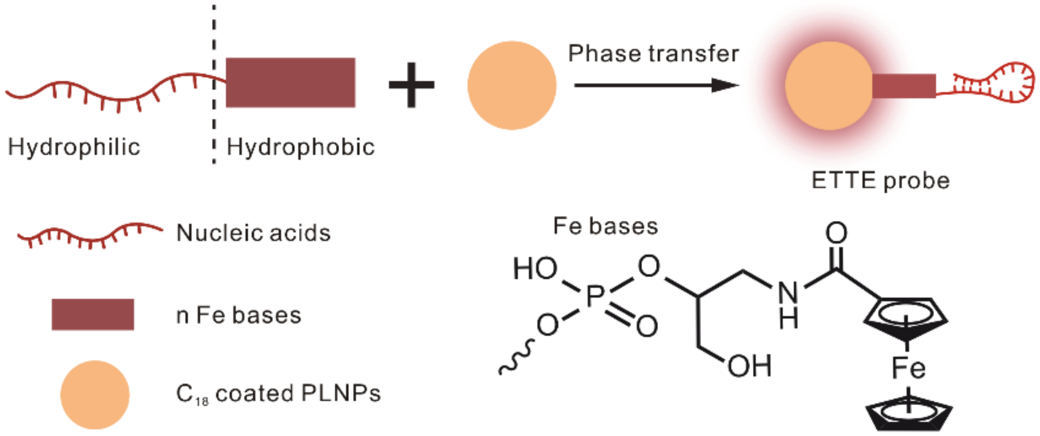


Supplementary Fig. 29. Fabrication schematics of the ETTE nanoprobes with Fe-bases (not to scale).

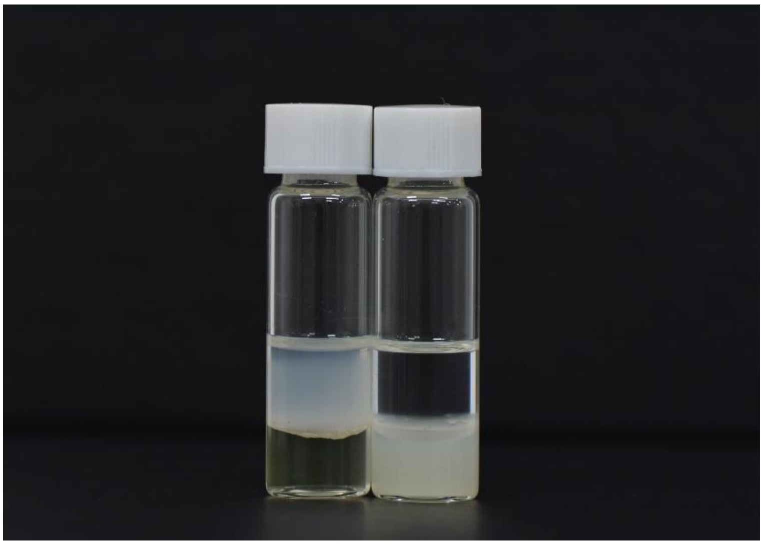

Supplementary Fig. 30. Digital picture of $ZnGa_2O_4$ and ETTE nanoprobes before (left) and after (right) phase transfer reaction, respectively.

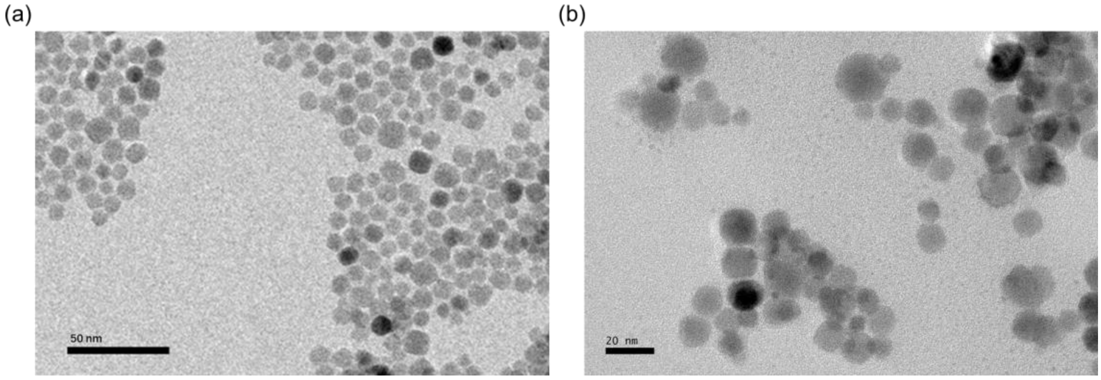


Supplementary Fig. 31. TEM images of ETTE nanoprobes before (a) and after (b) optimization with ferrocene-DNA polymer chains. Scale bar: (a) 50 nm; (b) 20 nm. The TEM imaging experiments were repeated independently three times and similar results were obtained.

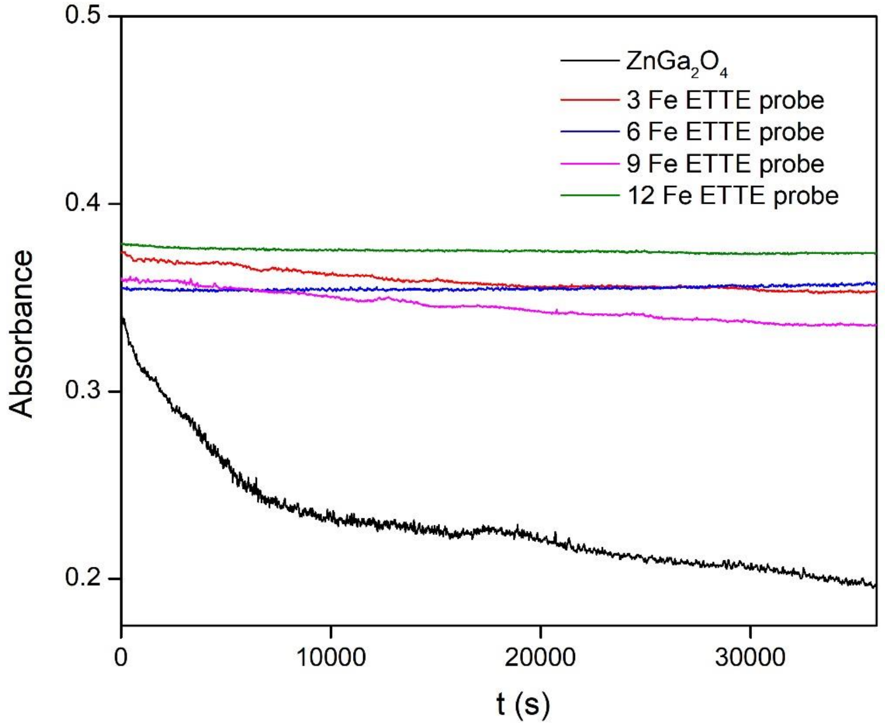


Supplementary Fig. 32. Kinetic measurement of dispersibility of ETTE nanoprobe with varying Fe-base modifications ($ZnGa_2O_4$, 3 Fe ETTE nanoprobe, 6 Fe ETTE nanoprobe, 9 Fe ETTE nanoprobe, 12 Fe ETTE nanoprobe), where precipitation of ETTE nanoprobe occurred in phosphate buffer solution (pH 6.98).

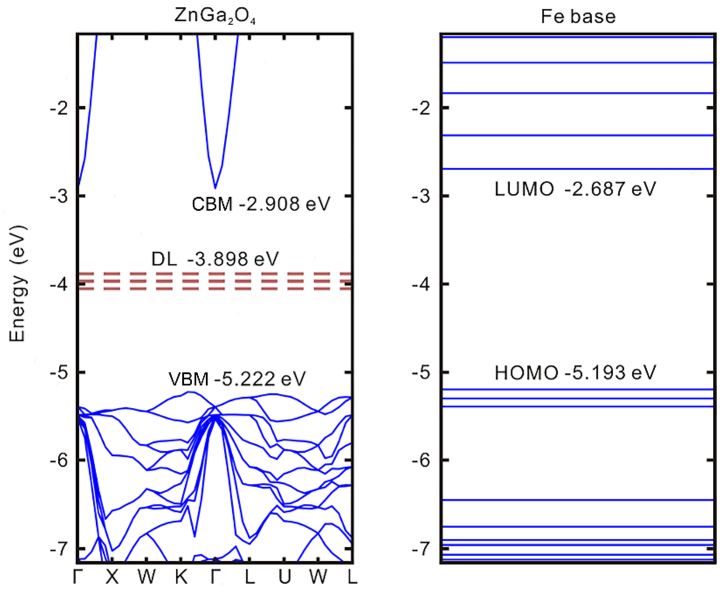


Supplementary Fig. 33. The energetic structure of the ETTE nanoprobe calculated by first-principle simulation. CBM, conduction band minimum; VBM, valence band maximum; LUMO, lowest unoccupied molecular orbital; HOMO, highest occupied molecular orbital.

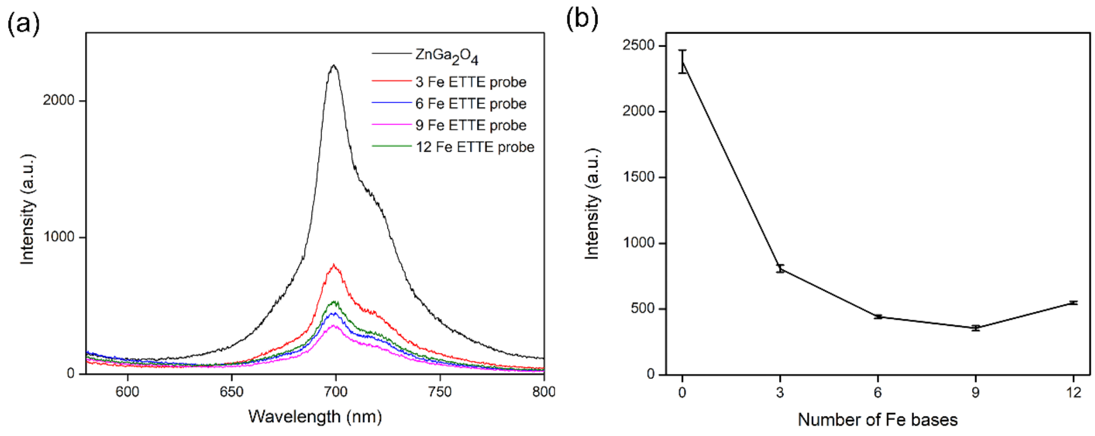


Supplementary Fig. 34. Fluorescence spectrum of ETTE nanoprobe with varying Fe-base modifications ($ZnGa_2O_4$, 3 Fe ETTE nanoprobe, 6 Fe ETTE nanoprobe, 9 Fe ETTE nanoprobe, 12 Fe ETTE nanoprobe). Data are presented as the mean values ± s.d.; n = 3 independent experiments.

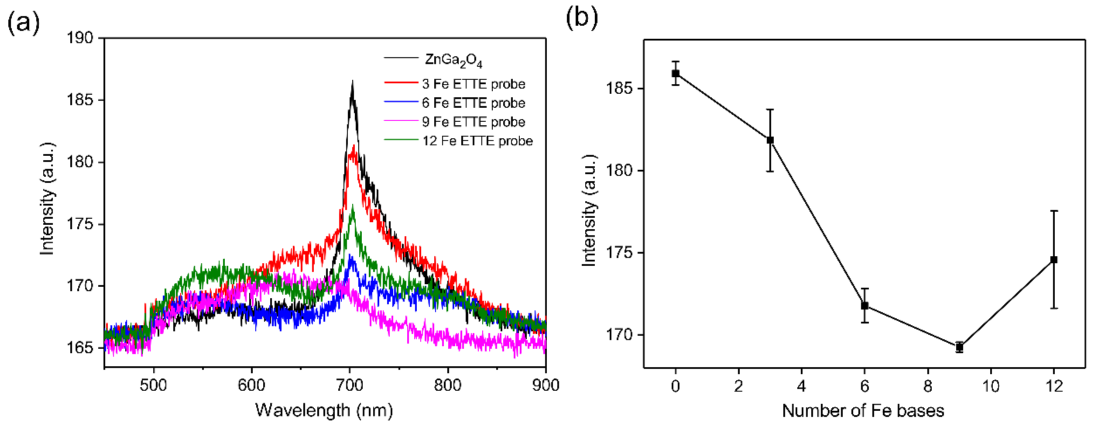


Supplementary Fig. 35. Single particle fluorescence spectrum of ETTE nanoprobe with varying Fe-base modifications ($ZnGa_2O_4$, 3 Fe ETTE nanoprobe, 6 Fe ETTE nanoprobe, 9 Fe ETTE nanoprobe, 12 Fe ETTE nanoprobe). Data are presented as the mean values ± s.d.; n = 3 independent experiments.

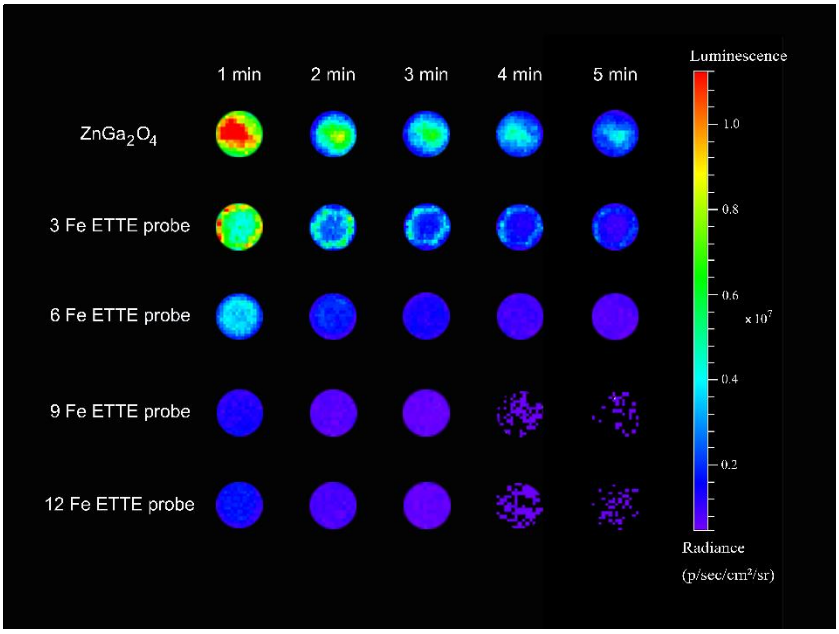


Supplementary Fig. 36. Afterglow images of ETTE nanoprobe with varying Fe-base modifications ($ZnGa_2O_4$, 3 Fe ETTE nanoprobe, 6 Fe ETTE nanoprobe, 9 Fe ETTE nanoprobe, 12 Fe ETTE nanoprobe). Color scale: $4.0 \times 10^5 – 1.6 \times 10^7$. The imaging experiments were repeated independently three times and similar results were obtained.

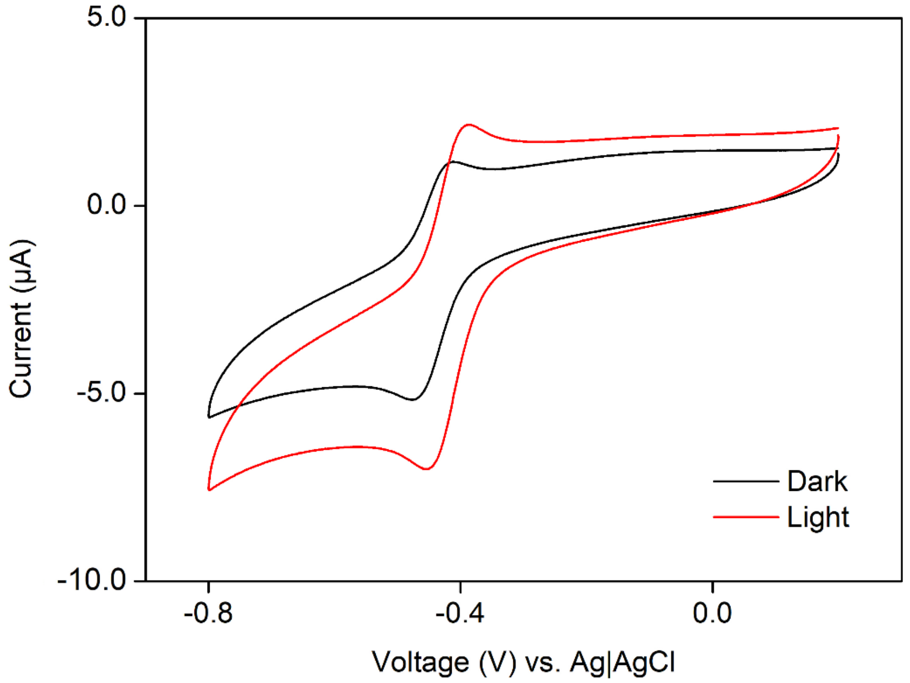


Supplementary Fig. 37. Cyclic voltammograms of the GOD/ETTE nanoprobe-modified electrode in oxygen-saturated phosphate buffer solution (pH 6.98) at a scan rate of 50 $mV\cdot s^{-1}$ under dark (black line) and light (red line) conditions.

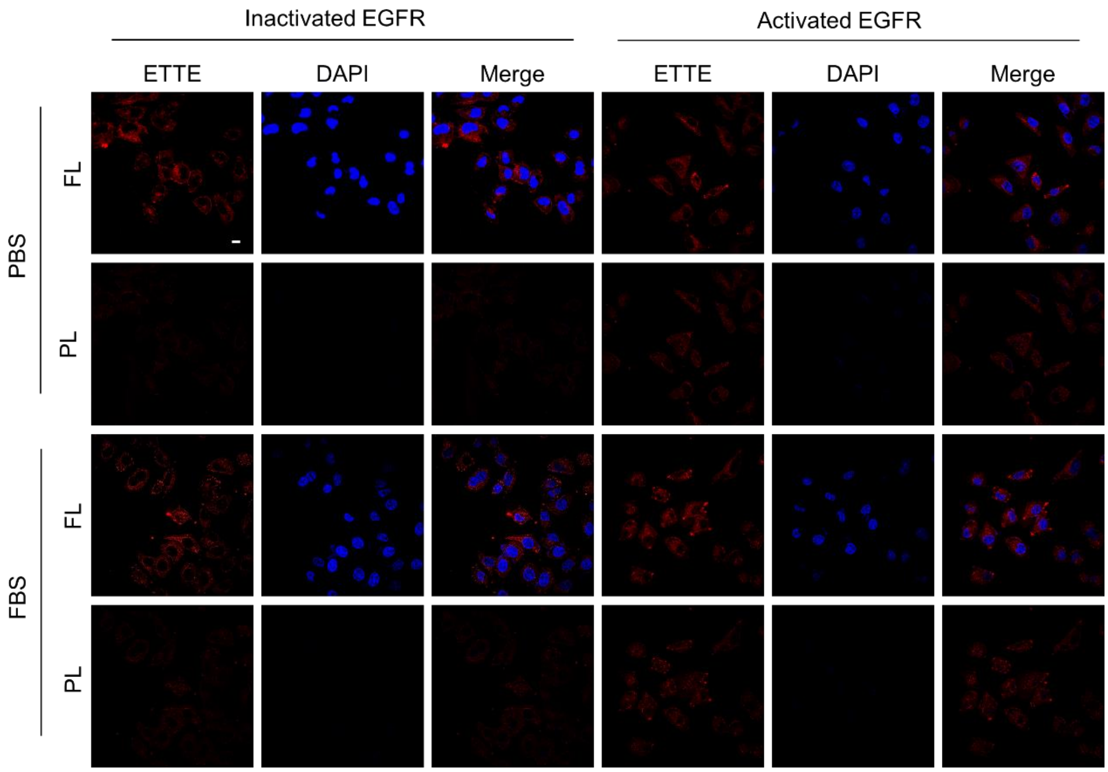


Supplementary Fig. 38. A549 cells were incubated with ETTE nanoprobes and DAPI for imaging. Fluorescence intensities and afterglow intensities of ETTE nanoprobes from A549 cells incubated without or with EGF in phosphate buffer solution (pH 6.98) and 10% FBS culture medium. Scale bar, 10 μm. The imaging experiments were repeated independently three times and similar results were obtained.

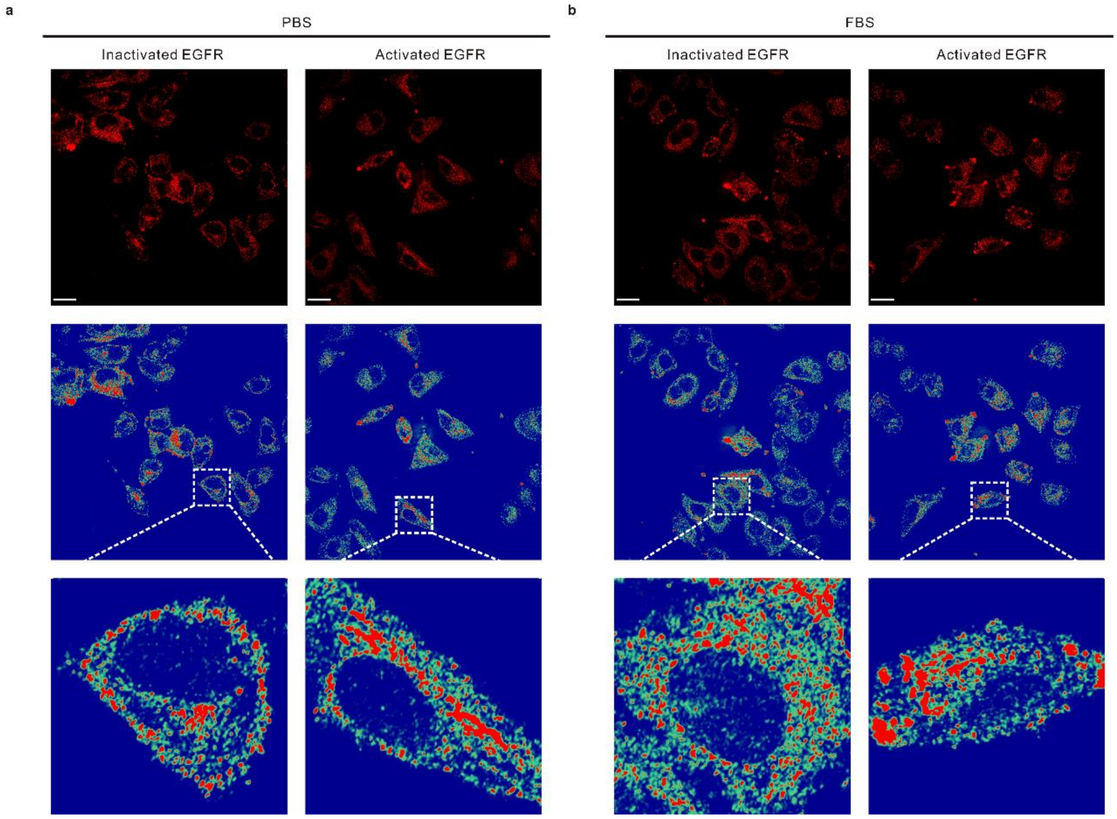


Supplementary Fig. 39. Fluorescence images and corresponding pseudo-color images of ETTE nanoprobes from A549 cells incubated without or with EGF in PBS (**a**) and in 10% fetal bovine serum (FBS) culture medium (**b**). Similar fluorescence intensities reflect comparable EGFR targeting capability of ETTE probe. Scale bar, 20 µm. The imaging experiments were repeated independently three times and similar results were obtained.

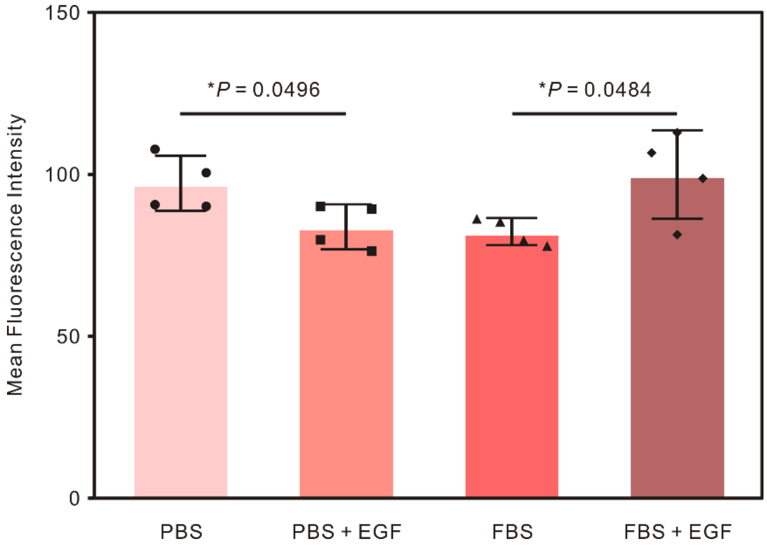


Supplementary Fig. 40. The bar graph shows the relative fluorescence intensities of ETTE nanoprobes shown in Supplementary Fig. 39. Data are presented as the mean values ± s.d.; unpaired two-tailed Student's t-test; n = 4 independent experiments.

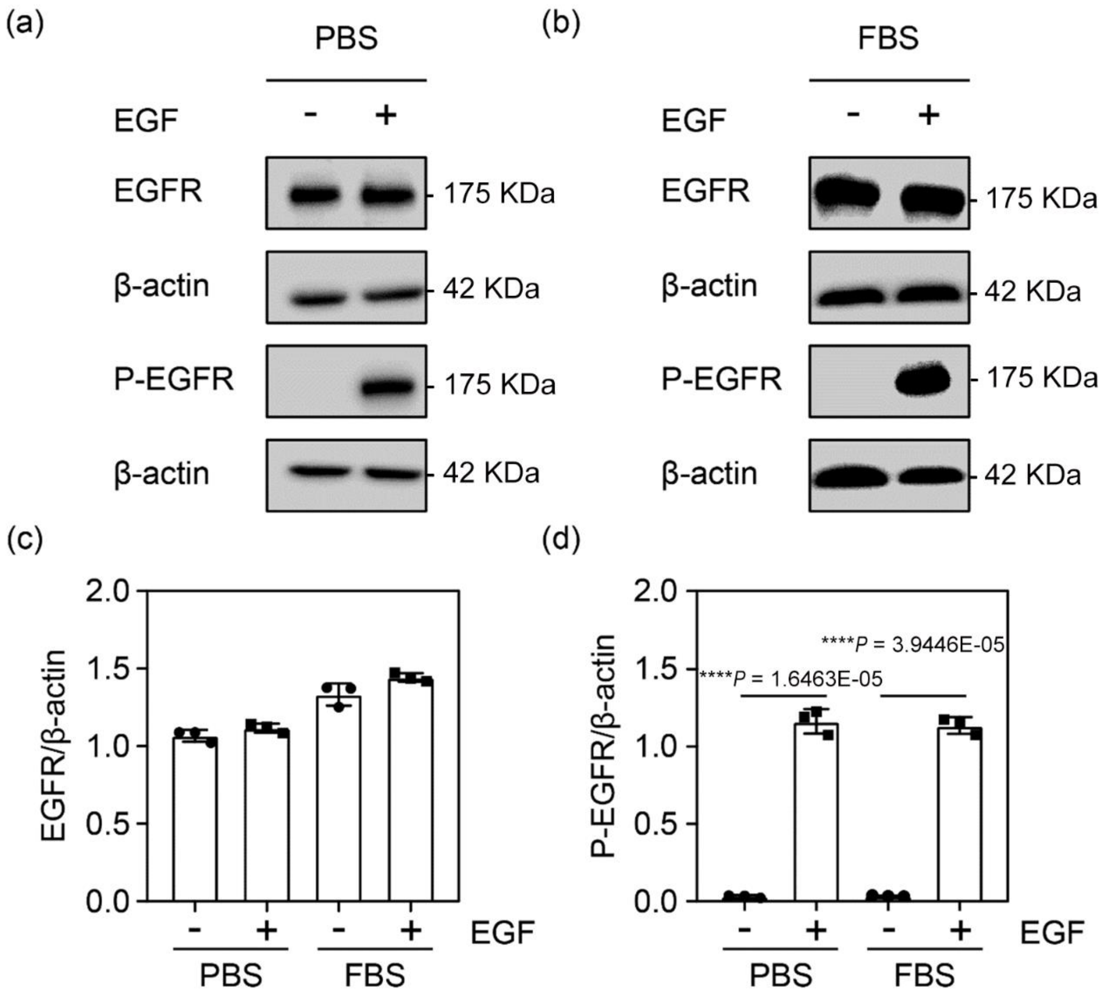


Supplementary Fig. 41. Western blot analysis on expression of EGFR, P-EGFR and β-catenin protein in A549 cells incubated without or with EGF in phosphate buffer solution (a) and 10% FBS culture medium (b). The experiments were repeated independently three times and similar results were obtained. For the western blot quantification, the EGFR (c) and P-EGFR (d) bands were normalized to the β-catenin bands, respectively. Data are presented as the mean values ± s.d.; unpaired two-tailed Student's t-test; n = 3 independent experiments.

It was observed in western blot result that comparable EGFR bands could be observed, referring to the similar EGFR expression in A549 cells incubated without or with EGF. When it came to P-EGFR, the bands were clearly displayed in the EGF-treated cells and disappeared in the cells without EGF incubation.

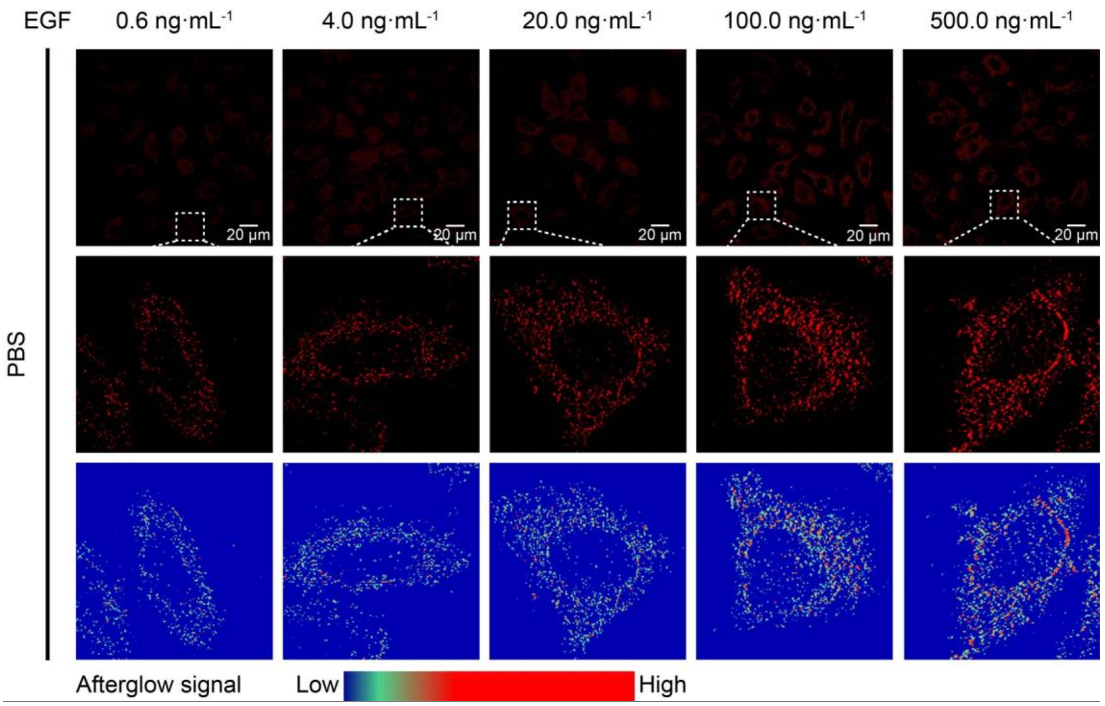


Supplementary Fig. 42. Afterglow images and corresponding pseudo-color images of ETTE nanoprobes from A549 cells incubated with various EGF concentrations range from 0.6 ng·mL$^{-1}$ to 500.0 ng·mL$^{-1}$ in PBS. Color scale ($\Delta F$ / $F_0$): 0 – 1. The imaging experiments were repeated independently three times and similar results were obtained. Cells were treated and incubated for 10 min. After washing, the cells were visualized by the confocal microscope. The laser of 635 nm and 365 nm were used as the excitation sources, afterglow signal from the ETTE probe was obtained at 1.0 s after excitation. At a given concentration of EGF, the afterglow signal of ETTE was used to visualize the activity of EGFR.

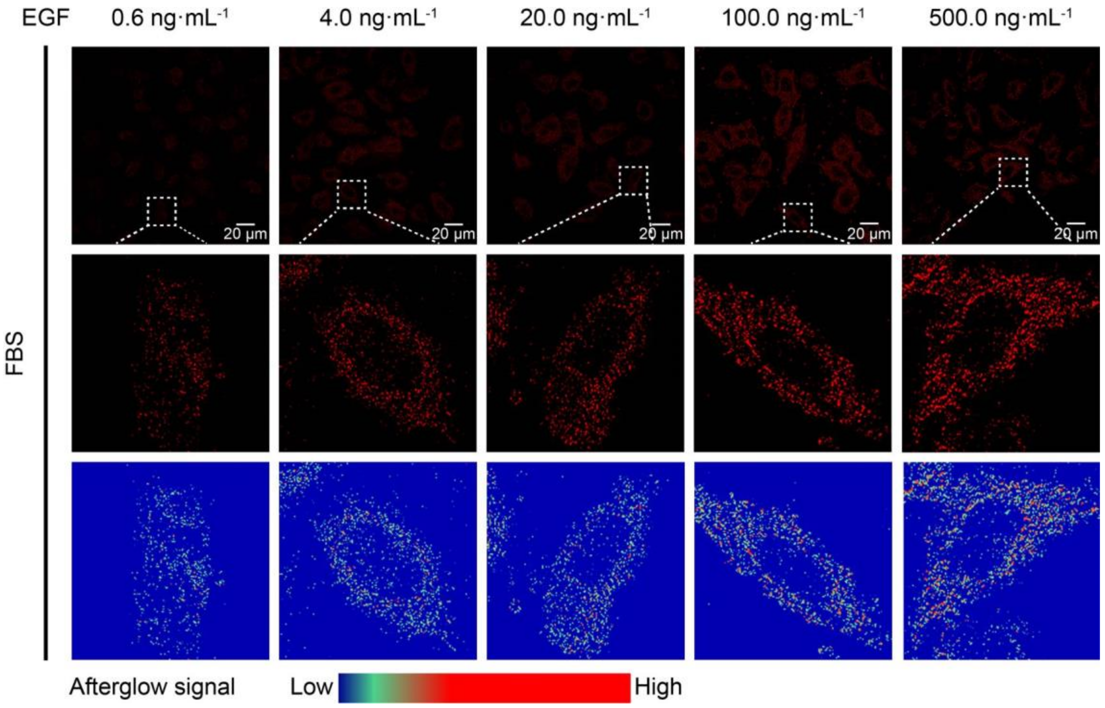


Supplementary Fig. 43. Afterglow images and corresponding pseudo-color images of ETTE nanoprobes from A549 cells incubated with various EGF concentrations range from 0.6 ng·mL$^{-1}$ to 500.0 ng·mL$^{-1}$ in FBS. Color scale ($\Delta F$ / $F_0$): 0 – 1. The imaging experiments were repeated independently three times and similar results were obtained. Cells were treated and incubated for 10 min. After washing, the cells were visualized by the confocal microscope. The laser of 635 nm and 365 nm were used as the excitation sources, afterglow signal from the ETTE probe was obtained at 1.0 s after excitation. At a given concentration of EGF, the afterglow signal of ETTE was used to visualize the activity of EGFR.

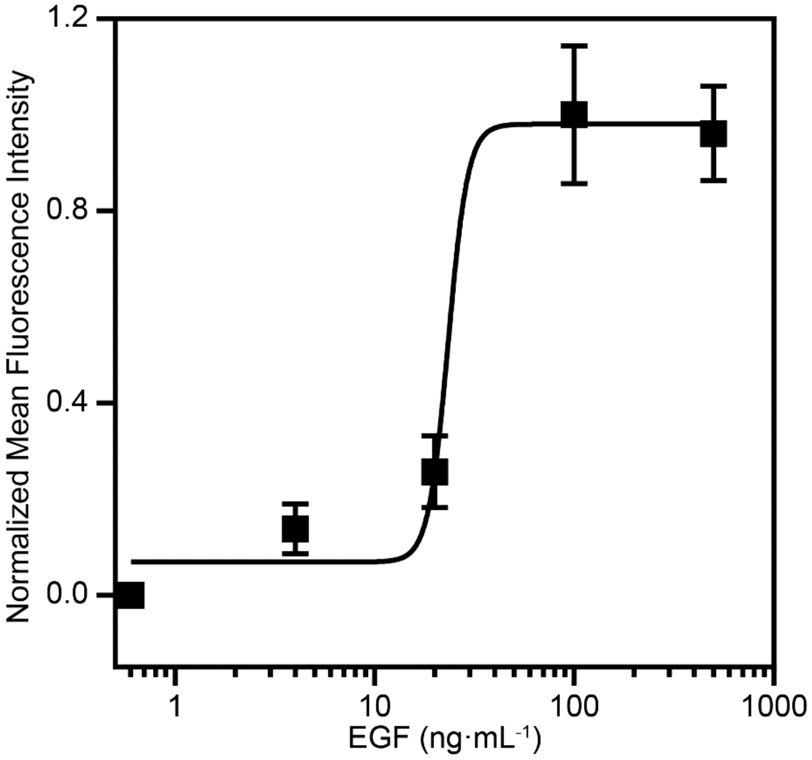


Supplementary Fig. 44. Dose-dependent response plot of ETTE probe to EGF application shown in Supplementary Fig. 42 yielded $EC_{50}$ = 23.33 ng·mL$^{-1}$ in PBS. Data are presented as the mean values ± s.d.; n = 4 independent experiments.

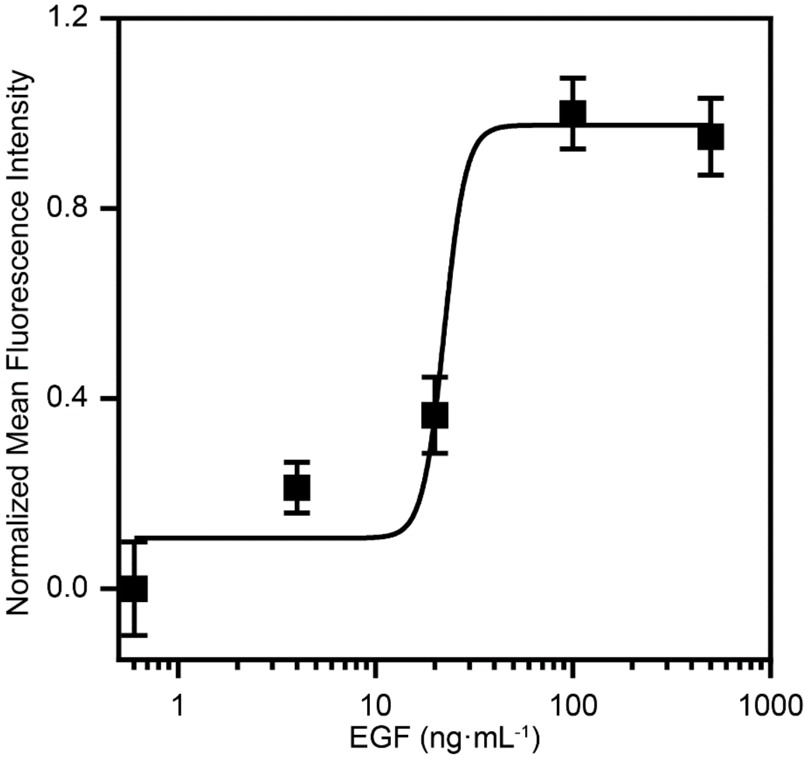


Supplementary Fig. 45. Dose-dependent response plot of ETTE probe to EGF application shown in Supplementary Fig. 43 yielded $EC_{50}$ = 22.29 ng·mL$^{-1}$ in FBS. Data are presented as the mean values ± s.d.; n = 4 independent experiments.

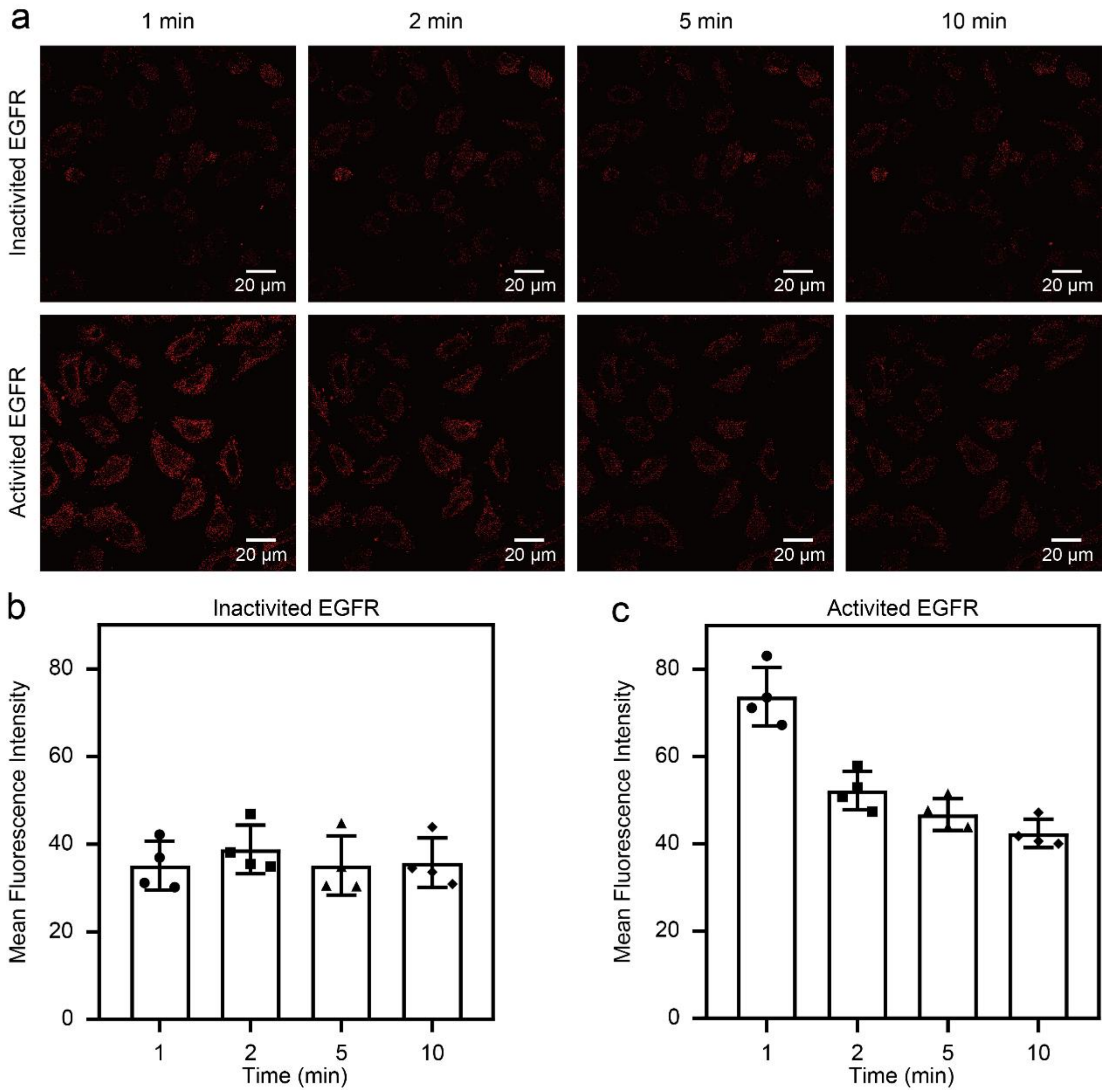


Supplementary Fig. 46. (a) Luminescence decay images of EGFR-inactivated and EGFR-activated cells. Afterglow signals of cells were continuously captured after excitation with UV lamp for 2 min. Scale bar: 20 μm. For the mean fluorescence intensity quantification, the EGFR-inactivated (b) and EGFR-activated cells (c) were calculated using ImageJ, respectively. Data are presented as the mean values ± s.d.; n = 4 independent experiments.

The afterglow signals of the EGFR-activated cells gradually weakened over time, while the afterglow signals of EGFR-inactivated cells kept low intensity. Compared with the EGFR-inactivated group, the cells in EGFR-activated group can be clearly imaged from 1 min to 5 min after excitation. While for 10 min after excitation, afterglow signals in the EGFR-activated group were still stronger than those in the EGFR-inactivated group.

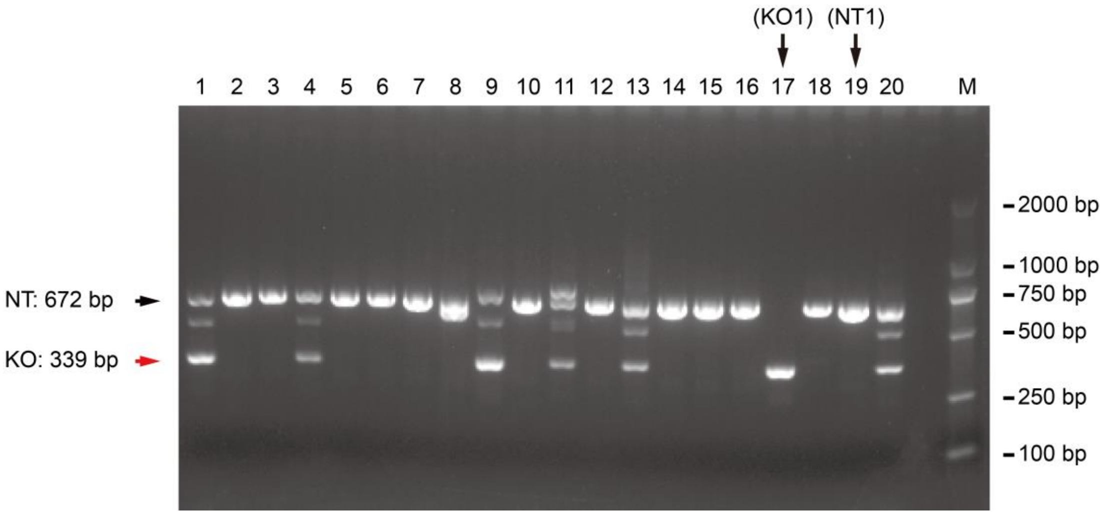


Supplementary Fig. 47. Agarose gel electrophoresis of PCR-based genotyping for identification of EGFR knockout clones of A549 cells from American Type Culture Collection (ATCC). A representative image of three biologically independent samples from each group is shown. Lane M: molecular weight standard marker. Lane #19: NT1 represents non-targeting control of A549 cells from ATCC. Lane #17: KO1 represents the EGFR knockout clone of A549 cells from ATCC. The forward primer of PCR was CTACCACCCACCCCTTTAAATTTCA, the reverse primer was CATTAGCTGGTAAAATGGCTTTCTC.

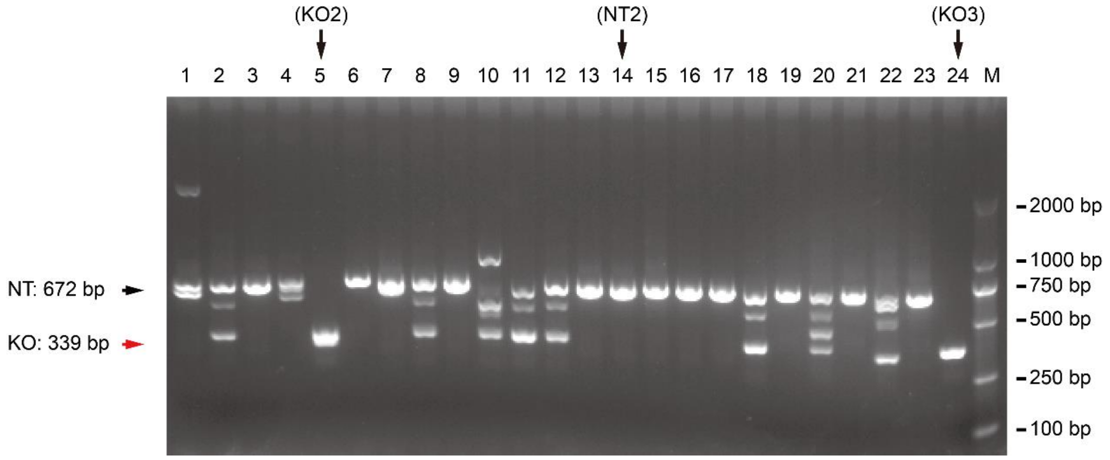


Supplementary Fig. 48. Agarose gel electrophoresis of PCR-based genotyping for identification of EGFR knockout clones of A549 cells from China Center for Type Culture Collection (CCTCC). A representative image of three biologically independent samples from each group is shown. Lane M: molecular weight standard marker. Lane #14: NT2 represents non-targeting control of A549 cells from CCTCC. Lane #5 and Lane #24: KO2 and KO3 represent two EGFR knockout clones of A549 cells from CCTCC. The forward primer of PCR was CTACCACCCACCCCTTTAAATTTCA, the reverse primer was CATTAGCTGGTAAAATGGCTTTCTC.

In order to ensure the accuracy and reproducibility of the experiment, A549 cells derived from two different sources, American Type Culture Collection (ATCC) and China Center for Type Culture Collection (CCTCC), were used for gene knockout, marked as A549-NT1, A549-NT2. Following transfection, cells were replated for single-cell cloning, propagated, and screened by a polymerase chain reaction (PCR) strategy designed to screen for EGFR knockout A549 cells. For A549-NT1, DNA sequencing of the PCR products from lane #1, #4, #9, #11, #13, #17, and #20 confirmed insertion or deletions leading to stop codons (Supplementary Fig. 46). The target gene in lane #17 have been knocked out and marked as A549-KO1. While for A549-NT2, the EGFR gene in A549-KO2 clone #5 and A549-KO3 clone #24 have been knocked out (Supplementary Fig. 48). A549-KO1 clone and A549-KO2 clone was further used for the western blot analysis.

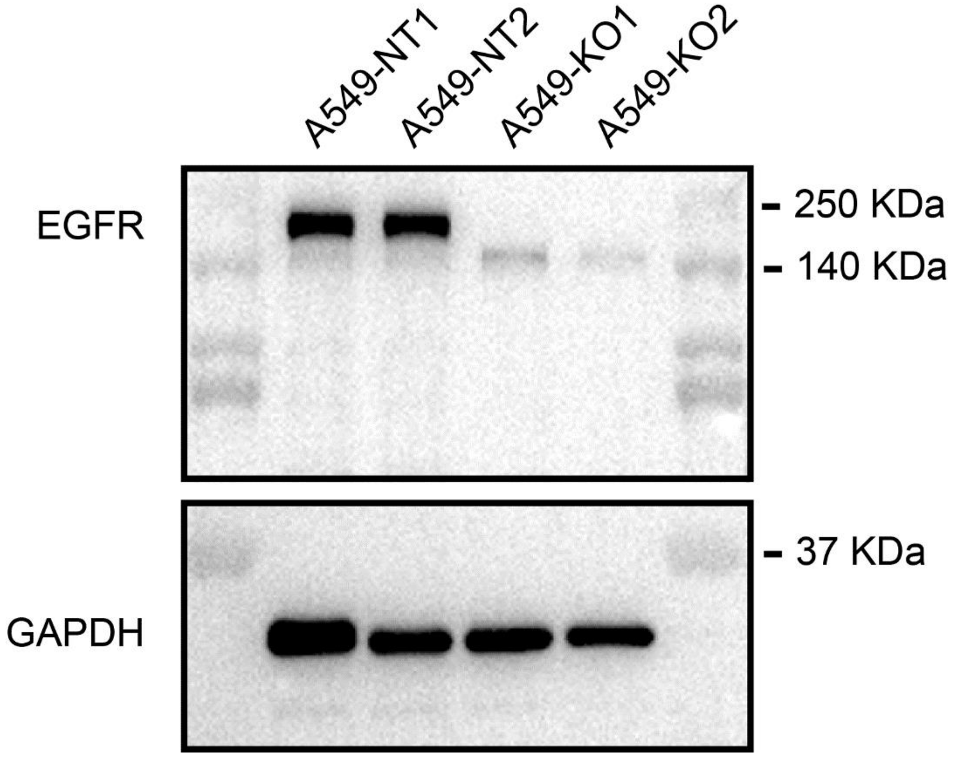


Supplementary Fig. 49. Western blot analysis confirmed loss of EGFR protein expression in A549-NT1, A549-NT2, A549-KO1, and A549-KO2 cells. GAPDH was used as a loading control. The experiments were repeated using three biologically independent samples.

As shown in Supplementary Fig. 49, western blot analysis by using anti-EGFR specific antibody revealed that EGFR proteins were less expressed in A549-KO1 and A549-KO2 cells than those in A549-NT1 and A549-NT2 cells. These results suggested that, for both two A549 cell strains, EGFR knockout A549 cells have been established, marking as A549-KO1 and A549-KO2 cell.

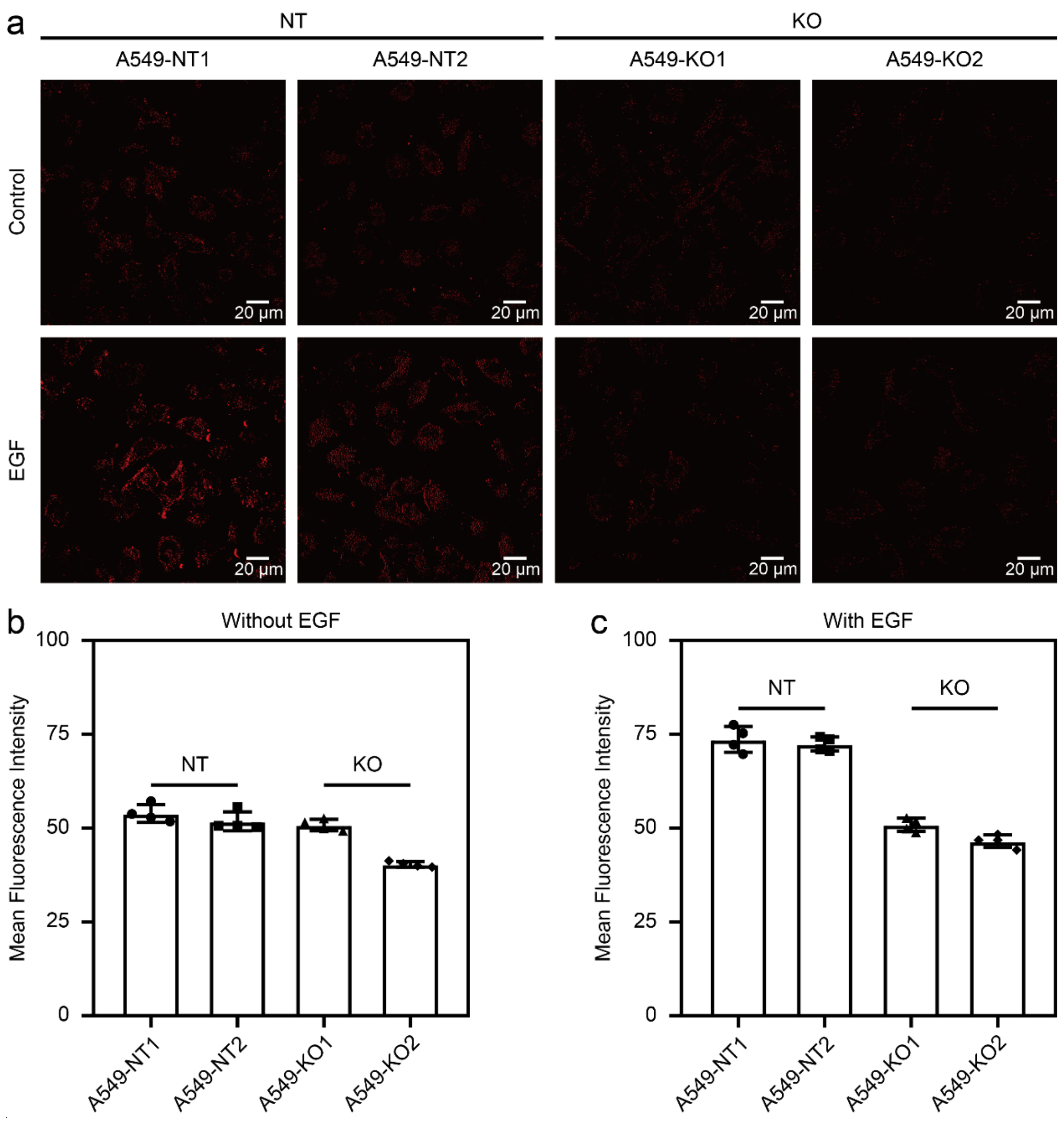


Supplementary Fig. 50. Confocal laser scanning microscopy images (a) and corresponding quantitative analysis (b, c) of ETTE probe-incubated A549-NT1, A549-NT2, A549-KO1 and A549-KO2 cells without (b) or with EGF treatment. Scale bars represent 20 μm. Data are presented as the mean values ± s.d.; n = 4 independent experiments.

Confocal laser scanning microscopy images showed that for the control cells A549-NT1 and A549-NT2, the afterglow signals of ETTE probe were significantly enhanced after adding EGF. While for EGFR knockout cells, the afterglow signal did not respond or were very insensitive to the treatment of EGF, suggesting that the signal change of ETTE probe is indeed EGFR-dependent.

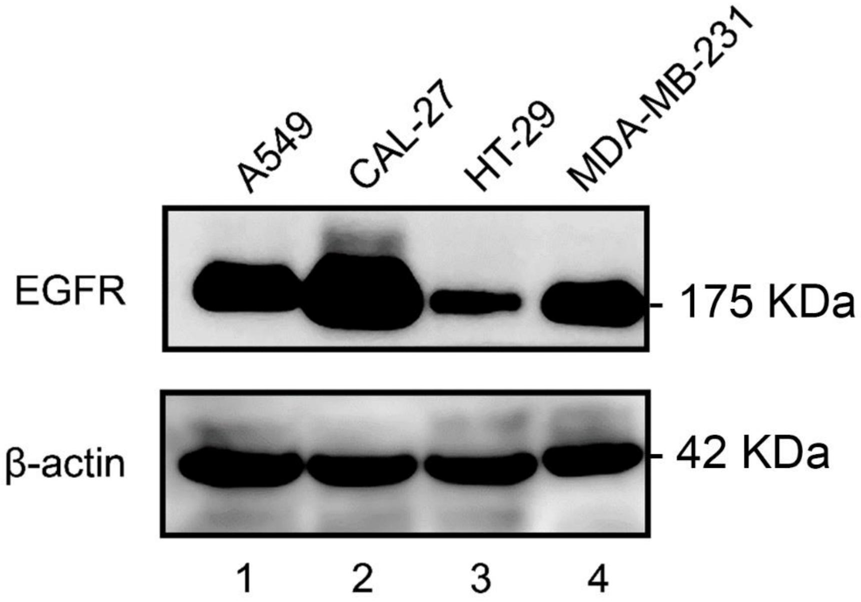


Supplementary Fig. 51. Western blot analysis on the expression of EGFR and β-actin protein in A549, CAL-27, and MDA-MB-231 cell lines. The experiments were repeated using three biologically independent samples.

Human tongue squamous cell carcinoma cell line (CAL-27), human colon cancer cell line (HT-29), and human breast cancer cell line (MDA-MB-231) were selected as EGFR high-expressing cell lines[3, 4]. Western blot analysis by using anti-EGFR specific antibody confirmed that EGFR proteins were highly expressed in A549, CAL-27, and MDA-MB-231 cells, thus these cell lines were chosen for the following experiments.

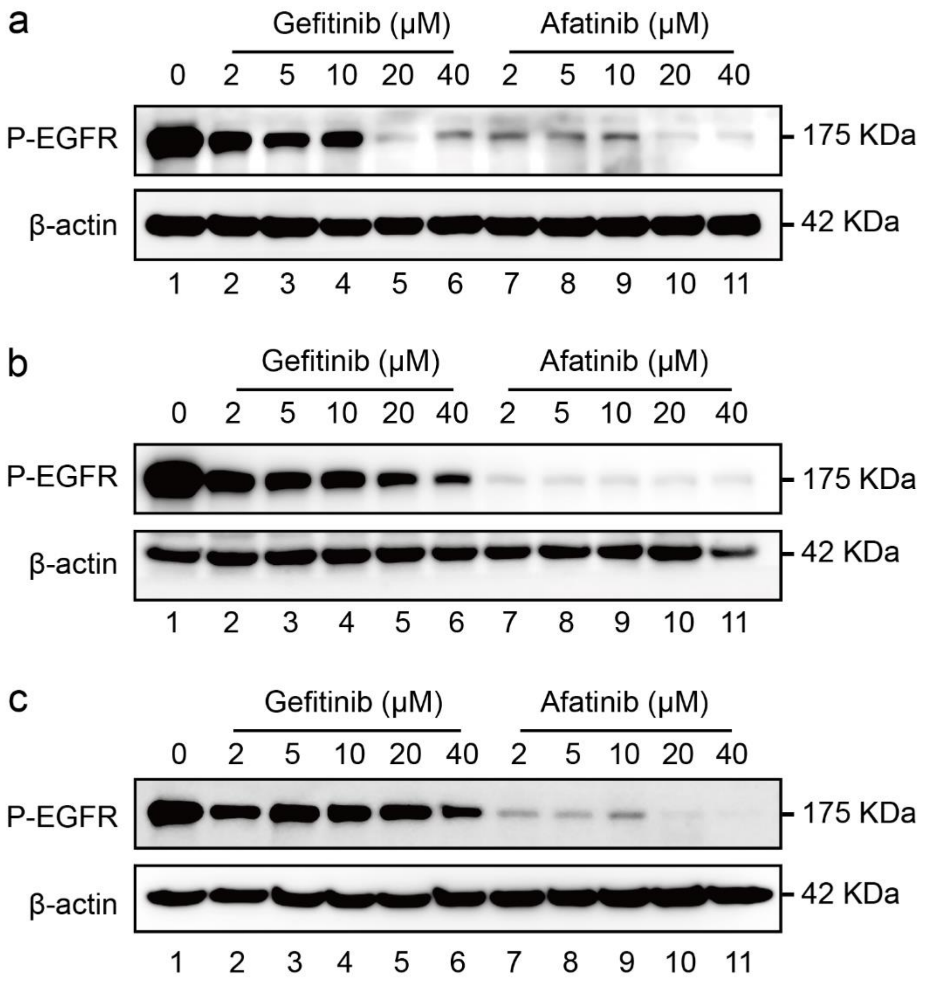


Supplementary Fig. 52. Suppression of P-EGFR in A549 (a), CAL-27 (b), and MDA-MB-231 (c) cell lines cells by EGFR tyrosine kinase inhibitor treatment. Cells were exposed to increasing concentrations of gefitinib or afatinib (2, 5, 10, 20 and 40 μM) for 3 h, then incubated with EGF for 30 min. The lysates were subjected to Western blot analysis to evaluate expression of P-EGFR and β-actin protein. The experiments were repeated using three biologically independent samples.

To determine the inhibitory effects of the two drugs on EGFR activity in different cell lines, various concentrations of the drugs were incubated with the cells before adding EGF. The expression of phosphorylated EGFR was analyzed by western blot, it was found that afatinib has a significant inhibitory effect on the activation of EGFR in three cell lines (Supplementary Fig. 52). Subsequently, afatinib was selected as an inhibitor of EGFR activity to study the imaging ability of ETTE probes on EGFR high-expressing cells treated with inhibitors.

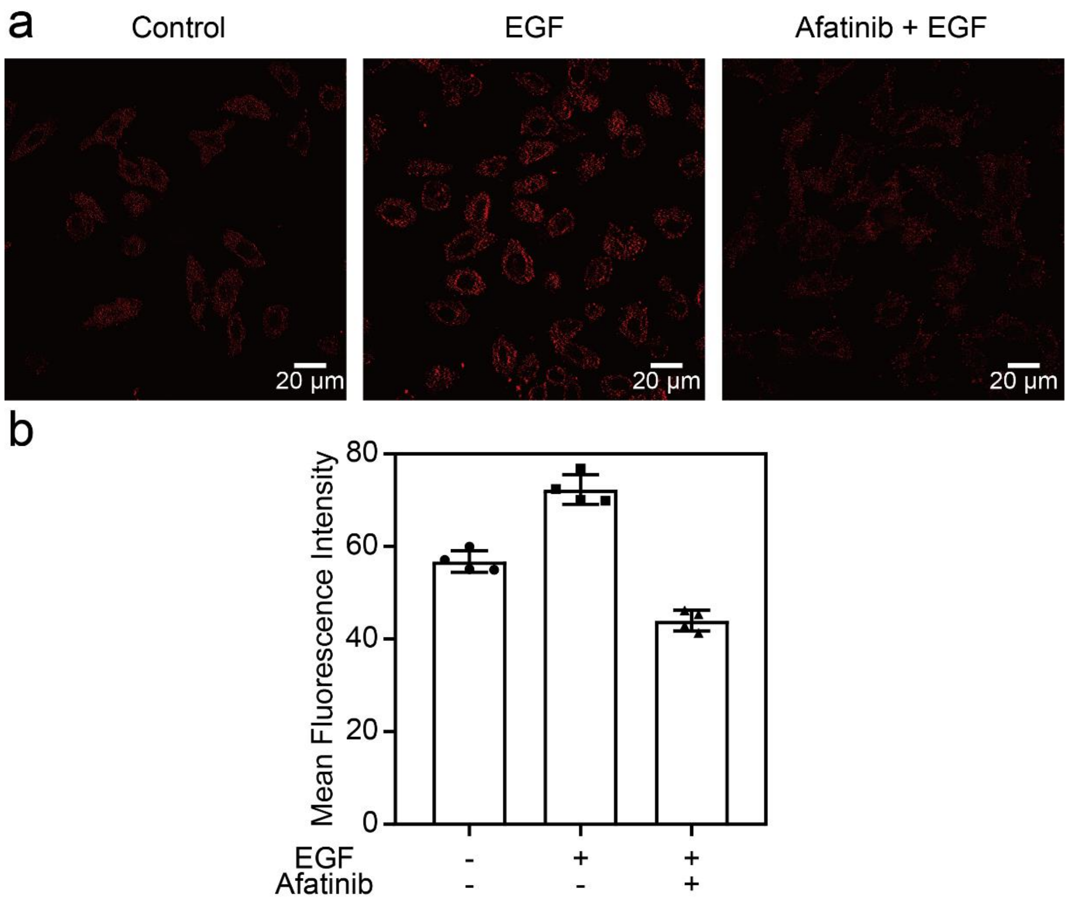


Supplementary Fig. 53. Confocal laser scanning microscopy images (a) and corresponding quantitative analysis (b) of A549 cells incubated with ETTE probe under different conditions: PBS control; addition of EGF; addition of EGF and afatinib. Scale bars represent 20 μm. Data are presented as the mean values ± s.d.; n = 4 independent experiments.

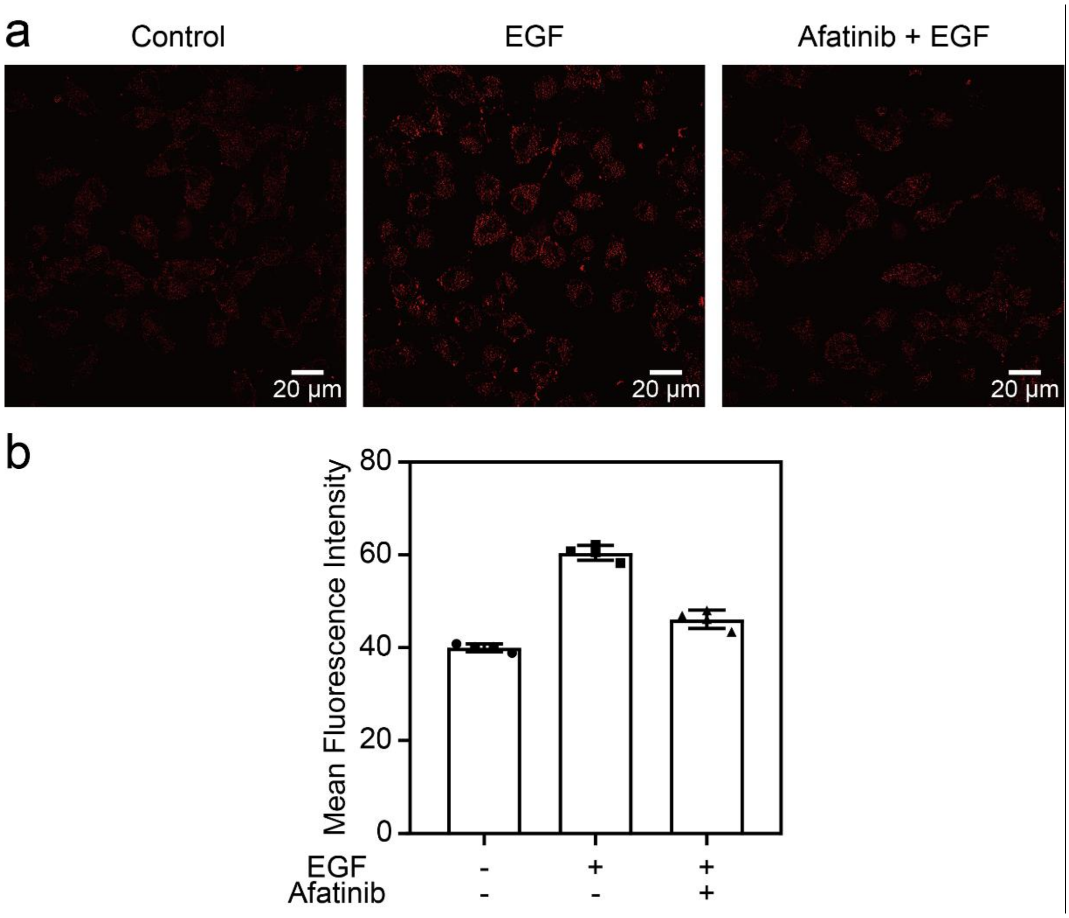


Supplementary Fig. 54. Confocal laser scanning microscopy images (a) and corresponding quantitative analysis (b) of CAL-27 cells incubated with ETTE probe under different conditions: PBS control; addition of EGF; addition of EGF and afatinib. Scale bars represent 20 μm. Data are presented as the mean values ± s.d.; n = 4 independent experiments.

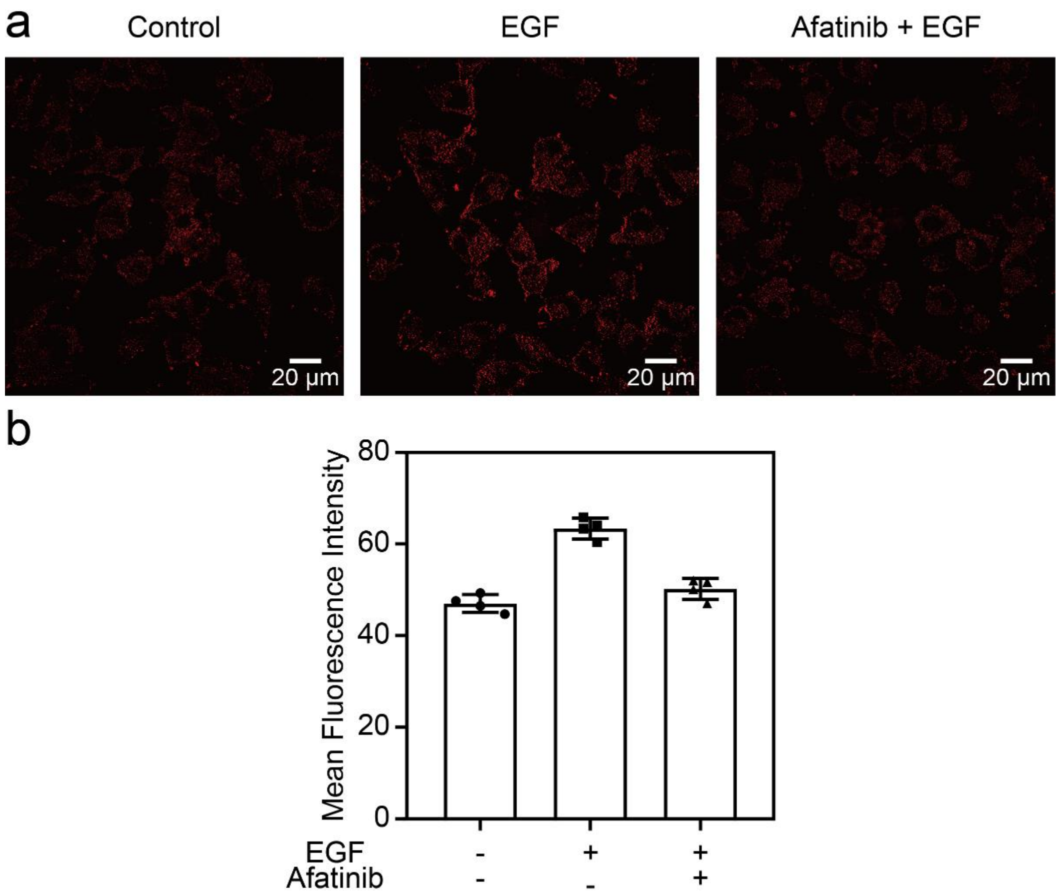


Supplementary Fig. 55. Confocal laser scanning microscopy images (a) and corresponding quantitative analysis (b) of MDA-MB-231 cells incubated with ETTE probe under different conditions: PBS control; addition of EGF; addition of EGF and afatinib. Scale bars represent 20 μm. Data are presented as the mean values ± s.d.; n = 4 independent experiments.

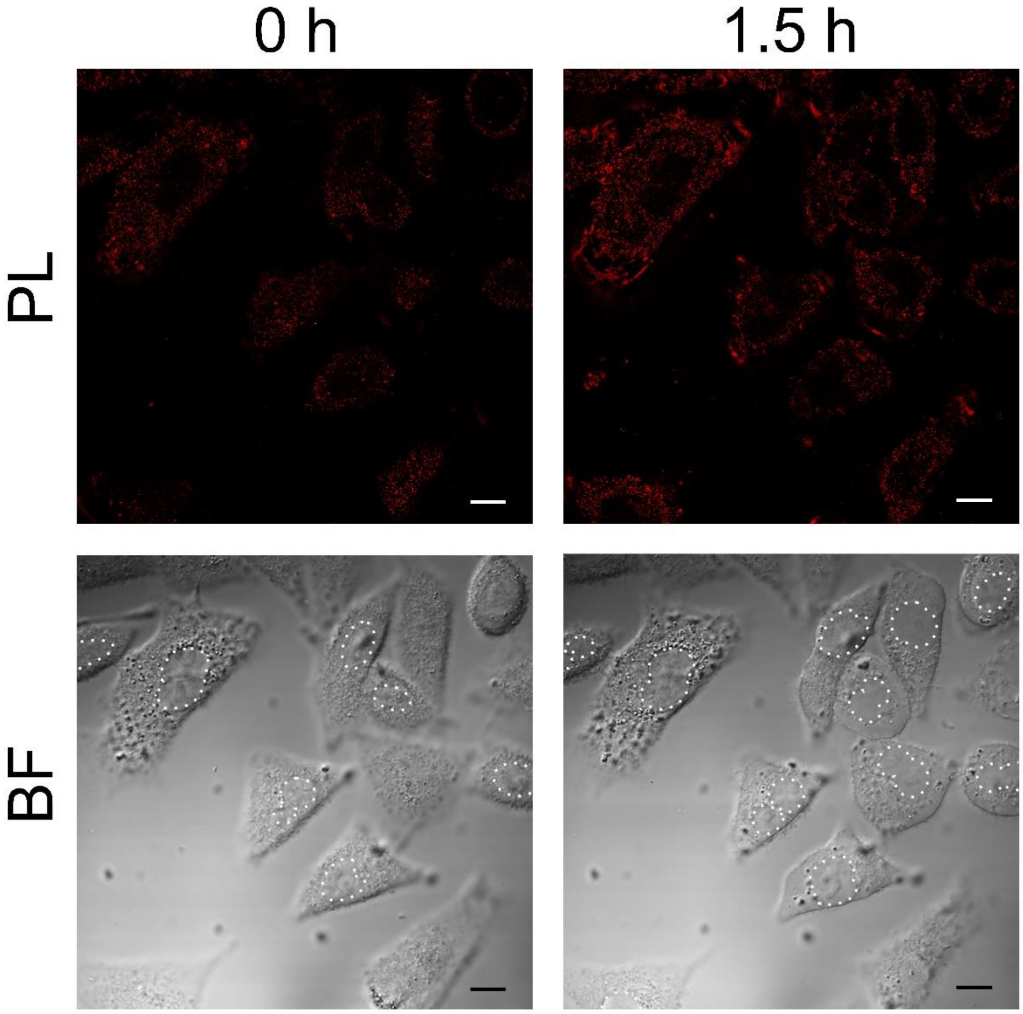


Supplementary Fig. 56. A549 cells were incubated with ETTE nanoprobe for imaging during cell division. Afterglow signal and bright field signal from A549 cells incubated with ETTE nanoprobes in 10% FBS culture medium. The imaging experiments were repeated independently three times and similar results were obtained. Higher intensities reflect higher EGFR signaling activity. Scale bar, 10 μm. During the division, daughter cells were linked together by a cytoplasmic bridge that extended over time, with the enhanced EGFR activity.

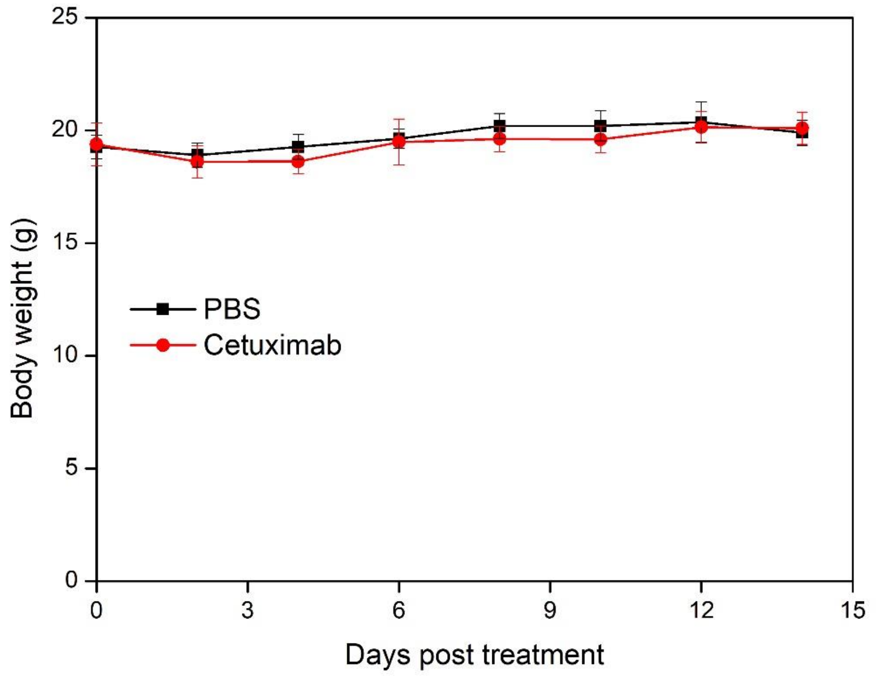


Supplementary Fig. 57. Graphical representation of the mean body weight from A549 xenograft mice treated without (square) or with (circle) cetuximab during 14 days. Each bar represents the mean values ± s.d.; n = 5 mice.

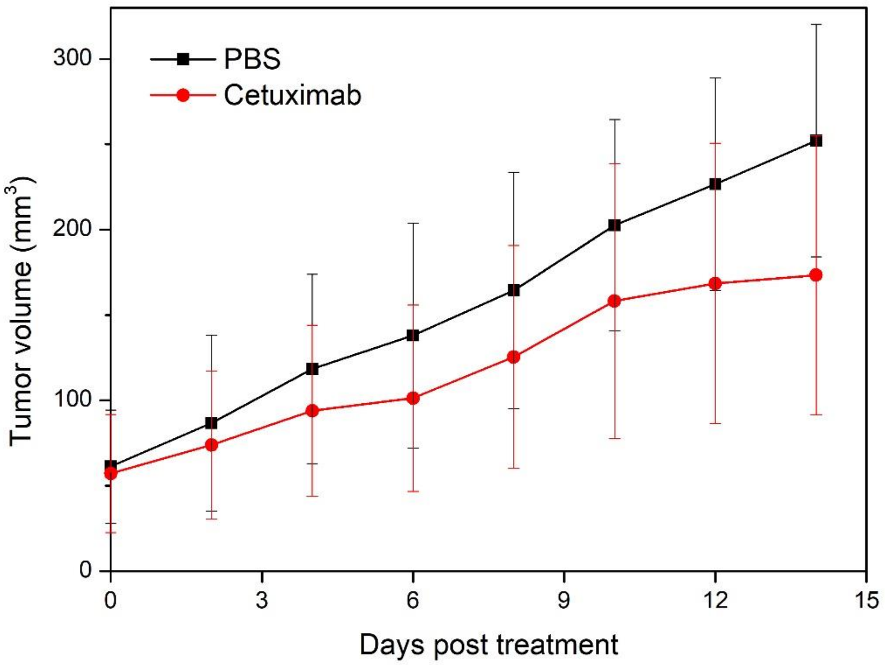


Supplementary Fig. 58. Graphical representation of the mean tumor volume from A549 xenograft mice treated without (square) or with (circle) cetuximab during 14 days. Each bar represents the mean values ± s.d.; n = 5 mice.

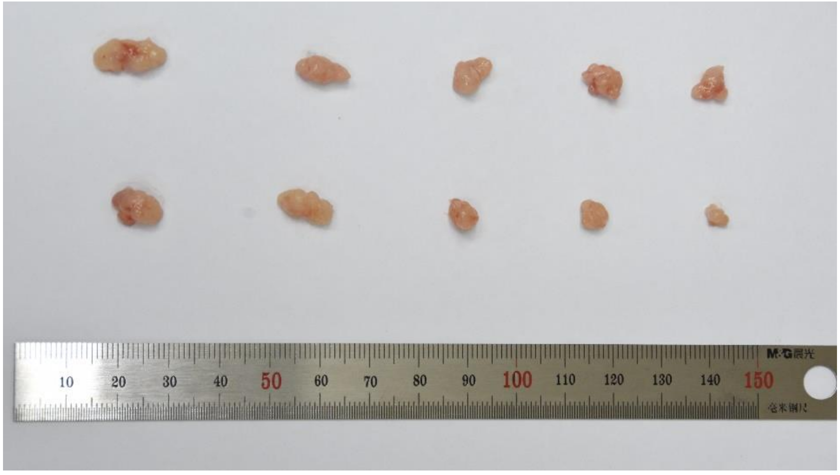

Supplementary Fig. 59. Images of the tumors harvested from the A549 xenograft mice treated without (a) or with (b) cetuximab after 14 days treatment, n = 5 mice.

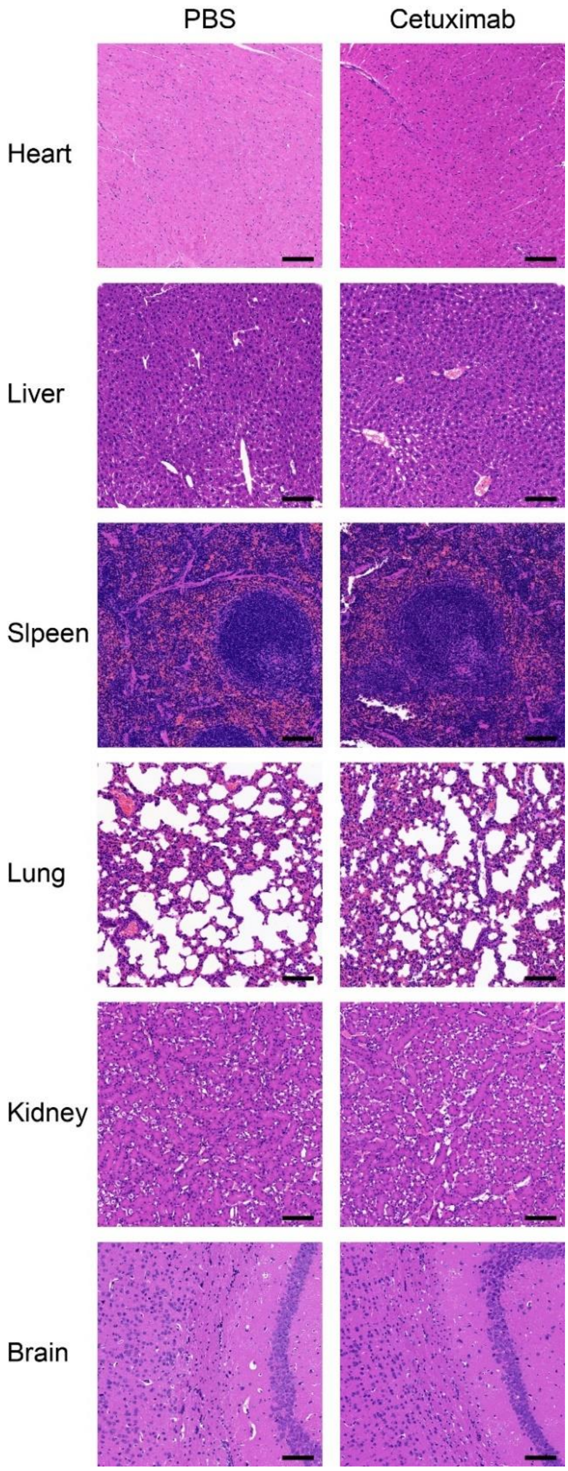


Supplementary Fig. 60. Histopathology images of dissected major organs (heart, liver, spleen, lung, kidney and brain) from mice treated without or with cetuximab for in vivo biosafety evaluation. Scale bar, 100 μm. A representative image of five biologically independent samples from each group is shown. The major organs of mice displayed no obvious abnormalities or lesions in both groups, indicating the limited adverse side effects to organs.

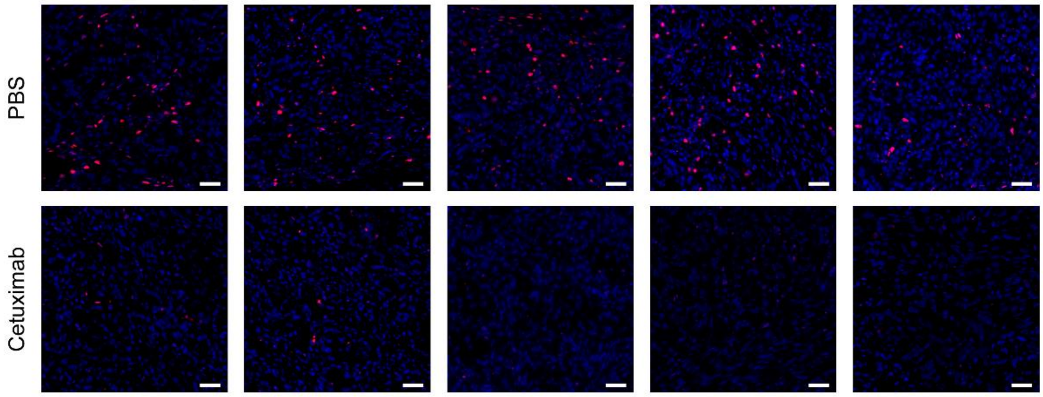


Supplementary Fig. 61. Ki67 staining images of the tumors harvested from the A549 xenograft mice treated with PBS or with cetuximab. Scale bar, 50 μm; n = 5 mice. These images could hardly display any red fluorescence of ki67 in the cetuximab-treated group compared with the PBS-treated group. As reported, the low expression of ki67 in cetuximab-treated group may owing to the suppression of tumor cell proliferation caused by the inhibited EGFR activity[5, 6].

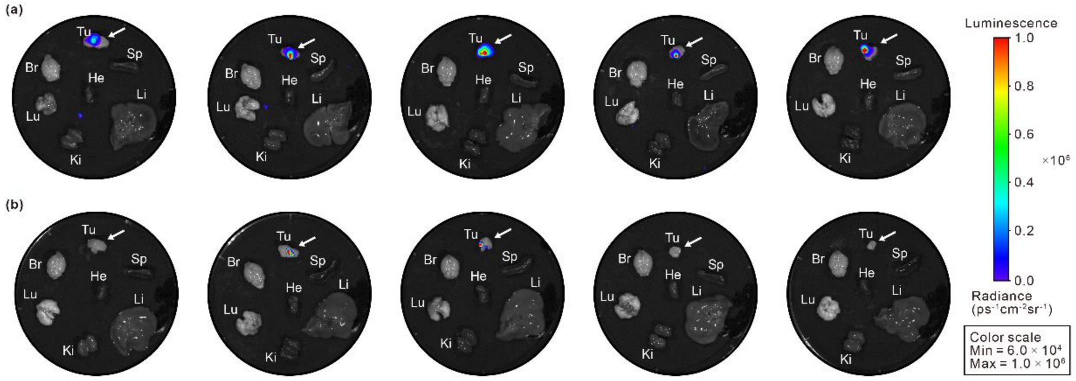


Supplementary Fig. 62. Ex vivo afterglow imaging of various organs and tumor tissue after final treatment without (a) and with cetuximab (b), respectively. Tu, tumor; Sp, spleen; Li, liver; Ki, kidney; Lu, lung; Br, brain; He, heart. Color scale: $6.0 \times 10^4 – 1.0 \times 10^6$; n = 5 mice.

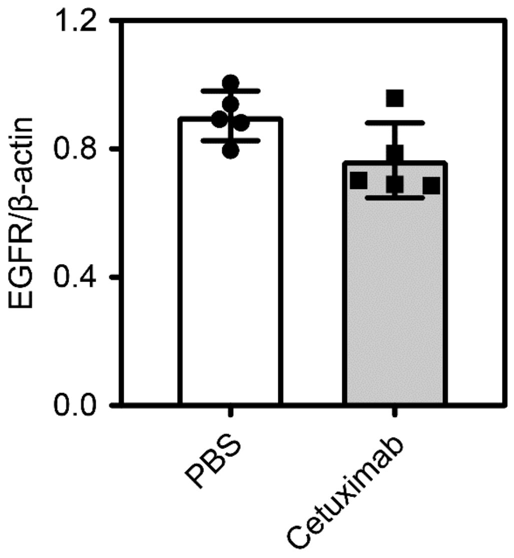


Supplementary Fig. 63. Quantitative analysis of EGFR in tumor sections observed by western blot analysis. Data are presented as mean values ± s.d., n = 5 mice.

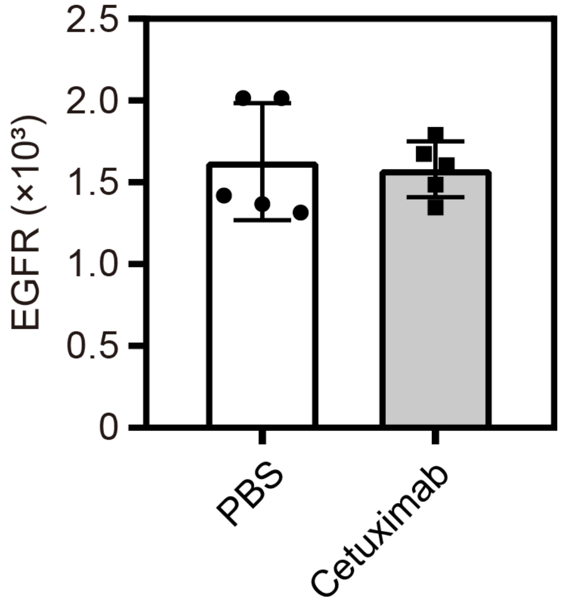


Supplementary Fig. 64. Quantitative analysis of EGFR in tumor sections observed by IHC analysis. Data are presented as mean values ± s.d., n = 5 mice.

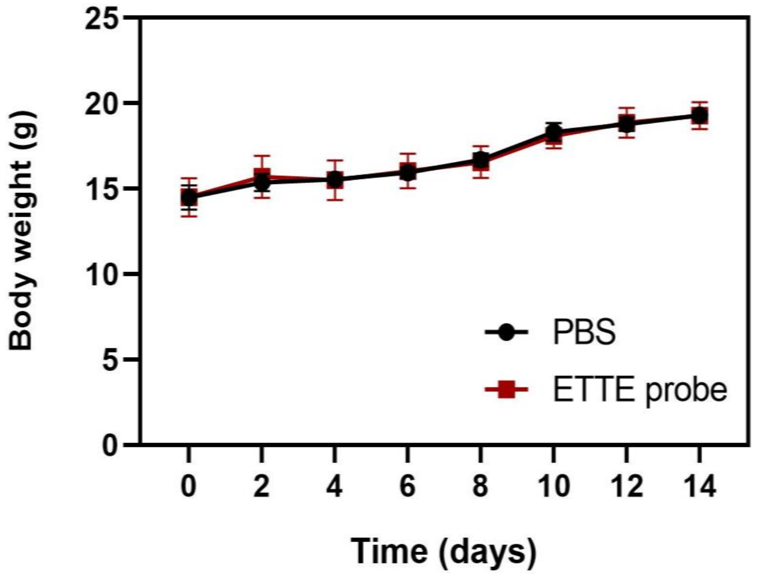


Supplementary Fig. 65. The body weights of BALB/c nude mice after injections with PBS and ETTE probe intravenously. Data are presented as the mean values ± s.d.; unpaired two-tailed Student's t-test; n = 5 mice.

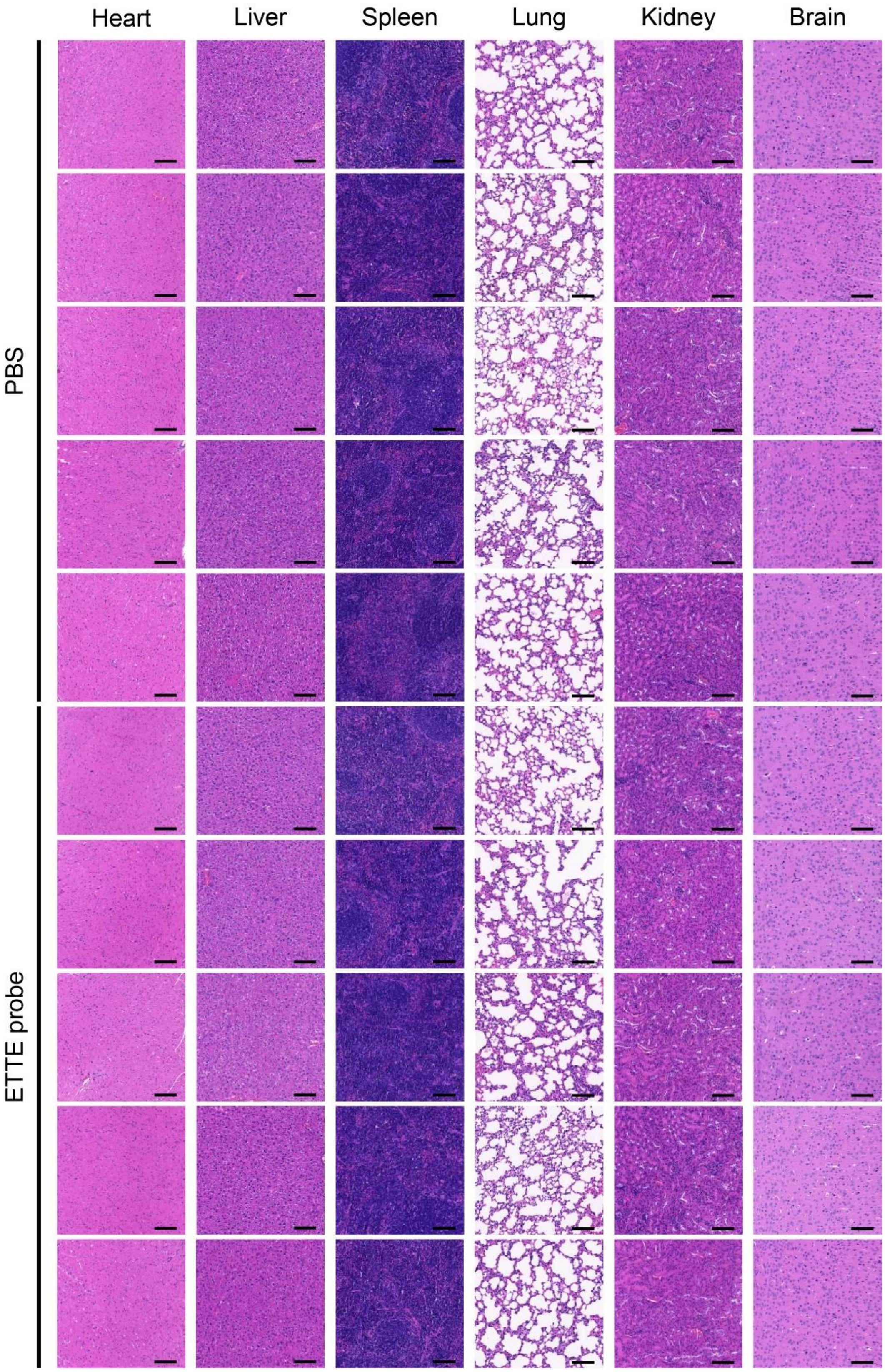


Supplementary Fig. 66. Histopathology images of dissected major organs (heart, liver, spleen, lung, kidney and Brain) stained with H&E of PBS-treated and ETTE probe-treated BALB/c nude mice. Scale bar: 100 μm, n = 5 mice.

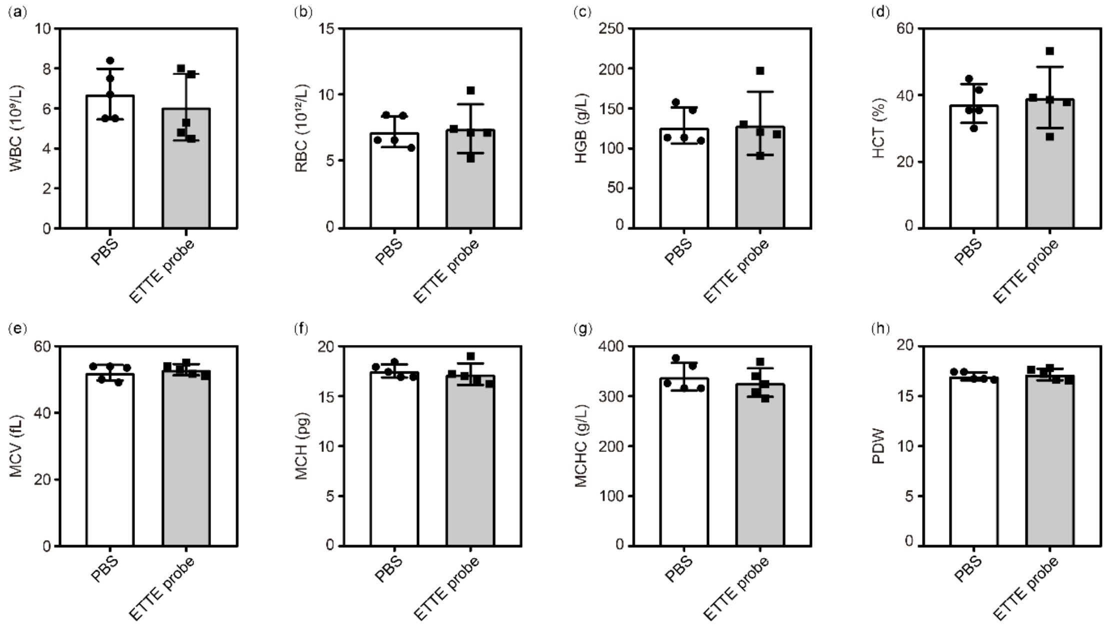


Supplementary Fig. 67. Hematological examination of the variation in WBC (white blood cell count), RBC (red blood cell count), HGB (hemoglobin), HCT (hematocrit), MCV (mean corpuscular volume), MCH (mean corpuscular hemoglobin), MCHC (mean corpuscular hemoglobin concentration) and PDW (platelet distribution width) after injections with PBS and ETTE probe. Data are presented as the mean values ± s.d.; unpaired two-tailed Student's t-test; n = 5 mice.

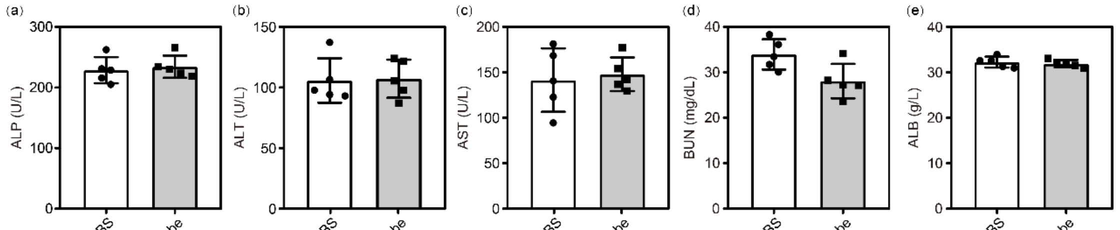


Supplementary Fig. 68. Blood biochemistry examination of the variation in ALP (alkaline phosphatase), ALT (alanine transferase), AST (aspartate transferase), BUN (blood urea nitrogen), and ALB (albumin) of BALB/c nude mice after injections with PBS and ETTE probe. Data are presented as the mean values ± s.d.; unpaired two-tailed Student's t-test; n = 5 mice.

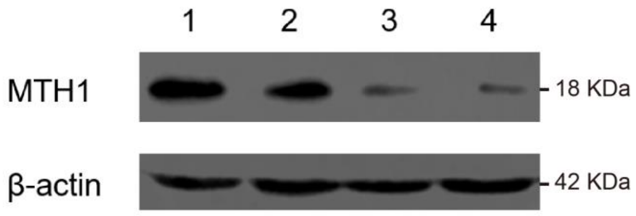


Supplementary Fig. 69. Western blot analysis on the expression of MTH1 and β-catenin protein in A549 cells after different treatments. A representative image of three biologically independent samples from each group is shown. Lane 1, untreated control; lane 2, transfected with 0.05 nmol siMTH1; lane 3, transfected with 0.15 nmol siMTH1; lane 4, transfected with 0.30 nmol siMTH1.

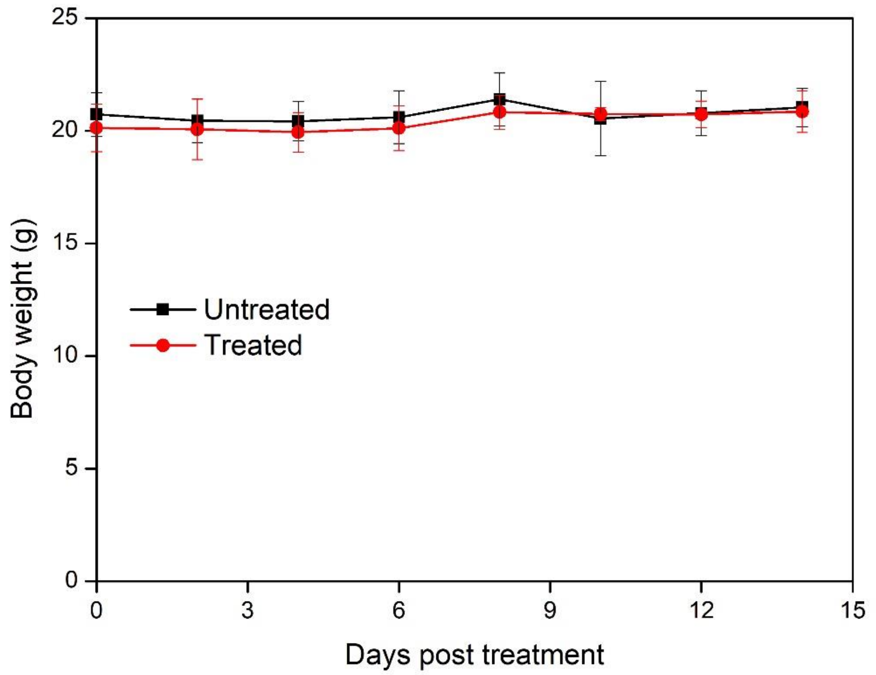


Supplementary Fig. 70. Graphical representation of the mean body weight from A549 xenograft mice treated without (black square) or with (red circle) MTH1 siRNA during 14 days. Each bar represents the mean values ± s.d.; n = 4 mice.

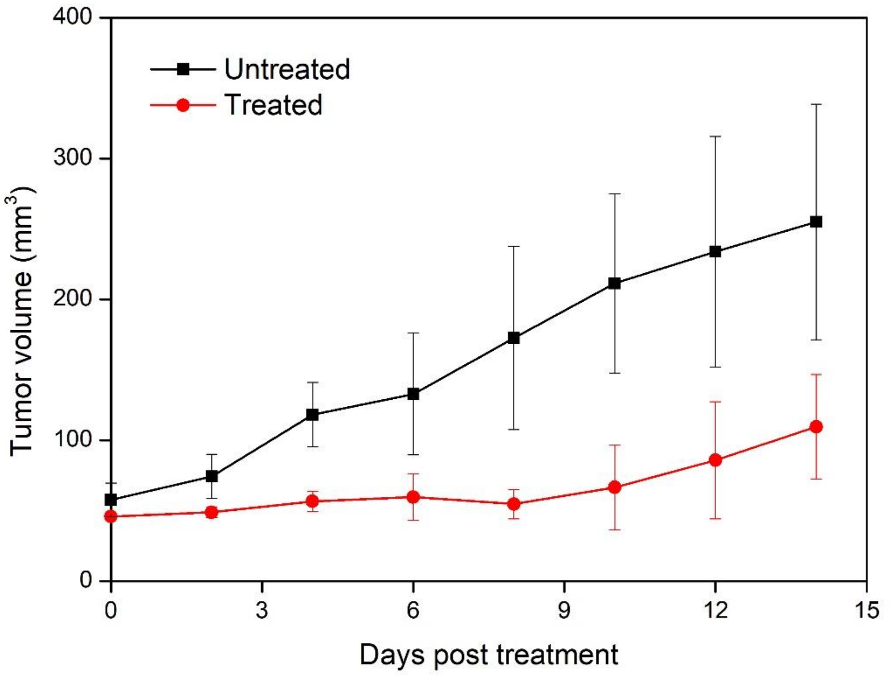


Supplementary Fig. 71. Graphical representation of the mean tumor volume from A549 xenograft mice treated without (black square) or with (red circle) MTH1 siRNA during 14 days. Each bar represents the mean values ± s.d.; n = 4 mice.

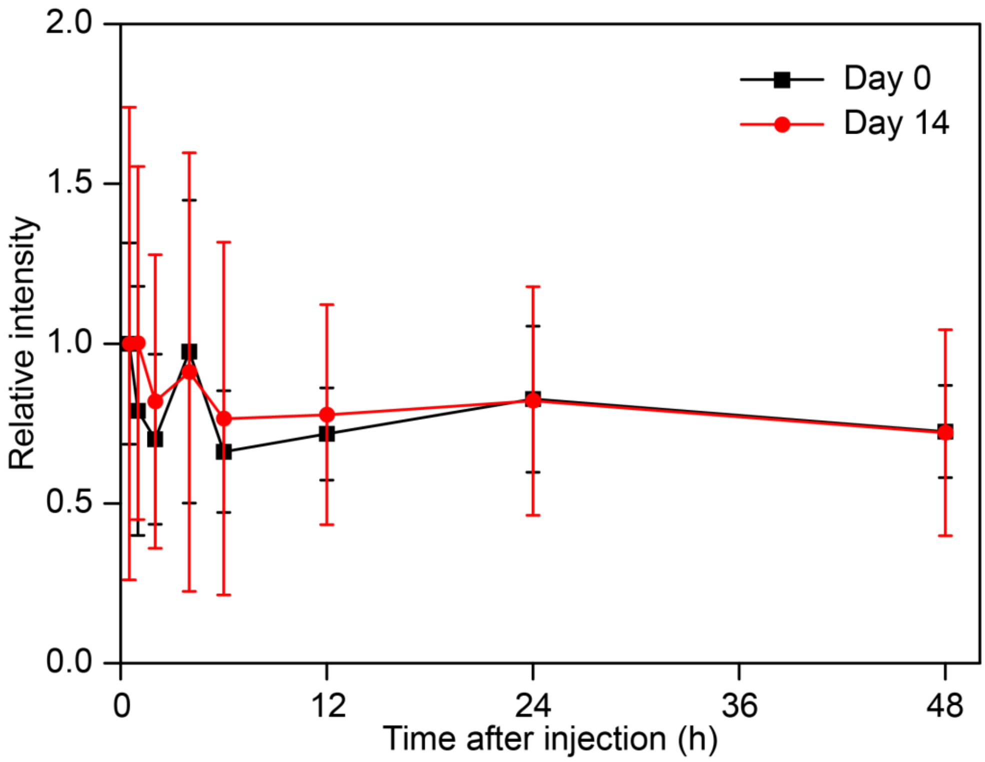


Supplementary Fig. 72. Corresponding quantitative data analysis of relative total radiance in tumor area of untreated group shown in Figure 6a after intravenous injection of ETTE nanoprobe without therapeutic agent at day 0 (black square) and day 14 (red circle) after treatment. Each bar represents the mean values ± s.d.; n = 4 mice.

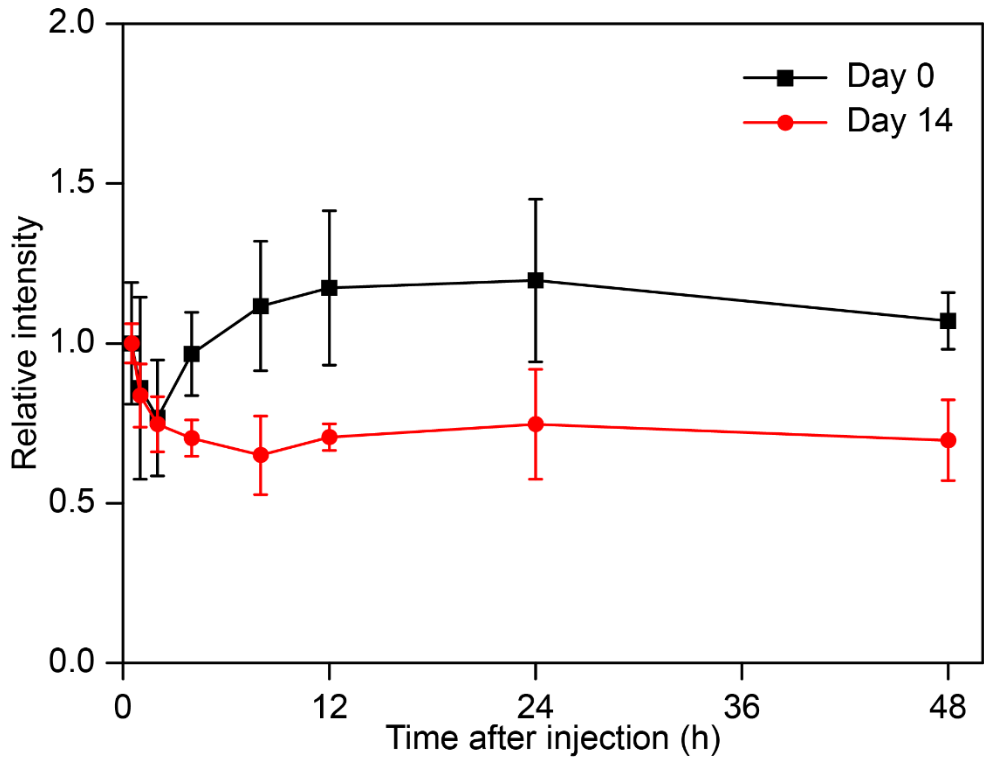


Supplementary Fig. 73. Corresponding quantitative data analysis of relative total radiance in tumor area of treated group shown in Figure 6c after intravenous injection of ETTE nanoprobe with therapeutic agent at day 0 (black square) and day 14 (red circle) after treatment. Each bar represents the mean values ± s.d.; n = 4 mice. Compared with the initial signal (black line), the relative afterglow signal intensity decreased significantly after 14 days treatment (red line).

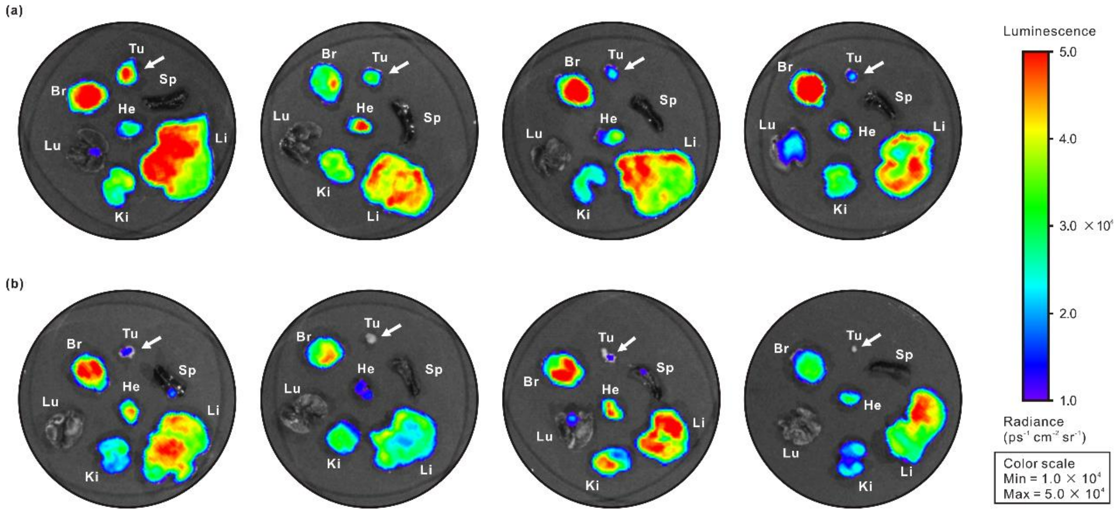


Supplementary Fig. 74. Ex vivo afterglow imaging of various organs and tumor tissue at the 48th hour after intravenous injection of ETTE nanoprobe without (a) and with therapeutic agent (b), respectively. Tu, tumor; Sp, spleen; Li, liver; Ki, kidney; Lu, lung; Br, brain; He, heart. Color scale: $1.0 \times 10^4 – 5.0 \times 10^4$; n = 4 mice.

## Supplementary Table

Supplementary Table 1. Oligonucleotides used in this study.

| **DNA** | **Sequence** [a] |
|---|---|
| DNA | TGGGTGGGTGGGGGGT |
| DNA-$Fe_3$ | [**Fe**]$_3$-TGGGTGGGTGGGGGGT |
| DNA-$Fe_6$ | [**Fe**]$_6$-TGGGTGGGTGGGGGGT |
| DNA-$Fe_9$ | [**Fe**]$_9$-TGGGTGGGTGGGGGGT |
| DNA-$Fe_{12}$ | [**Fe**]$_{12}$-TGGGTGGGTGGGGGGT |
| FAM-labeled DNA-$Fe_3$ | [**Fe**]$_3$-TGGGTGGGTGGGGGGT-FAM |
| FAM-labeled DNA-$Fe_6$ | [**Fe**]$_6$-TGGGTGGGTGGGGGGT-FAM |
| FAM-labeled DNA-$Fe_9$ | [**Fe**]$_9$-TGGGTGGGTGGGGGGT-FAM |
| FAM-labeled DNA-$Fe_{12}$ | [**Fe**]$_{12}$-TGGGTGGGTGGGGGGT-FAM |

[a] **Fe** represents nucleotides with ferrocenyl group.

Supplementary Table 2. List of sgRNA sequences for EGFR.

| **Name** | **Sequence** |
|---|---|
| EGFR-sgRNA-1-F | 5′-GGAATGGGTGAGTCTCTGTG-3′ |
| EGFR-sgRNA-1-R | 5′-CACAGAGACTCACCCATTCC-3′ |
| EGFR-sgRNA-2-F | 5′-AAAATTGGAGAAAATCTAAG-3′ |
| EGFR-sgRNA-2-R | 5′-CTTAGATTTTCTCCAATTTT-3′ |

**Supplementary References**

[1] Radhakrishnan, S. & Paul, S. Conducting polypyrrole modified with ferrocene for applications in carbon monoxide sensors. *Sens. Actuators B* **125**, 60-65 (2007).

[2] Zhang, L. et al. The marriage of ferrocene and silicotungstate: An ingenious heterogeneous Fenton-like synergistic photocatalyst. *Appl. Catal., B* **193**, 47-57 (2016).

[3] Orcutt, K.P. et al. Erlotinib-mediated inhibition of EGFR signaling induces metabolic oxidative stress through NOX4. *Cancer Res.* **71**, 3932-3940 (2011).

[4] Rusnak, D.W. et al. Assessment of epidermal growth factor receptor (EGFR, ErbB1) and HER2 (ErbB2) protein expression levels and response to lapatinib (Tykerb, GW572016) in an expanded panel of human normal and tumour cell lines. *Cell Prolif.* **40**, 580-594 (2007).

[5] Majumder, B. et al. Predicting clinical response to anticancer drugs using an ex vivo platform that captures tumour heterogeneity. *Nat. Commun.* **6**, 6169 (2015).

[6] Iida, M. et al. Overcoming acquired resistance to cetuximab by dual targeting HER family receptors with antibody-based therapy. *Mol. Cancer* **13**, 242 (2014).
