# Supplementary material for: Electron transfer-triggered imaging of EGFR signaling activity
Source: Nat Commun. 2022 Feb 1;13:594. doi: 10.1038/s41467-022-28213-y (PMC8807759; doi:10.1038/s41467-022-28213-y)
Supplement: Supplementary file 2 — Description of Additional Supplementary Files [file 41467_2022_28213_MOESM2_ESM.docx]

**Description of Additional Supplementary Files**

**Supplementary Movie 1:**

The dynamic afterglow intensity of ETTE nanoprobe during cell division. In the process of A549 cell division, the EGFR signaling pathway is activated, which enhances the response ability of ETTE nanoprobe. Monitoring the afterglow change of the ETTE nanoprobe within 1.5 hours continuously and dynamically by the laser confocal microscope, it can be observed that the afterglow intensity of the probe gradually increases as the cell division activity proceeds.
