# Supplementary material for: Electron transfer-triggered imaging of EGFR signaling activity
Source: Nat Commun. 2022 Feb 1;13:594. doi: 10.1038/s41467-022-28213-y (PMC8807759; doi:10.1038/s41467-022-28213-y)
Supplement: Supplementary file 4 — Reporting Summary [file 41467_2022_28213_MOESM4_ESM.pdf]

nature research

Corresponding author(s): Quan Yuan

Last updated by author(s): Nov 10, 2021

Reporting Summary

Nature Research wishes to improve the reproducibility of the work that we publish. This form provides structure for consistency and transparency in reporting. For further information on Nature Research policies, see our Editorial Policies and the Editorial Policy Checklist.

Statistics

For all statistical analyses, confirm that the following items are present in the figure legend, table legend, main text, or Methods section.

n/a	Confirmed	
☐	☒	The exact sample size (n) for each experimental group/condition, given as a discrete number and unit of measurement
☐	☒	A statement on whether measurements were taken from distinct samples or whether the same sample was measured repeatedly
☐	☒	The statistical test(s) used AND whether they are one- or two-sided<br>*Only common tests should be described solely by name; describe more complex techniques in the Methods section.*
☐	☒	A description of all covariates tested
☐	☒	A description of any assumptions or corrections, such as tests of normality and adjustment for multiple comparisons
☐	☒	A full description of the statistical parameters including central tendency (e.g. means) or other basic estimates (e.g. regression coefficient) AND variation (e.g. standard deviation) or associated estimates of uncertainty (e.g. confidence intervals)
☐	☒	For null hypothesis testing, the test statistic (e.g. F, t, r) with confidence intervals, effect sizes, degrees of freedom and P value noted<br>*Give P values as exact values whenever suitable.*
☒	☐	For Bayesian analysis, information on the choice of priors and Markov chain Monte Carlo settings
☒	☐	For hierarchical and complex designs, identification of the appropriate level for tests and full reporting of outcomes
☒	☐	Estimates of effect sizes (e.g. Cohen's d, Pearson's r), indicating how they were calculated

Our web collection on statistics for biologists contains articles on many of the points above.

Software and code

Policy information about availability of computer code

Data collection	1. QUANTUM ESPRESSO (version 6.8).<br>2. JES-FA200 EPR spectrometer.<br>3. The commercial software of FV500-IX81 confocal microscope.<br>4. The commercial software of CHI660D electrochemical working station.<br>5. IR spectrometer instrument (Thermo, Nicolet 6700).
Data analysis	Graphpad Prism (version 8.0.1.224), origin (2017), Image-Pro Plus (version 6.0.0.260), ImageJ software (NIH), Living Image (version 4.4), Shimadzu's LCMS Solution software (version 3.41)

For manuscripts utilizing custom algorithms or software that are central to the research but not yet described in published literature, software must be made available to editors and reviewers. We strongly encourage code deposition in a community repository (e.g. GitHub). See the Nature Research guidelines for submitting code & software for further information.

Data

Policy information about availability of data

All manuscripts must include a data availability statement. This statement should provide the following information, where applicable:

- Accession codes, unique identifiers, or web links for publicly available datasets
- A list of figures that have associated raw data
- A description of any restrictions on data availability

The experimental data supporting the findings of this study are available within the article and Supplementary Information. The data for all graphs generated in this study are provided in the Source Data file.

Field-specific reporting

Please select the one below that is the best fit for your research. If you are not sure, read the appropriate sections before making your selection.

- [x] Life sciences
- [ ] Behavioural & social sciences
- [ ] Ecological, evolutionary & environmental sciences

For a reference copy of the document with all sections, see nature.com/documents/nr-reporting-summary-flat.pdf

Life sciences study design

All studies must disclose on these points even when the disclosure is negative.

Sample size	In vitro studies were repeated at least three times independently and in vivo experiments with 4-5 mice per group were performed,meeting the standard of at least 3 biological replicates or 3 animals per group.
Data exclusions	No animals and/or data were excluded.
Replication	Experiments were repeated at least three independent experiments with similar results. All experiments were repeated and experimental findings were reproducible.
Randomization	For the in vivo studies, mice were randomized into the experimental groups with equivalent body weight.
Blinding	No blinding was carried out for data collection and analysis. No specific blinding was applied since all experiments were assigned into groups including relevant controls and analysis was done objectively and without bias.

Reporting for specific materials, systems and methods

We require information from authors about some types of materials, experimental systems and methods used in many studies. Here, indicate whether each material, system or method listed is relevant to your study. If you are not sure if a list item applies to your research, read the appropriate section before selecting a response.

Materials & experimental systems

n/a	Involved in the study	
[ ]	[x]	Antibodies
[ ]	[x]	Eukaryotic cell lines
[x]	[ ]	Palaeontology and archaeology
[ ]	[x]	Animals and other organisms
[x]	[ ]	Human research participants
[x]	[ ]	Clinical data
[x]	[ ]	Dual use research of concern

Methods

n/a	Involved in the study	
[x]	[ ]	ChIP-seq
[x]	[ ]	Flow cytometry
[x]	[ ]	MRI-based neuroimaging

Antibodies

Antibodies used	Anti-MTH1 antibody (#EPR15934-50, Abcam, UK); β-actin rabbit monoclonal antibody (AF5003; Beyotime); Horseradish peroxidase (HRP)-conjugated anti-IgG antibody (ARG65351, Arigo); Anti-EGFR (#4267, Cell Signaling Technology); Anti-p-EGFR (#3777, Cell Signaling Technology); Anti-Ki67 Rabbit pAb (#GB111499, Servicebio)
Validation	All antibodies were verified by the supplier and each lot has been quality tested. All validation statements can be found on the respective antibody website:<br>1. Anti-MTH1 antibody: https://www.abcam.com/mth1-antibody-epr15934-50-ab200832.html<br>2. β-actin rabbit monoclonal antibody: https://www.beyotime.com/product/AF5003.htm<br>3. Horseradish peroxidase (HRP)-conjugated anti-IgG antibody: https://www.arigobio.cn/Goat-anti-Rabbit-IgG-antibody-HRP-ARG65351.html<br>4. Anti-EGFR: https://www.cellsignal.cn/products/primary-antibodies/egf-receptor-d38b1-xp-rabbit-mab/4267?site-search-type=Products&N=4294956287&Ntt=%234267&fromPage=plp&_requestid=1768456<br>5. Anti-p-EGFR: https://www.cellsignal.cn/products/primary-antibodies/phospho-egf-receptor-tyr1068-d7a5-xp-rabbit-mab/3777?site-search-type=Products&N=4294956287&Ntt=%233777&fromPage=plp&_requestid=1768514<br>6. Anti-Ki67 Rabbit pAb: https://www.servicebio.cn/goodsdetail?id=3931

Eukaryotic cell lines

Policy information about cell lines

Cell line source(s)	A549 cells were derived from two different sources, American Type Culture Collection (ATCC) and China Center for Type

	Culture Collection (CCTCC). HT-29 cells, CAL-27 cells and MDA-MB-231 cells were purchased from China Center for Type Culture Collection (CCTCC).
Authentication	All cell lines were authenticated by ATCC and CCTCC using STR profiling.
Mycoplasma contamination	ATCC and CCTCC tested for mycoplasma contamination and none was detected.
Commonly misidentified lines (See ICLAC register)	No commonly misidentified lines were used.

Animals and other organisms

Policy information about studies involving animals; ARRIVE guidelines recommended for reporting animal research

Laboratory animals	Four-week-old female athymic BALB/c mice were purchased from the Hunan SJA Laboratory Animal Co., Ltd (Changsha, China)
Wild animals	No wild animals were used in the study.
Field-collected samples	The study did not involve samples collected from field.
Ethics oversight	All animals were used under protocols approved by the Institutional Animal Care and Use Committee of Hunan University (housing conditions, dark/light cycle: 12/12 h, temperature: 20°C, humidity: about 40%). The mice were also cultured under specific pathogen-free (SPF) condition at SPF Animal Laboratory of School and Hospital of Stomatology, Wuhan University. This experiment was approved by the Experimental Animal Ethics Committee of School and Hospital of Stomatology, Wuhan University (Ethics No. S07918100F).

Note that full information on the approval of the study protocol must also be provided in the manuscript.
